# Optical Control of Dopamine D2-like Receptors with Cell-Specific Fast-Relaxing Photoswitches

**DOI:** 10.1021/jacs.3c02735

**Published:** 2023-08-16

**Authors:** Belinda E. Hetzler, Prashant Donthamsetti, Zisis Peitsinis, Cherise Stanley, Dirk Trauner, Ehud Y. Isacoff

**Affiliations:** † Department of Chemistry, New York University, New York, New York 10003, United States; ‡ Molecular and Cell Biology, University of California, Berkeley, Berkeley, California 94720, United States; § Department of Chemistry and Department of Systems Pharmacology and Translational Therapeutics, University of Pennsylvania, Philadelphia, Pennsylvania 19104, United States; ⊥ Helen Wills Neuroscience Institute, University of California, Berkeley, California 94720, United States; || Weill Neurohub, University of California, Berkeley, Berkeley, California 94720, United States; # Molecular Biophysics & Integrated Bioimaging Division, Lawrence Berkeley National Laboratory, Berkeley, California 94720, United States

## Abstract

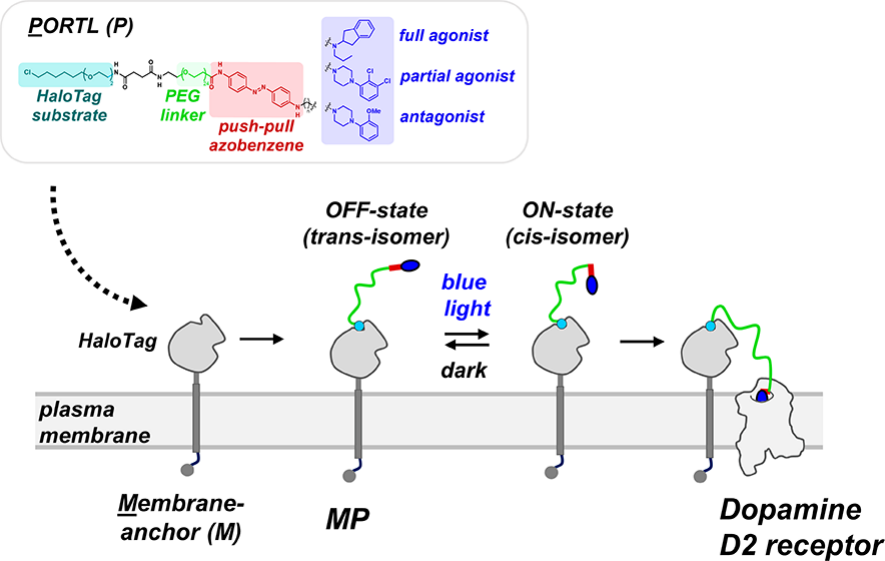

Dopamine D2-like receptors (D2R, D3R, and D4R) control diverse physiological and behavioral functions and are important targets for the treatment of a variety of neuropsychiatric disorders. Their complex distribution and activation kinetics in the brain make it difficult to target specific receptor populations with sufficient precision. We describe a new toolkit of light-activatable, fast-relaxing, covalently taggable chemical photoswitches that fully activate, partially activate, or block D2-like receptors. This technology combines the spatiotemporal precision of a photoswitchable ligand (P) with cell type and spatial specificity of a genetically encoded membrane anchoring protein (M) to which the P tethers. These tools set the stage for targeting endogenous D2-like receptor signaling with molecular, cellular, and spatiotemporal precision using only one wavelength of light.

## Introduction

The neuromodulator dopamine plays a central role in physiology and behavior (movement, learning, cognition, and metabolism),^1,2^ and dysregulation of dopamine signaling has been implicated in a variety of neuropsychiatric disorders.^3−5^ Dopaminergic signaling is mediated by five G-protein-coupled receptors (GPCRs) that are classified into two categories: the D1-like receptors (D1R and D5R), which couple to the stimulatory G proteins G_s/olf_, and the D2-like receptors (D2R, D3R, and D4R), which couple to the inhibitory G proteins G_i/o/z_.^6^ Among dopamine receptors, D2R is particularly notable as a therapeutic target. Motor impairments in Parkinson’s disease can be ameliorated with D2R agonists,^7^ antipsychotic medications are either D2R antagonists or weak partial agonists,^8−10^ and D2R is a putative target for treating addiction.^11^ Nevertheless, D2R medications have several features that lower the clinical efficacy, quality of life, and medication compliance. First, D2R is widely expressed in the central and peripheral nervous system, having distinct functions in different locations and cell types.^2,12^ Because D2R medications are freely diffusible, they cannot differentiate between the D2Rs that confer clinical benefit versus the D2Rs linked to adverse side effects. Second, once administered, D2R medications are persistently active until they are gradually metabolized or removed by the body, making it difficult to control the precise timing and level of D2R drug action at the target site. Finally, D2R medications bind off-target proteins, such as other GPCRs and ion channels.

The alternatives to conventional D2R medications are also limited in their utility *in vivo*. Genetic manipulation of D2R (knockout, knockdown, overexpression) occurs over long time scales and can lead to compensatory and possibly deleterious effects in neural circuits. Chemogenetic DREADDs (mutant GPCRs exclusively activated by designer ligands) and optogenetic opto-XRs (chimeras of a naturally light-sensitive opsin and a GPCR of interest) can be used to promote G protein signaling with cell type and spatiotemporal precision, but these tools are artificial proteins that cannot fully mimic the actions of endogenous receptors and their natural ligands.^13,14^ Membrane-anchored chemical ligands (DARTs) have been used to target AMPA and GABA_A_ receptors with tethered agonists and antagonists that limit action to genetically targeted cells and this approach could, in principle, be used to target endogenous D2Rs in specific locations.^15,16^ However, DARTs are chronically active, turning off gradually over hours to days as their protein component is removed by the cell, and thus, do not allow for temporal control to match the dynamics of dopamine signaling. LumiToxins (light-sensitive membrane-anchored peptides) can be turned on and off with light, but take minutes to turn off and are difficult to develop for dopamine receptors, which bind small chemical ligands.^17^ Taken together, there remains a great need for improved therapeutics that target D2R.^18^

Photopharmacology offers a means to toggle protein function on and off with precise spatiotemporal control, whereby a ligand’s affinity and/or efficacy for its endogenous biological target is modulated through a synthetic photoswitch. Remote control of D2R has been achieved with diffusible photocaged agonists based on dopamine itself and antagonists based on eticlopride or sulpiride (Figure S1A).^19−25^ However, photouncaging is irreversible, making it difficult to reliably and repeatedly turn on and off the target receptor, especially in living animals. In contrast, reversibly caged D2R modulators have been developed that contain photoswitches such as dithienylethenes or fulgimides.^20^ These are small molecules that undergo a reversible change from their thermodynamically stable *trans* state to their *cis* state within milliseconds in response to light, and then return to the *trans* state either in a light-driven manner or through thermal relaxation in the dark.^26^ Similarly, when appended to or incorporated within existing receptor ligands, azobenzenes can switch the ligands from the off-state to the on-state and back by reversibly distorting the ligand shape and ability to bind and activate or inhibit the target.

Proximity photopharmacology enables selective protein control with spatiotemporal and cell type specificity. In one version, the photoswitchable ligand is attached to an engineered version of the receptor via a self-labeling protein tag (e.g., SNAP-tag, CLIP-tag, or HaloTag) placed at a site in the receptor that is distal to the ligand binding site.^27−31^ This Photoswitchable Orthogonally Tethered Ligand (PORTL) has at one end an azobenzene that switches the receptor ligand between an on- and off-state, at the other end a moiety for covalent attachment to the protein tag (e.g., benzylguanine, benzylcytosine, haloalkane), and in the middle a polyethylene glycol (PEG) linker that allows the photoswitchable ligand to reach for its attachment point to the ligand binding site on the target protein. While this technology has enabled light-control of dopamine receptors and a variety of other proteins that are fused to a protein tag,^27,28,30,32−41^ insertion of the tag into the receptor requires modification of the receptor primary amino acid sequence, which could alter its function and requires either overexpression or genetic knock-in.

**Figure 1:**
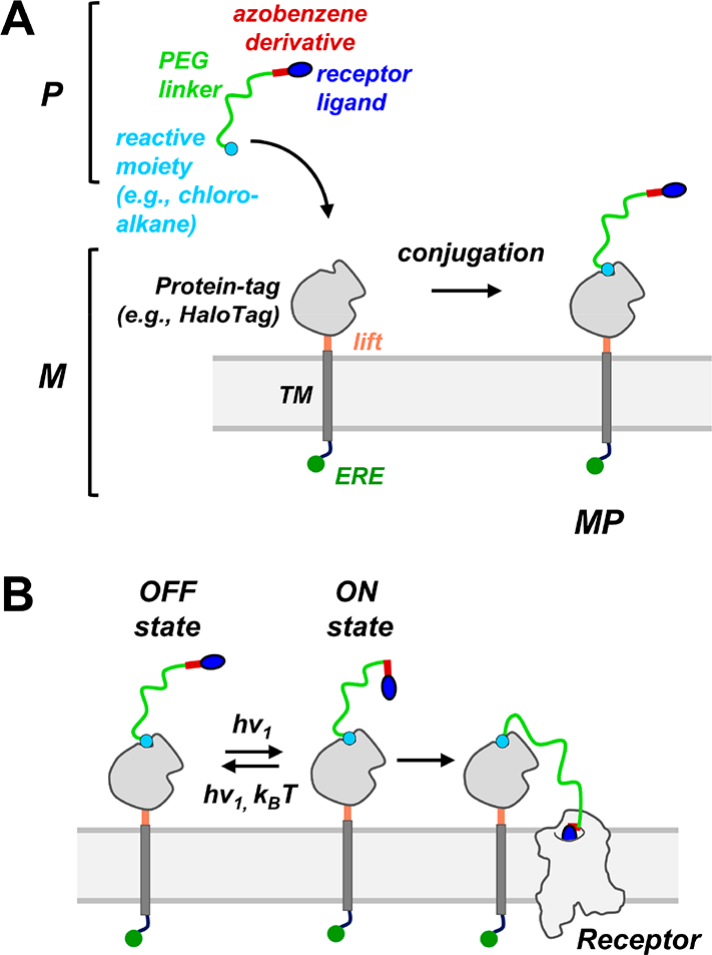
MP design. (A) MP is composed of two parts. The first component is the P, which contains a receptor ligand, a photoisomerizable azobenzene derivative, a long PEG linker, and reactive moiety. The second component is the “M”, which is a membrane-anchored protein tag that captures and restricts the P via the reactive moiety to a specific cell type and location. The M consists of a protein-tag that is anchored to the plasma membrane via a single-pass transmembrane segment (TM), a lift peptide that optimally positions the protein-tag at the cell surface, and an endoplasmic reticulum export signal (ERE) that boosts M expression at the cell surface. (B) By placing azobenzene close to or within the receptor ligand, the MP can be rapidly and reversibly driven by light between two states, one that has no or low affinity for the receptor (the off-state) and one that can bind the receptor (the on-state).

We recently solved this problem with our Membrane-anchored Photoswitchable Remotely Tethered Ligand (MP) approach, which combines the cell type-specific control of endogenous receptors afforded by DARTs with the temporal precision of photopharmacology.^29,40^ Instead of attachment to an engineered receptor, the PORTL (P) is covalently bound to an engineered membrane-anchor protein (M) that consists of an externally facing self-labeling protein tag fused to a single-pass transmembrane segment (Figure 1A). When the M is expressed in the target cell and conjugated to the P to form the MP (Figure 1A), the MP can interact with its endogenous receptor target by lateral diffusion in the plasma membrane (Figure 1B). Recently, we developed a two-wavelength (one wavelength to turn on and another wavelength to turn off) D1R/D5R-selective MP agonist called **MP-D1_ago_** that consists of a SNAP-tag M and a SNAP-reactive D1 PORTL agonist and applied its spatial, temporal, and cellular specificity to study the role of striatal D1Rs in the control of movement. Here, we extended the MP approach to the D2-like dopamine receptors using a new HaloTag membrane anchor. We generated three HaloTag-reactive (chloroalkane) D2 PORTLs: an agonist (**MP-D2_ago_**), a partial agonist **(MP-D2_p.ago_**), and an antagonist (**MP-D2_block_**). Importantly, by incorporating a red-shifted fast-relaxing azobenzene into the pharmacophores used in each PORTL, we obtained one-wavelength photocontrol of D2R, with a light-driven turn-on and a rapid turn-off in the dark. These novel MPs could be used to provide cell type-specific and spatiotemporally precise control of endogenous D2R *in vivo*.

## Results and Discussion

A variety of synthetic pharmacophores have been developed over the years to mimic the interaction of dopamine with its receptors in the orthosteric binding site.^42^ Recent progress has led to the development of bitopic compounds, which consist of a primary pharmacophore that binds the orthosteric binding site, a hydrophobic secondary pharmacophore that binds a secondary binding site, and an aliphatic linker that forms a bridge between the two pharmacophores.^43,44^ Importantly, the identity of the secondary pharmacophore and the length and substitution pattern of the aliphatic linker can influence receptor affinity, subtype selectivity, and signaling bias.^45−50^ While most of the privileged scaffolds for the bitopic D2R ligands show polypharmacology across other non-dopaminergic GPCRs, our MP approach will only render off-target activity to GPCRs of concern that are expressed on cells targeted by the M.^51,52^ The hydrophobic secondary pharmacophore stood out as an ideal unit for azologization, as previous work has shown that a fulgimide or diarylethene photoswitch can be incorporated at this position (Figure S1B).^20,53^ Furthermore, it has been shown that bitopic compounds can be elongated beyond the secondary pharmacophore with a remote fluorophore or a second ligand,^54−56^ hinting that an analogous extension with a PEG linker and covalent binding to the M anchor protein could be tolerated (Figure 1).

Parent azobenzene transitions from a thermostable *trans-*state to a higher energy *cis-*state in response to UV-A light and back to the *trans-*state in response to visible light or by slow thermal relaxation in the dark. The need for potentially toxic and poorly tissue penetrating UV light and two colors has sparked interest in photoswitches with greater biocompatibility. This led to the development of red-shifted, fast-relaxing azobenzenes through modification of their substitution pattern on their aromatic cores. For instance, through introduction of an electron-donating group in para-position to an electron-withdrawing group, the relaxation half-life of the photoswitch can be decreased from hours to milliseconds.^57,58^ Push–pull photoswitches are operated by one wavelength of light—the *trans-*state switches to the *cis-*state with visible light instead of UV light, and the *cis-*state rapidly relaxes back to the *trans-*state in the dark.^59^

An additional advantage of red-shifted, fast-relaxing azobenzenes is their photostationary state (PSS). Unsubstituted azobenzene is an isomeric mixture under visible light (∼95% *trans*: ∼5% *cis*) or UV-A light (∼20% *trans*: ∼80% *cis*), limiting the dynamic range of photoswitching.^60^ This limitation also applies to variety of other commonly used photoswitches (fulgi(mi)de, diarylethene, stilbene, hemithioindigo, spiropyrane and the LOV2 peptide).^61−67^ In contrast, since many azobenzenes are purely *trans* when left to relax for long enough in the dark,^57^ azobenzene variants that undergo this thermal relaxation rapidly provide the advantage of being able to toggle between light activated majority *cis* and fully *trans* in the dark. Therefore, if the *trans-*configuration was inert and the light-driven *cis*-configuration was bioactive, the photoswitch would avoid a finite floor of activation in the light-driven off state. This can be particularly useful in the MP approach, where the concentration of the genetically encoded M anchor protein may be difficult to control *in vivo*. Therefore, we based our design of the D2R photoswitches on a red-shifted, fast-relaxing azobenzene moiety that is flanked by an amine and amide (a push–pull azobenzene), analogous to that in a glutamate receptor photoswitch that we developed previously.^27,68^

## Synthesis and Functional Analysis of MP-D2_ago_

**Figure 2:**
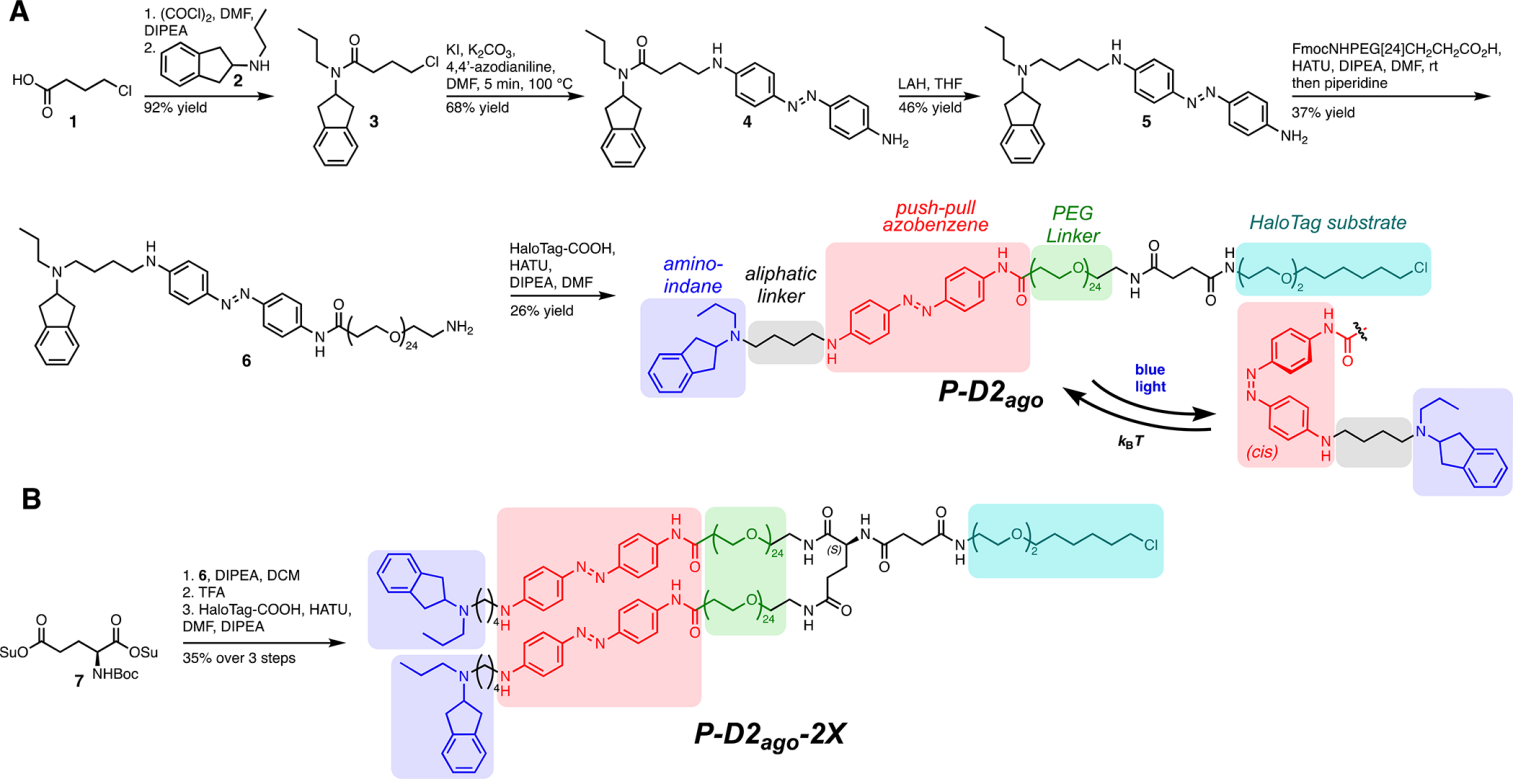
Synthesis of **P-D2**_**ago**_ and **P-D2**_**ago**_**-2X**. (A) Synthesis of **P-D2**_**ago**_. (B) Synthesis of branched, photoswitchable aminoindane dimer **P-D2**_**ago**_**-2X**.

In effort to generate an MP D2R agonist, we chose 2-aminoindane as a primary pharmacophore because it is a full D2R agonist that can tolerate chemical modification.^56,69,70^ A 4-carbon chain was installed by acylation of *N*-propyl 2-aminoindane (**2**) with freshly prepared acid chloride from acid **1** (Figure 2A). Upon Finkelstein reaction of **3**, the *in situ* formed primary iodide underwent S_N_2-reaction with commercial 4,4′-azodianiline. The reduction of disubstituted amide **4** to tertiary amine **5** proceeded in moderate yields but was tolerated by the azobenzene moiety. A heterobifunctional, carboxylic acid terminated PEG[24]-linker was then installed by a HATU-mediated coupling with azoaniline **5**, followed by the unveiling of the primary amine **6** by Fmoc-deprotection. Lastly, a HATU-mediated coupling with the chloroalkane substrate for the HaloTag (**HaloTag-COOH**) furnished the final construct **P-D2**_**ago**_.^71^ Motivated by the recent success of branched PORTLs bearing multiple photoswitchable glutamates in increasing the effective concentration and sensitivity to light, we also synthesized a branched analog of **P-D2_ago_** with two PEG[24]-azobenzene-2-aminoindanes (**P-D2**_**ago**_**-2X**, Figure 2B).^41^ This was achieved in a single operation by linking primary amine **6** with bis-NHS ester **7**, followed by Boc deprotection and amide coupling with the HaloTag carboxylic acid.

**Figure 3:**
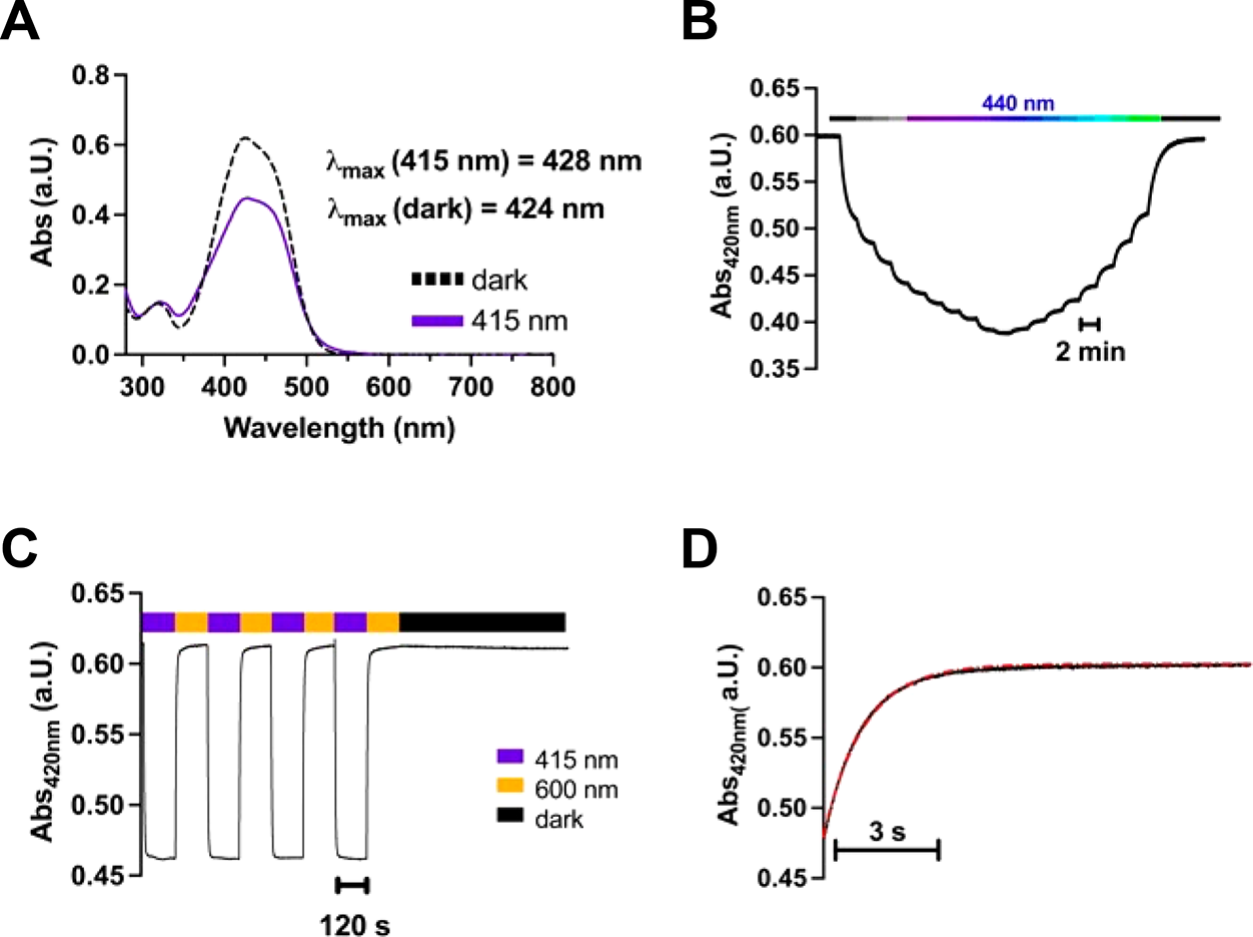
Photophysical characterization of **P-D2**_**ago**_. (A) UV Vis absorbance spectra of **P-D2**_**ago**_ (20 μM in DMSO, 24 °C) in the dark and under 415 nm irradiation. (B) Wavelength scan for **P-D2**_**ago**_ (20 μM, DMSO, 24 °C). Each wavelength is applied for 2 min. A maximum photostationary state (PSS) is reached by irradiation with 440 nm. (C) Reversible switching of **P-D2**_**ago**_ (20 μM, DMSO, 24 °C) by alternating irradiation with 415/600 nm, 90 s irradiations. (D) Thermal relaxation of **P-D2**_**ago**_ in DMSO (20 μM) after 1 min of irradiation with 415 nm at 37 °C. The thermal relaxation half-life of *cis* isomer is <1 s.

UV/vis studies confirmed the desired switching properties of **P-D2**_**ago**_: the absorption maximum of **P-D2**_**ago**_ in DMSO is 424 nm and maximal switching into the *cis*-configuration proceeds at 440 nm (Figure 3A, B; Figure S2). The *trans* isomer absorbance maximum shifted to 444 nm in 10% DMSO (Figure S2A). **P-D2**_**ago**_ can be reversibly switched without fatigue (Figure 3C) and thermally relaxes back into the *trans*-configuration with a half-life of 0.78 s in DMSO (Figure 3D). In aqueous environment (10% DMSO in PBS), the thermal back-relaxation proceeds more rapidly, exceeding the detection limit of the UV/vis spectrophotometer (Figure S2C). The photostationary state of **P-D2**_**ago**_ was determined by NMR (10 mM, DMSO-d6). Under *in situ* irradiation with a 415 nm high-power LED, 20% *cis* content was achieved (Figure S2D).

Our original M anchor protein contains an extracellularly facing SNAP-tag anchored to the plasma membrane via a single-pass transmembrane segment, a rigid α-helical “lift” peptide between the transmembrane segment and the SNAP-tag that optimizes the positioning of the SNAP-tag “above” the cell surface, and an intracellular endoplasmic reticulum export signal (ERE) that enhances surface expression.^29^ To provide orthogonality with our D1MP, we switched the SNAP-tag to the HaloTag, which reacts with **P-D2**_**ago**_ (Figure 1).

**Figure 4:**
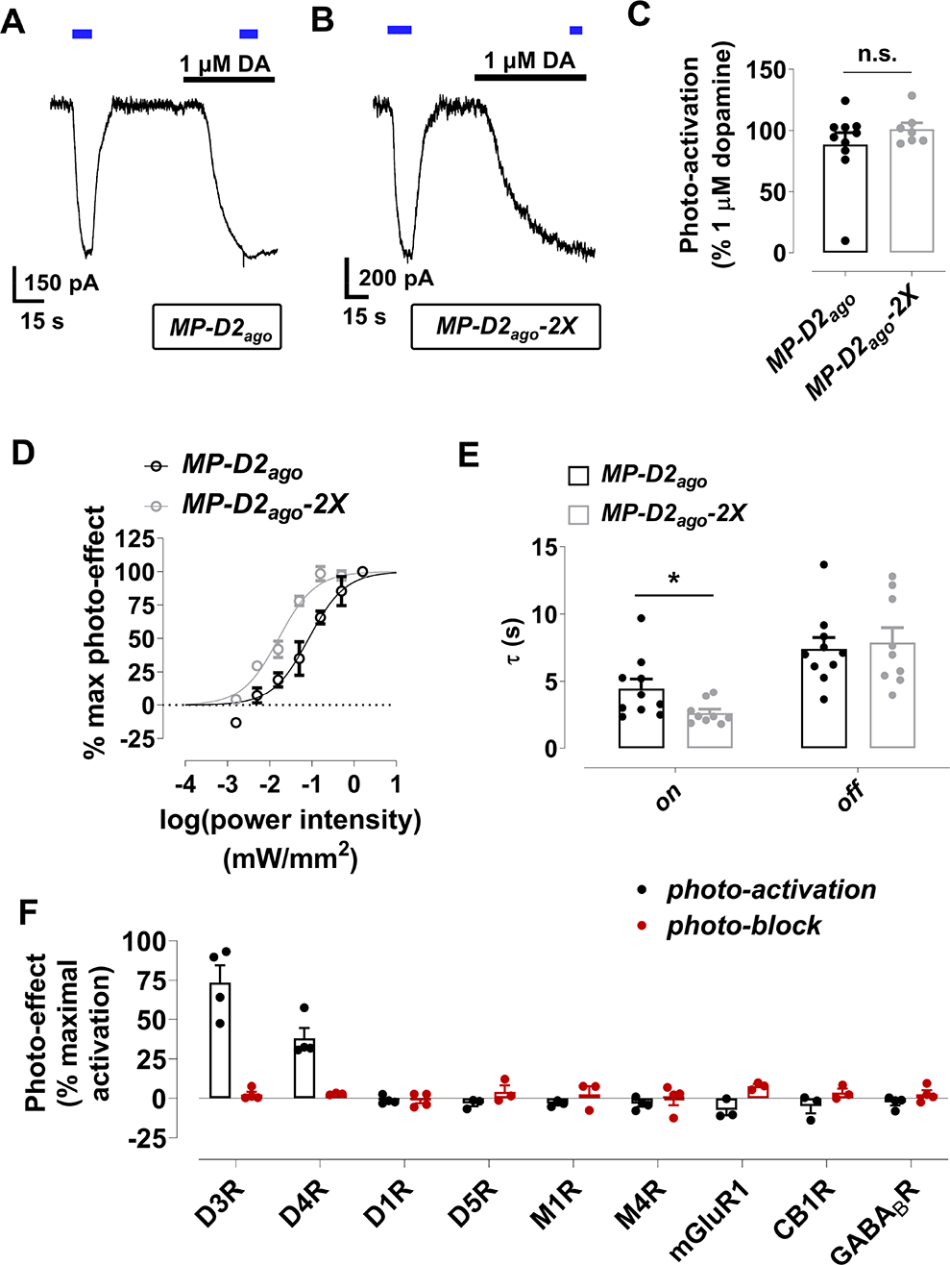
Photoactivation of D2R by **MP-D2**_**ago**_ and **MP-D2**_**ago**_**-2X**. (A, B) Representative traces of D2R activation by a photoagonist with one branch point, **MP-D2_ago_** (A) or two branch points, **MP-D2_ago_-2X** (B). (C) Summary of the maximal photoactivation of D2R by **MP-D2_ago_** or **MP-D2_ago_-2X** relative to a saturating concentration of dopamine (1 μM). Unpaired two-sided *t* test, *p* = 0.33, *n* = 10 cells for **MP-D2_ago_** and 7 cells for **MP-D2_ago_-2X**. (D) Summary of the light sensitivity of D2R activation in response to **MP-D2_ago_** or **MP-D2_ago_-2X**. (E) Summary of the on- and off-kinetics of D2R activation in response to **MP-D2_ago_** or **MP-D2_ago_-2X**. Unpaired two-sided *t* test, *p* = 0.03, *n* = 10 and 7 cells from left to right. (F) Summary of the maximal photoactivation and photoblock of various receptors by **MP-D2_ago_-2X** relative to a saturating concentration of agonist for each receptor. DA = dopamine.

We measured the effect of **P-D2**_**ago**_ tethered to the M (MP-D2_ago_) on D2R in HEK293T cells using a coexpressed G protein-activated inwardly rectifying K^+^ (GIRK) channel as an effector (Figure S3). Cells were cotransfected with D2R, the M, and GIRK and then labeled with **P-D2**_**ago**._ Following washout of unbound **P-D2**_**ago**_, the cells were patch-clamped in whole-cell configuration in high external K+ (120 mM), held at a negative holding potential (−80 mV), and switched from the dark to 440 nm (blue) light and then back again to the dark. Consistent with receptor activation and consequent opening of GIRK channels, blue light (to drive isomerization to the *cis* configuration) elicited a large inward current that was rapidly reversed by turning off the light (allowing return to the *trans* configuration) (Figure 4A). The magnitude of the light-driven current approached that elicited by a concentration of dopamine (1 μM) that fully saturates the receptor (89% of dopamine current; Figure 4A, C), indicating that MP-D2_ago_ is a near full D2R photoagonist.

We turned to the branched version of the photoswitch, **P-D2**_**ago**_**-2X**, which bears two azobenzene-2-aminoindanes photoagonists per M anchoring site. **P-D2**_**ago**_**-2X** tethered to the M (**MP-D2_ago_-2X**) maximally activated D2R (101% of dopamine current) and had no effect on dopamine-induced current (Figure 4B, C), indicating that it is a full photoagonist of D2R. Furthermore, MP-D2_ago_-2X photoactivated D2R with greater sensitivity to light (∼5-fold) and faster activation kinetics (∼2-fold) than the single photoagonist MP-D2_ago_ (Figure 4D, E), consistent with the expected higher effective concentration.

Consistent with the photophysical properties of its parent compound, **P-D2**_**ago**_ (Figure 3A, B), **P-D2**_**ago**_**-2X** maximally photoactivated D2R in response to 440 nm light (Figure S4A, B) in a manner that was rapid, reversible, and repeatable over multiple light exposures (Figure S4C, D). In addition, **P-D2**_**ago**_**-2X** had no effect on HEK293T cells lacking either D2R (Figure S4E, F) or the M (Figure S4G, H). Taken together, these results indicate that **MP-D2_ago_-2X** works as intended.

To be useful as a tool for controlling D2R, it is critical that **MP-D2_ago_-2X** be completely inactive in its *trans-*configuration in the dark. To measure the activity of **MP-D2_ago_-2X** in the dark, we applied the D2R inverse agonist spiperone, which is a competitive inhibitor that also suppresses constitutive receptor activity. As expected, in HEK293T cells expressing D2R and GIRK, spiperone elicited a reduction in *inward* current, consistent with inhibition of constitutive D2R activity (Figure S5A, B). Importantly, there was no difference between the effect of spiperone in the dark on cells that contained or lacked **MP-D2_ago_-2X** (Figure S5C), indicating that the photoswitch is completely inactive unless photoconverted to the *cis-*configuration with blue light.

We next characterized the binding of **P-D2**_**ago**_**-2X** to M by measuring the kinetics of complex formation. Using flow cytometry, we found that the M is fully labeled by 1 μM compound in just 10 min (Figure S6A), ∼4 times faster than the binding of our previous D1R photoswitch to SNAP-tag.^40^ We also measured the concentration dependence of **P-D2**_**ago**_**-2X** labeling. Interestingly, although flow cytometry indicated that 10 nM of **P-D2**_**ago**_**-2X** was required to approximately half-label the M (Figure S6B), only 1 nM of compound was needed to reach approximately half-maximal photoactivation of D2R in the GIRK assay (Figure S6C), suggesting that there was an excess of **P-D2**_**ago**_**-2X**, likely due to a combination of the inherent high affinity of the agonist moiety, 2-aminoindane, for D2R and an excess of M. This is in contrast to PORTLs developed previously for D1R and metabotropic glutamate receptors that require micromolar levels of compound to achieve full receptor activation under similar M expression and photoswitch labeling conditions.^27,40^**P-D2**_**ago**_**-2X** was about 10-fold more potent when labeling was extended from 1 h to overnight labeling (Figure S6C). The ability to work at low labeling concentrations could be particularly beneficial when **P-D2**_**ago**_**-2X** is applied to the brain *in vivo*.

We next compared the effect of **P-D2**_**ago**_**-2X** on D2R when attached to two different positions: (i) to the HaloTag in the M anchor protein of **MP-D2_ago_-2X**, where it is not physically attached to D2R (Figure 1), and must encounter it when M and the receptor diffuse into proximity of one another, and (ii) to a HaloTag that was genetically fused to the extracellular N-terminus of D2R (**HaloTag-D2R:P-D2_ago_-2X**) (Figure S7A), placing it in permanent close proximity to the receptor’s orthosteric binding site. Interestingly, the magnitude and kinetics of photoactivation of D2R by **P-D2**_**ago**_**-2X** in these two configurations were similar (Figure S7B–D). This indicates that the M density in the membrane is sufficient for the P to reach the target receptor.

We screened for the effect of **MP-D2_ago_-2X** on the other members of the D2-like receptor subfamily. **MP-D2_ago_-2X** was a strong partial photoagonist of D3R (Figure 4F), the closest homologue of D2R (78% identity), and a weak partial photoagonist of D4R (Figure 4F), which is less homologous (50% identity). In contrast to its effect on the D2-like receptors, **MP-D2_ago_-2X** had no effect on either D1-like receptor, D1R or D5R, nor on other receptors that are coexpressed with D2R in the same neurons in the brain, including selected muscarinic acetylcholine receptors, metabotropic glutamate receptors, cannabinoid receptors, and GABA receptors (Figure 4F). Some D2 antagonists and agonists have off-target actions on biogenic amine receptors such as 5HT2A serotonergic receptors and alpha adrenergic receptors. However, neither α_1A_AR nor α_2A_AR nor 5HT_2A_R is coexpressed with D2R in either the striatum or cortex, so that the cell-specific targeting of the MP-D2s can avoid such potential crosstalk.^72−75^ Thus, MP-D2_ago_-2X is a selective D2-like receptor photoagonist.

## Synthesis and Functional Analysis of MP-D2_p.ago_

**Figure 5:**
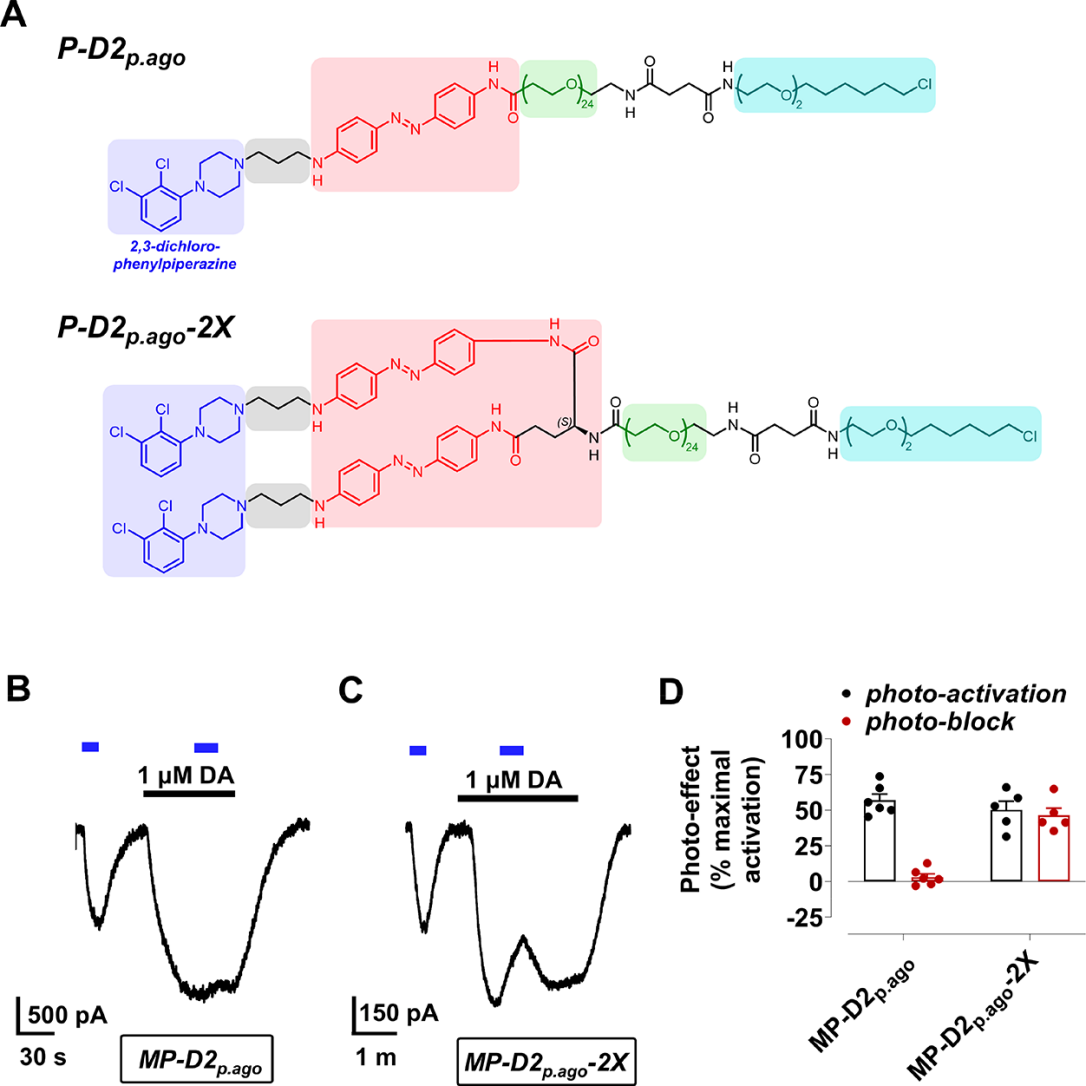
Partial photoactivation of D2R by **MP-D2**_**p.ago**_ and **MP-D2**_**p.ago**_**-2X**. (A) Structure of **P-D2**_**p.ago**_ and **P-D2**_**p.ago**_**-2X**. (B, C) Representative trace of D2R activation with a partial photoagonist with one branch point, **MP-D2_p.ago_** (B), or two branch points, **MP-D2_p.ago_-2X** (C). (D) Summary of the maximal photoactivation of D2R by **MP-D2_p.ago_** or **MP-D2_p.ago_-2X** relative to a saturating concentration of dopamine (1 μM). DA = dopamine.

To complement our cell-specific D2R photoagonist, we designed a tethered photopartial agonist of D2R. We employed the same photoswitch architecture used in **P-D2**_**ago**_ but replaced the 2-aminoindane headgroup with 2,3-dichloro-phenylpiperazine, which is present in a variety of D2R partial agonists including the antipsychotic medication aripiprazole.^76^ 2,3-dichloro-phenylpiperazine was appended to a fast-relaxing azobenzene, a 3-carbon aliphatic linker, a PEG[24] linker, and the chloroalkane HaloTag substrate, resulting in **P-D2**_**p.ago**_ (Figure 5A, top, and Figure S8A). In the GIRK assay, in response to blue light (to photoswitch to the *cis* configuration), **P-D2**_**p.ago**_ tethered to the M (**MP-D2_p.ago_**) partially activated D2R (57% of dopamine current), an effect that was reversed by switching back to the dark (allowing return to the *trans* configuration; Figure 5B, D).

**MP-D2_p.ago_** had almost no effect on saturating dopamine in response to blue light (3% reduction in dopamine current; Figure 5B, D), raising the possibility that the photoswitch is a full agonist at subsaturating concentrations rather than a true partial agonist that stabilizes a partially active conformational state in the receptor. To test this, we increased the effective concentration by synthesizing a branched variant with two 2,3-dichloro-phenylpiperazines per attachment point, resulting in **P-D2**_**p.ago**_**-2X** (Figure 5A, bottom and Figure S8B). **P-D2**_**p.ago**_**-2X** attached to the M (**MP-D2_p.ago_-2X**) partially activated the receptor to a similar degree as the monovalent analogue (50% of dopamine current) but also partially inhibited dopamine-induced receptor activation (46% reduction in dopamine current; Figure 5C, D). This is consistent with the actions of a partial agonist that can outcompete dopamine at high enough concentrations.

## Synthesis and Functional Analysis of MP-D2_block_

**Figure 6:**
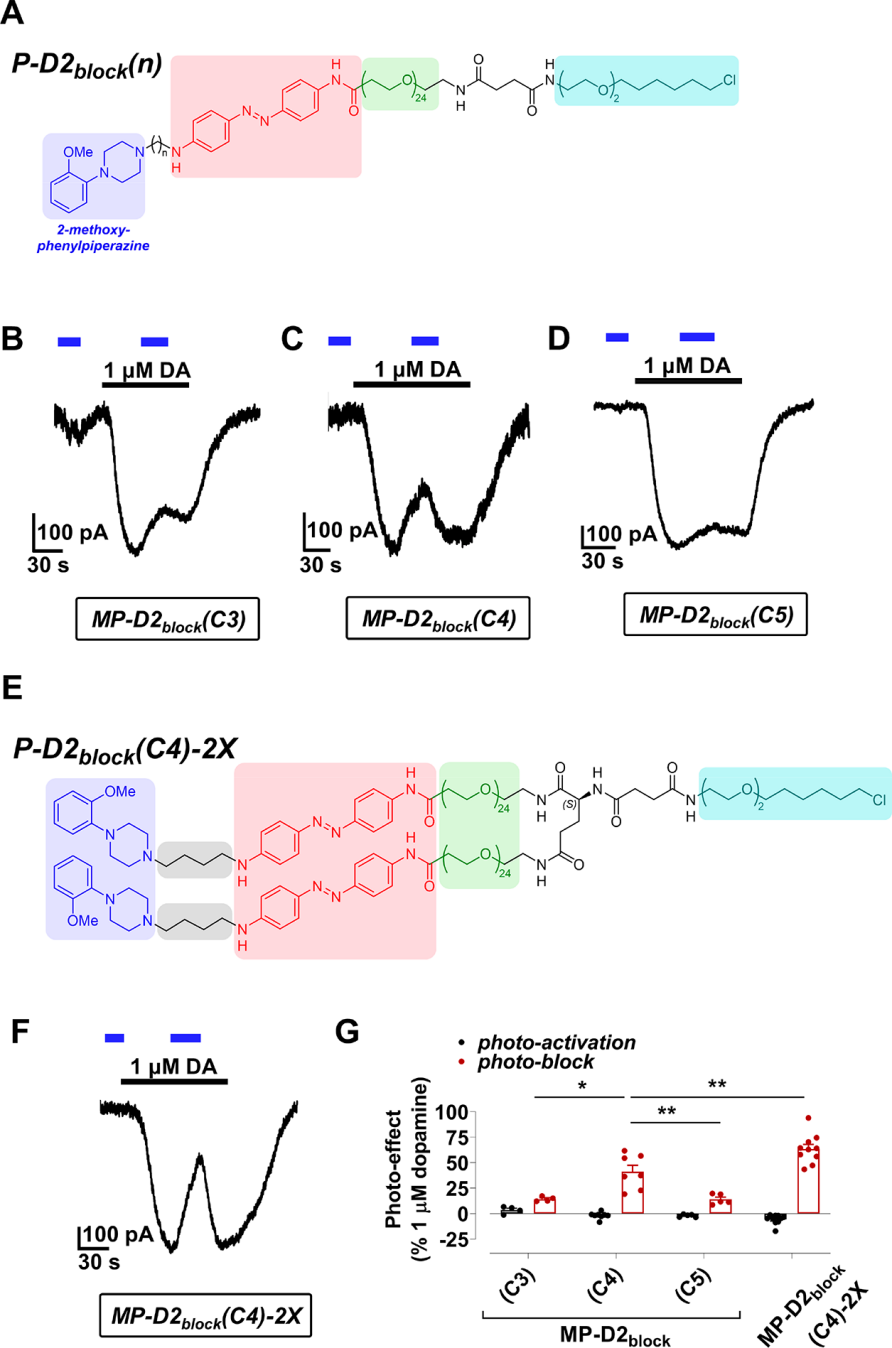
Photoantagonism of D2R by **MP-D2_block_** and **MP-D2**_**block**_**-2X**. (A) Structure of **P-D2**_**block**_**(C3)**, **P-D2**_**block**_**(C4)**, and **P-D2**_**block**_**(C5)**. (B–D) Representative trace of D2R activation by **MP-D2_block_(C3)** (B), **MP-D2_block_(C4)** (C), and **MP-D2_block_(C5)** (D). (E) Structure of **P-D2**_**block**_**(C4)-2X**. (F) Representative trace of D2R activation by **MP-D2_block_(C4)-2X**. (G) Summary of the maximal photoactivation of D2R by various **MP-D2_block_** variants relative to a saturating concentration of dopamine (1 μM). One-way ANOVA, *F* = 23.2, Tukey, *p < 0.5, **p < 0.01. *n* = 4, 7, 5, and 10 cells from left to right. DA = dopamine.

To develop a photoantagonist of D2R, we turned to the pharmacophore 2-methoxy-phenylpiperazine, which our previous work suggested to be much less efficacious than 2,3-dichloro-phenylpiperazine.^43^ We also tuned the length of the aliphatic linker between the 2-methoxy-phenylpiperazine and azobenzene, as we showed previously that subtle changes in this linker can dramatically impact activity.^43,48^ We synthesized 2-methoxy-phenylpiperazine analogs with a three-carbon linker, **P-D2**_**block**_**(C3)**, a four-carbon linker, **P-D2**_**block**_**(C4)**, or a five-carbon linker, **P-D2**_**block**_**(C5)** (Figure 6A and Figure S9). **P-D2**_**block**_**(C3)** tethered to the M, or **MP-D2_block_(C3)**, inhibited dopamine-induced D2R activation in the GIRK assay (14% reduction in dopamine current; Figure 6B, G) and weakly activated the receptor in the absence of dopamine (4% of dopamine current; Figure 6B, G), consistent with the fact that 2-methoxy-phenylpiperazine pharmacophore is itself a weak partial agonist.^43^ In contrast, **P-D2**_**block**_**(C4)** tethered to the M, or **MP-D2_block_(C4)**, more robustly inhibited the receptor than **MP-D2_block_(C3)** (41% reduction in dopamine current) and had no agonist activity (−1% of dopamine current; Figure 6C, G). This behavior is analogous to our previous finding that extension of the aliphatic linker can stabilize phenylpiperazines in an antagonist binding mode within the orthosteric binding site of the receptor.^43^**P-D2**_**block**_**(C5)** tethered to the M, or **MP-D2_block_(C5)**, was less effective than **MP-D2_block_(C4)** (∼14% reduction in dopamine current; Figure 6D, G), indicating that 4-carbons is the ideal aliphatic linker length for photoantagonism at D2R.

To enhance photoantagonism further, we synthesized a branched analogue of **P-D2**_**block**_**(C4)** with two 2-methoxy-phenylpiperazine moieties (**P-D2**_**block**_**(C4)-2X**) (Figure 6E). **P-D2**_**block**_**(C4)-2X** tethered to the M (**MP-D2_block_(C4)-2X**) enhanced photoblock ∼1.5-fold compared to its monovalent counterpart (63% reduction in dopamine current; Figure 6F, G).

In summary, we developed a toolkit of photoswitchable ligands to control D2R in a cell-specific manner. These include the full photoagonist **MP-D2_ago_-2X**, the partial photoagonist **MP-D2_p.ago_-2X**, and the photoantagonist **MP-D2_block_-2X**.

## Conclusion

Dopaminergic signaling spans a variety of physiological processes. The five dopamine receptors are expressed in diverse patterns in the CNS and periphery, making it challenging to pinpoint the exact role of each receptor in each cell type or neural circuit. While many approaches have been developed over the years to control a receptor of interest, none have provided control over endogenous receptors with cellular and spatiotemporal precision. The combination of membrane-anchored ligands for cell and spatial specificity with photopharmacology for precise temporal control provides a solution and offers a powerful means to study GPCR signaling.^77^ The tools described here complement our previously described tethered D1R photoagonist since they can be tagged to orthogonal fusion proteins (SNAP-tag versus HaloTag) and have distinct wavelength sensitivity, potentially allowing for multiplexed experiments in complex biological systems.

For *in vivo* use, the gene encoding the M component must be delivered to the desired brain region and cell type. In rodents, this can be accomplished with established genetic approaches, such as the Cre-lox system, that enable ectopic expression of the M protein in specific neurons.^78^ For example, we recently targeted striatal D1R-expressing direct-pathway medium spiny neurons (dMSNs) by injecting mice expressing Cre recombinase in this cell type (D1-Cre mice) with a Cre-dependent adeno-associated virus (AAV) encoding the SNAP-tag version of M that goes with P-D1_ago_.^40^ Along these lines, the HaloTag version of M developed here for the P-D2 switches can be targeted to D2R-expressing neurons in the striatum, including indirect pathway-medium spiny neurons (iMSNs) using A2a-Cre mice, or to cholinergic interneurons (ChIs) using ChAT-Cre mice, or to dopamine neurons that innervate the striatum using DAT-Cre mice.^79,80^ Once the M is expressed, a canula is implanted into the brain and used to infuse the photochemical P and guide the placement of the optical fiber to the target area, thus confining the photocontrol in three ways: via local delivery of the virus encoding M and the P, as well as by local illumination.

Several technical advancements could further increase the functionality of the MP system. The delivery of both the AAV encoding the M gene and the P would be considerably simplified by versions that cross the blood-brain barrier (although this would then mean that confinement of control to the brain region relies more heavily on spatial distribution of light from the optical fiber). To optimize expression of M, one can adjust the AAV titer or boost M cell surface targeting with ER export signals. In the future, it will be interesting to concentrate M in presynaptic terminals or postsynaptically using specific subcellular targeting motifs to boost M density and increase the specificity of the modulation. In addition, while tethering the P to the M protein in specific cells avoids unwanted effects in off-target cells, it could still bind off-target receptors in those target cells. This could be solved through the substitution of photoligands that bind in the orthosteric site with more selective allosteric ligands.^81^ It is also interesting to consider development of biased P variants that selectively activate specific signaling proteins downstream of D2R (e.g., via G proteins versus arrestins), since cellularly and spatiotemporally precise control of one such pathway at a time could greatly enhance our understanding of the relationship between this receptor and behavior. Such an advance would require the development of new assay systems that can detect the activation of specific signaling proteins in response to MP stimulation with light. Finally, our D2R MPs could be combined with neuronal activity reporters to enable the simultaneous control of dopamine receptor signaling and detection of neural activity. This challenge has been partly solved by the advent of reporters for calcium, voltage, or neural signal release (e.g., dopamine and glutamate) that operate in red/infrared light and are thus orthogonal to our blue-light sensitive D2R MPs.^82,83^

The MPs developed here have the potential to help uncover the role of D2R in various dopamine-associated diseases and could potentially be used as precision therapeutics. For example, Parkinson’s disease is a movement disorder that results from the degeneration of dopamine neurons that project from the substantia nigra compacta to the striatum, leading to the under-activation of striatal dopamine receptors that control movement.^84^ Dopamine replacement with D2R agonists (e.g., pramipexole) is a standard-of-care for treatment of Parkinson’s disease.^84,85^ However, as classical drugs, D2R agonists have two major problems: they reduce therapeutic efficacy and increase adverse side effects. First, they bind on- and off-target proteins beyond the striatum. Second, it is difficult, if not impossible, to apply the ideal therapeutic dose of D2R agonists once they are administered and metabolized by the body. These problems could be solved by targeting **MP-D2_ago_-2X** specifically to the striatum and fine-tuning its dose using light to mimic the actions of the dopamine that is lost in Parkinson’s disease. In addition to Parkinson’s disease, **MP-D2_p.ago_-2X** and/or **MP-D2_block_-2X** could potentially be used to treat schizophrenia by targeting therapeutically relevant D2Rs in the brain, thereby avoiding off-target effects in both the central nervous system and peripheral organs (e.g., pancreas, heart, gut).

## Supporting Information Available

Additional experimental details, materials, and methods, including supporting electrophysiology experiments, chemical design, synthesis and analysis (PDF)
