# Supplementary material for: Optical Control of Dopamine D2-like Receptors with Cell-Specific Fast-Relaxing Photoswitches
Source: J Am Chem Soc. 2023 Aug 16;145(34):18778–88. doi: 10.1021/jacs.3c02735 (PMC10472511; doi:10.1021/jacs.3c02735)
Supplement: Supplementary file 1 — ja3c02735_si_001.pdf [file ja3c02735_si_001.pdf]

Supporting Information

Optical Control of Dopamine D2-like Receptors with Cell-Specific Fast-Relaxing Photoswitches

Belinda E. Hetzler, Prashant Donthamsetti, Zisis Peitsinis, Cherise Stanley,

Dirk Trauner and Ehud Y. Isacoff

Contents

Additional experimental details, materials, and methods, including supporting electrophysiology experiments, chemical design, synthesis and analysis.

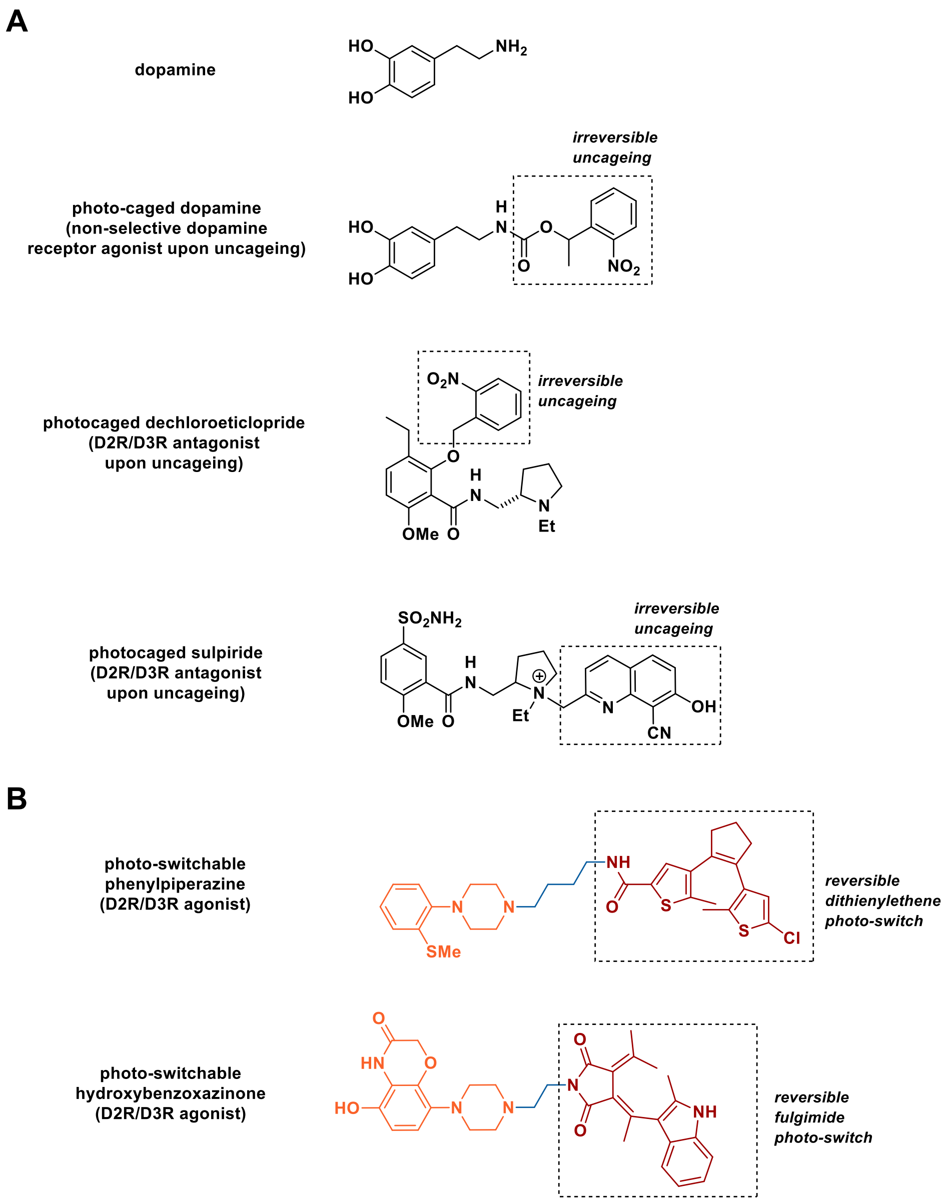

Figure S1. Existing photo-pharmacological tools for D2R. (**A**) Dopamine (*top*) is a catecholamine that binds in the orthosteric binding site of its receptors. Shown is a photo-caged agonist (*middle*) and two photo-caged antagonists (*bottom two*) that can be used to control D2R.[1,2] Importantly, the uncaging of these compounds in response to light is irreversible. (**B**) Bitopic ligands for D2R consist of a primary pharmacophore that binds the orthosteric binding site of the receptor, a secondary pharmacophore that binds a secondary binding site, and an aliphatic linker that connects the two pharmacophores. Shown is an example of a reversible D2R/D3R agonist that has a dithienylethene or a fulgimide photo-switch as the secondary pharmacophore.[3]

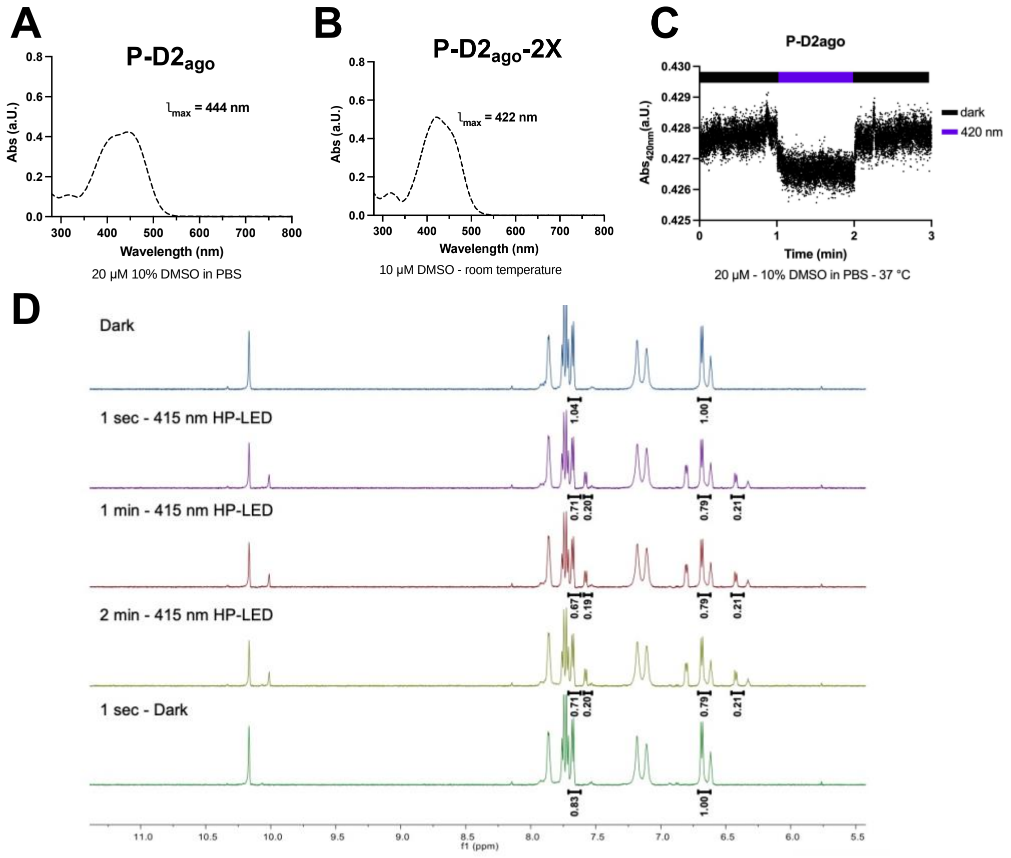

Figure S2. Extended photo-physical analysis. UV Vis absorption spectra of **P-D2$_{ago}$** and **P-D2$_{ago}$-2X** (**A**, **B**). Dark-adapted (100% *trans*) samples, recorded in DMSO (**B**) or 10% DMSO in PBS (**A**) at 24 °C. Samples were prepared at 20 µM (**A**), or 10 µM for the branched variant (**P-D2$_{ago}$-2X)** (**B**). Switching of **P-D2$_{ago}$** (**C**) in solution (10% DMSO in PBS,20 µM, 37 °C) with 420 nm (60 second irradiation). The thermal back-relaxation only allows switching to a scarcely detectable level by the UV Vis spectrophotometer. PSS determination by NMR (**D**). Irradiation of **P-D2$_{ago}$** in an NMR tube (5 mm tube, 10 mM, DMSO-d6) with a 415 nm HP-LED achieved a *cis*-content of 22%.

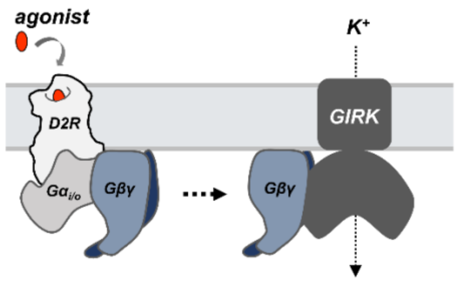

Figure S3. Schematic of the D2R-mediated GIRK activation assay. HEK293T cells were co-transfected with D2R and GIRK1(F137S). Agonist-induced receptor activation results in the release of Gβγ from endogenous heterotrimeric $G_{i/o}$. Gβγ subsequently binds GIRK channels, resulting in channel opening and increased inward-current assessed by whole-cell patch clamp recordings.

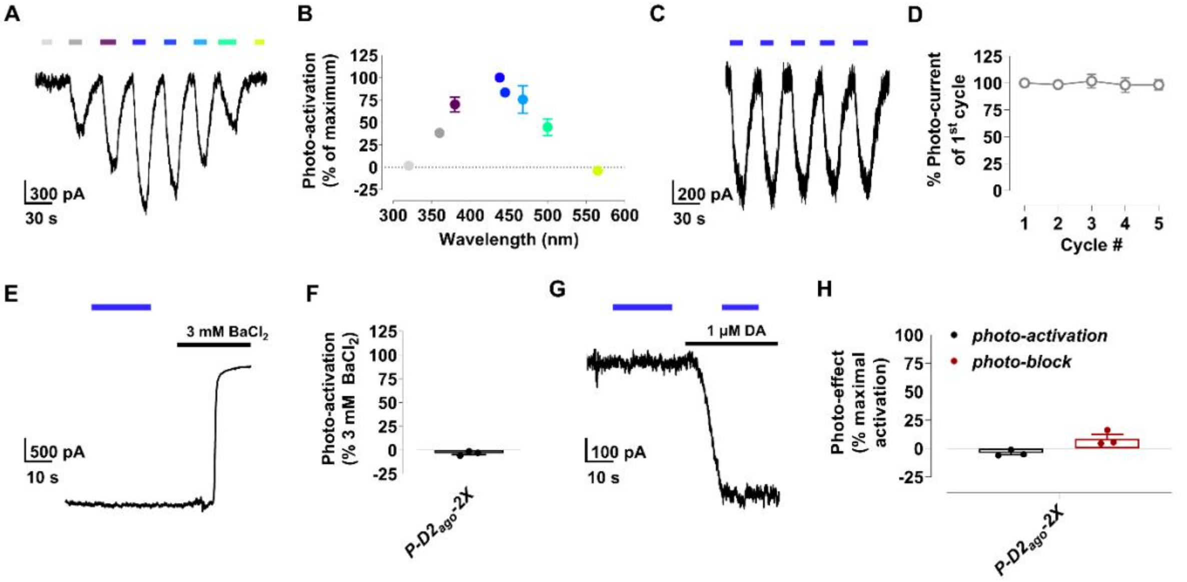

Figure S4. Additional characterization of MP-D2$_{ago}$-2X. (**A**) Representative trace of photo-activation of D2R by MP-D2$_{ago}$-2X in response to increasing wavelengths of light in the GIRK activation assay. (**B**) Summary of D2R photo-activation by MP-D2$_{ago}$-2X in response to increasing wavelengths of light. n = 3 cells per wavelength. (**C**) Photo-activation of D2R by MP-D2$_{ago}$-2X in response to 440 nm (blue) light is rapid, reversible, and repeatable. (**D**) Summary of the magnitude of D2R activation by MP-D2$_{ago}$-2X in response to repeated pulses of blue light. n = 4 cells. (**E**) Representative trace of the effect of blue light on M- and GIRK-expressing HEK293T cells labeled with **P-D2$_{ago}$-2X**. $BaCl_2$ was used to block GIRK channels and confirm channel expression. (**F**) Summary of the effect of blue light on M- and GIRK-expressing HEK293T cells labeled with **P-D2$_{ago}$-2X**. n = 3 cells. (**G**) Representative trace of the effect of blue light on D2R- and GIRK-expressing HEK293T cells labeled with **P-D2$_{ago}$-2X**. DA = dopamine. (**H**) Summary the effect of blue light on D2R- and GIRK-expressing HEK293T cells labeled with **P-D2$_{ago}$-2X** in the absence or presence of saturating 1 μM dopamine (photo-activation and photo-block, respectively). n = 3 cells.

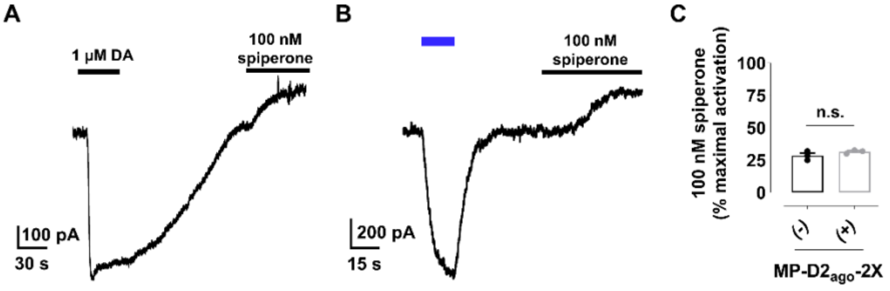

Figure S5. MP-D2$_{ago}$-2X has no effect on D2R in the dark. (**A**) Representative trace of the effect of dopamine followed by the inverse agonist spiperone on D2R-expressing cells in the GIRK activation assay. DA = dopamine. (**B**) Representative trace of the effect of MP-D2$_{ago}$-2X in response to blue light followed by spiperone on D2R. (**B**) Summary of the effect of spiperone on D2R in the absence of and presence of MP-D2$_{ago}$-2X. Unpaired two-sided t-test, p = 0.24, n = 3 cells per condition.

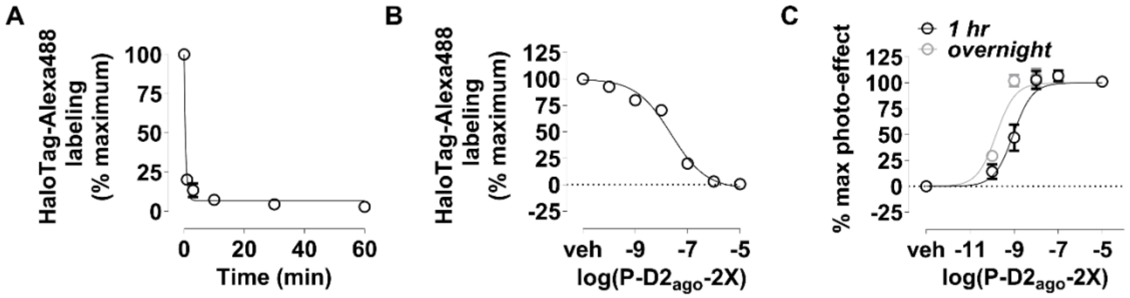

Figure S6. The relationship between P-D2$_{ago}$-2X labeling of the M and the photo-activation of D2R by MP-D2$_{ago}$-2X. (**A**) The kinetics of 1 µM binding of **P-D2$_{ago}$-2X** to M, according to its ability to block the dye HaloTag-Alexa488 from binding to the HaloTag-tag of the M in the flow cytometry assay. The M was first labeled with **P-D2$_{ago}$-2X** and then incubated with the dye. n = 2 replicates per time point. (**B**) Concentration-dependence of **P-D2$_{ago}$-2X** binding to the M in the flow cytometry assay. n = 3 replicates per concentration. (**C**) Photo-activation of D2R by MP-D2$_{ago}$-2X in the GIRK assay with increasing concentrations of **P-D2$_{ago}$-2X** after 1 hour or overnight labeling (ON). n = 3-7 cells per concentration.

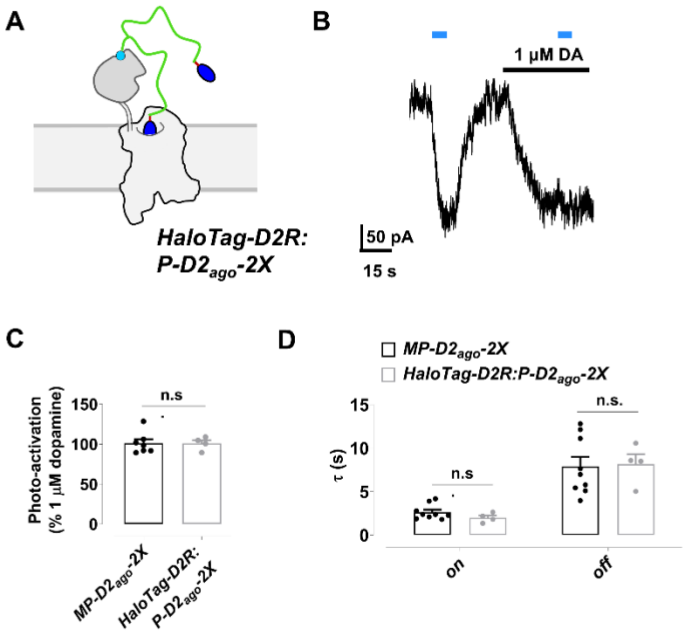

Figure S7. A comparison of the effect of MP-D2$_{ago}$-2X on D2R *versus* the effect of P-D2$_{ago}$-2X when tethered to HaloTag-D2R (HaloTag-D2R:P-D2$_{ago}$-2X). (**A**) A schematic representation of a D2R variant with HaloTag fused the extracellular N-terminus of the receptor. **P-D2$_{ago}$-2X** is shown tethered to the HaloTag (HaloTag-D2R:P-D2$_{ago}$-2X), with one of two agonist moieties bound to the orthosteric binding site of the receptor. (**B**) Representative trace of the photo-activation of HaloTag-D2R:P-D2$_{ago}$-2X in response to blue light. DA = dopamine. (**C**) Comparison of the maximal photo-activation of D2R by MP-D2$_{ago}$-2X and HaloTag-D2R:P-D2$_{ago}$-2X. Unpaired two-sided t-test, $p = 0.98$, $n = 7$ cells for MP-D2$_{ago}$-2X and 4 cells for HaloTag-D2R:P-D2$_{ago}$-2X. (**D**) Comparison of the activation and deactivation kinetics of D2R by MP-D2$_{ago}$-2X and HaloTag-D2R:P-D2$_{ago}$-2X. One-way ANOVA, $F = 13.41$, Tukey post-hoc test, $n = 9$ cells for MP-D2$_{ago}$-2X and 4 cells for HaloTag-D2R:P-D2$_{ago}$-2X.

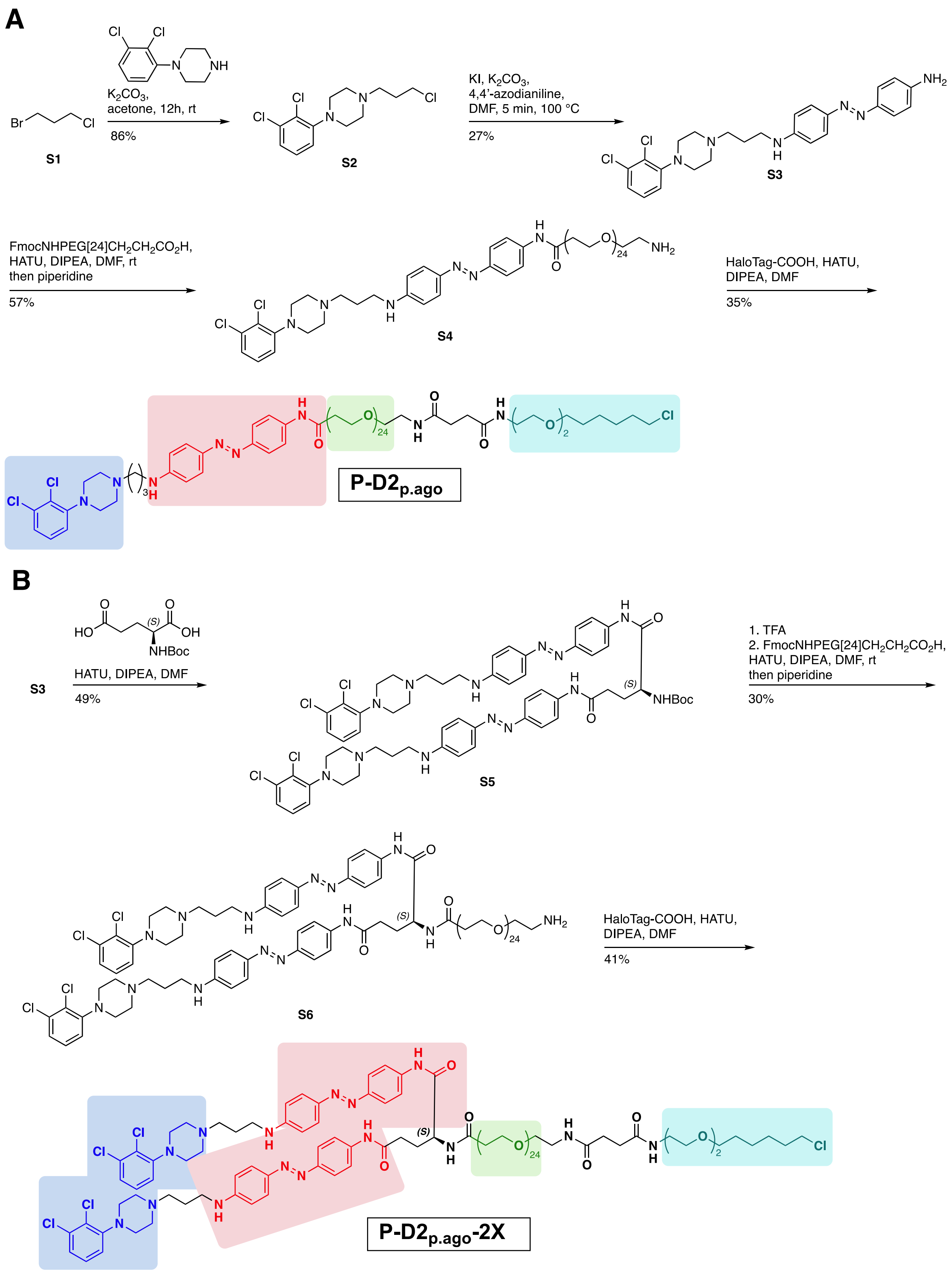

Figure S8. Synthesis of P-D2$_{p.ago}$ (A) and P-D2$_{p.ago}$-2X (B).

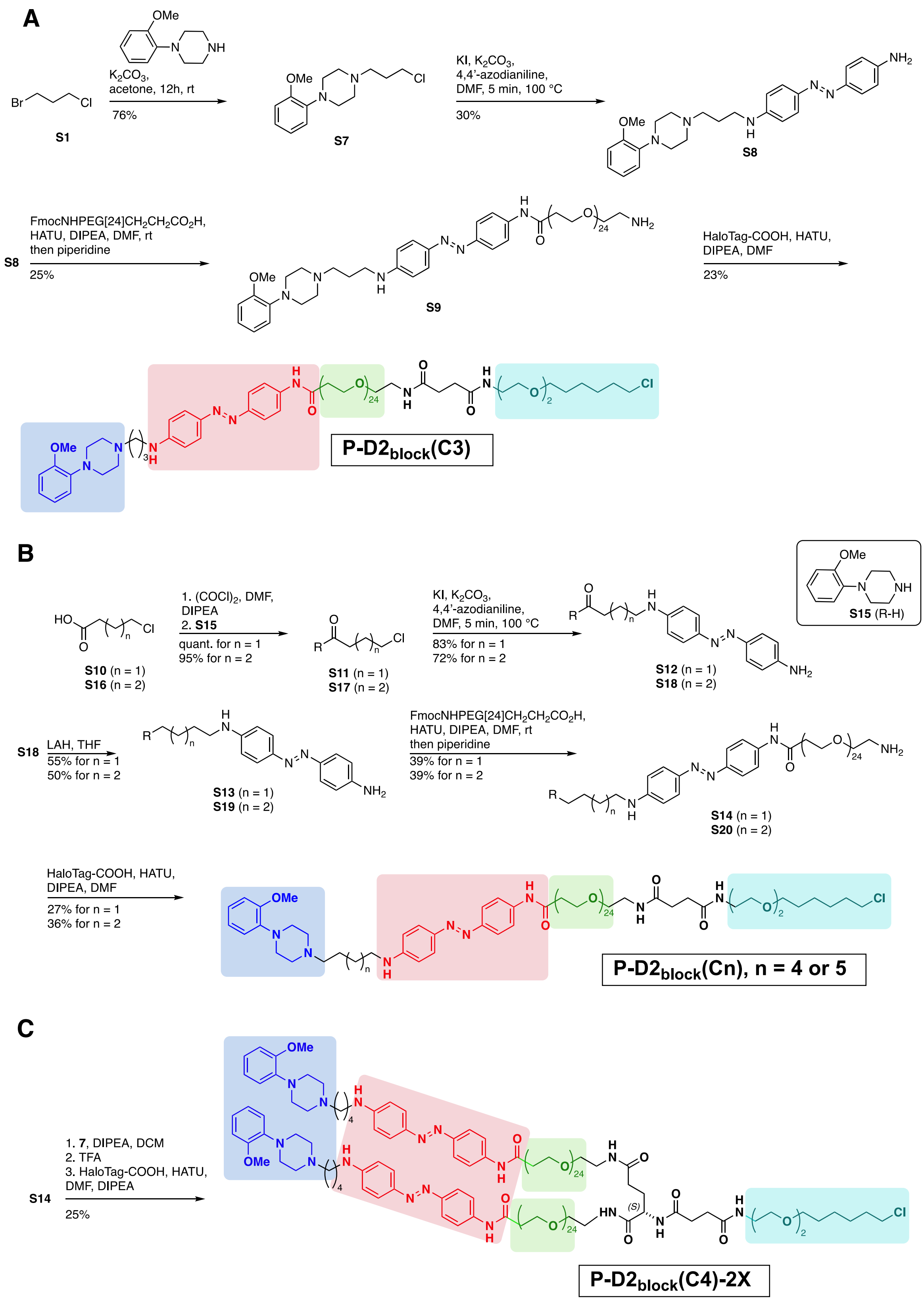

Figure S9. Synthesis of P-D2blocks. Preparation of **P-D2$_{\text{block}}$(C3)** (**A**), **P-D2$_{\text{block}}$(C4)** and **P-D2$_{\text{block}}$(C5)** (**B**), and **P-D2$_{\text{block}}$(C4)-2X** (**C**).

1 Experimental Details

1.1 General Methods, Reagents, Instrumentation

1.1.1 Reagents and Solvents

All **reagents** and **solvents** were purchased from commercial sources (Acros Organics, Alfa Aesar, Cayman, Combi-Blocks, Oakwood, Sigma Aldrich, TCI, TRC, etc.) and were used without further purification. Monodisperse PEG-linkers were purchased from PurePEG. Solvents were obtained from Fisher Scientific.

1.1.2 TLC, NMR, HRMS, FTIR, LCMS, HPLC

Reactions were monitored by thin layer chromatography (**TLC**) on glass plates precoated with silica gel (0.25 mm, 60 Å pore size, Merck). The plates were visualized by exposure to UV light (254 nm). Flash silica gel chromatography (**FCC**) was performed on a CombiFlash EZ PrepTM using silica gel (SiO_2, particle size 40-63 µm) purchased from SiliCycle. **NMR** spectra were measured on a Bruker AV-III HD 400 MHz (equipped with a CryoProbeTM) (operating at 400 MHz for ^{1}H and 100 MHz for ^{13}C) or on a Bruker AVIII-600 High Performance Digital NMR Spectrometer with a CPTCI-cryoprobe head (600 MHz for ^{1}H and 150 MHz for ^{13}C). Multiplicities in the following experimental procedures are abbreviated as follows: s = singlet, d = doublet, t = triplet, q = quartet, m = multiplet. ^{1}H chemical shifts are expressed in parts per million (ppm, δ scale). The residual protium in the deuterated solvent was used as internal reference (MeOD: δ = 7.26 or $CDCl_3$: δ = 7.26). ^{13}C chemical shifts are expressed in ppm (δ scale) and are referenced to the carbon resonance of the NMR solvent (MeOD: δ = 49.00 or $CDCl_3$: δ = 77.16). Structural analysis was conducted with ^{1}H- and ^{13}C-NMR spectra using additional 2D spectra (COSY, HMBC, HSQC). High-Resolution Mass Spectra (**HRMS**) were recorded on an Agilent 6224 Accurate-Mass TOF/LC/MS using an electrospray ionization source (ESI). **FTIR** were recorded on a ThermoScientific Nicolet-6700 Fourier Transform Infrared Spectroscopy system. **LCMS** analysis was performed on an LCMS 1260 Infinity II Agilent Technologies system (Windows 10, OpenLabs CDS Chemstation Software, 6120 Quadrupole LC/MS G7111B quaternary pump, G7129A Infinity II vialsampler, G7117C 1260 diode array detector) with an LC Kinetex column 2.6 µm C18 (50 x 3 mm). Runs were performed at a flow-rate of 1 mL/min with a run-time of 5 min, and a solvent gradient of 0-100% MeCN in water, containing 0.1% formic acid. Preparative **HPLC** was performed on a 1260 Infinity Agilent Technologies system (Windows 10, OpenLabs CDS Chemstation Software, two G1361A pumps, G2260A autosampler (2400 µ L max. injection volume), G1170A column switching valve, G7115A diode array detector, G1364B fraction

collector, using a semipreparative column (Phenomenex, Gemini 5 um C18 110 Å, 15- x 10 mm, product #00F-4435-N0) or a preparative column (Phenomenex, Gemini 5 um C18 110 Å, product #00F-4435-U0-AX). Runs were performed at a flowrate of 9 or 80 mL/min (if not specified otherwise), using solvent mixtures of MeCN in H_2O, containing 0.1% formic acid.

1.1.3 UV-Vis and Photophysical characterization

UV-Vis spectroscopy was performed on a Varian Cary 60 UV-Visible Spectrometer equipped with an Agilent Technologies PCB 1500 Water Peltier system for temperature control. Samples were measured using disposable Spectrometer/Photometer Ultra-Micro Cuvettes from BrandTech (10 mm light path, 1 mL sample) and irradiation was performed with a Cairn Research Optoscan Monochromator with Optosource High Intensity Arc Lamp equipped with a 75 W UXL-S50A lamp from USHIO Inc. Japan, set to 15 nm full width at half maximum. The Monochromator was controlled using a MATLAB program written by Christopher Arp. UV-Vis data were analyzed and plotted using GRAPHPAD Prism. Irradiation was achieved from the top of the cuvette through a fiber-optic cable. Reversible switching was performed by diluting samples to 20 µM and irradiating with 415/600 nm for 90 seconds each. 600 nm was chosen as a surrogate for "dark", where there is no absorption by the compound. Absorbance (reported as Abs, in arbitrary units) was measured at 420 nm over time. Thermal Relaxation was measured after 1 min irradiation with 415 nm at 10 or 20 µM, and the subsequent absorption increase was detected at 420 nm. The relaxation half-live of the *cis*-isomer was determined by curve fitting, using exponential one-phase decay in GRAPHPAD Prism. **Photophysical characterization by NMR** was performed in a Wilmad NMR tube (5 mm) equipped with a glass adapter for an optical fiber (single branch polymer fiber optical cord purchased from Prizmatix) connected to a high-power LED (415 nm). Spectra were gathered using the 600 MHz instrument. Spectrum analysis was conducted with the software MestReNova.

1.1.4 Molecular Biology and Heterologous Expression

All constructs were cloned into mammalian expression vectors and are available from the authors upon reasonable request.

For the receptor mediated-GIRK activation assay, HEK293T cells were seeded onto 18 mm coverslips and transiently transfected overnight with Lipofectamine 2000 and the following constructs: a receptor, the HaloTag membrane-anchor (M; 0.7 µg), GIRK1(F137S) (0.7 µg), and

tdTomato (0.2 µg). The following receptors were tested: D2R (0.2 µg), D3R (0.2 µg), D4R (0.2 µg), D1R (0.525 µg), D5R (0.35 µg), M1R (0.7 µg), M4R (0.7 µg), mGluR1 (0.7 µg), CB1R (0.7 µg), and $GABA_BR$ (0.7 µg of each subunit, B1 and B2). Some of these receptors do not couple to GIRK channels in HEK293T cells unless co-expressed with specific Gα subunits. D1R and D5R were co-transfected with $G\alpha_{is13}$ (0.35 µg), D3R with $G\alpha_{oA}$ (0.7 µg), and M1R or mGluR1 with $G\alpha_{iq5}$. Transfected cells were used for electrophysiology or flow cytometry experiments.

1.1.5 Confocal Imaging and Flow Cytometric Analysis of Cultured Cells

Untransfected or HaloTag membrane-anchor (M)-expressing HEK293T cells incubated with P-$D2_{ago}$-2X and were then labeled with 100 nM HaloTag-Alexa488 (Promega) for 10 minutes in the dark at 37°C and 5% CO_2 in a standard extracellular solution. Cells were then analyzed by flow cytometry (BD LSR II).

1.1.6 Electrophysiology

HEK293T cells were sparsely seeded and maintained in DMEM (Invitrogen) with 10% fetal bovine serum on poly-L-lysine-coated coverslips at 37°C and 5% CO_2. HEK293T cells were voltage clamped in whole-cell configuration 16-48 hours after transfection. For GIRK experiments, the extracellular solution contained 120 mM KCl, 25 mM NaCl, 10 mM HEPES, 2 mM CaCl2, and 1 mM MgCl2, pH 7.4. Glass pipettes with a resistance of 3-7 MΩ were filled with intracellular solution containing 120 mM Gluconic acid δ-lactone, 15 mM CsCl, 10 mM BAPTA, 10 mM HEPES, 1 mM $CaCl_2$, 3 $MgCl_2$, 3 mM MgATP, pH 7.2. Cells were voltage clamped to -80 mV using an Axopatch 200A (Molecular Devices) amplifier.

To conjugate P-D2 variants to the M, cells were incubated with varying concentrations for up to 60 minutes in the dark at 37°C in standard extracellular buffers. For all experiments, compounds were applied using a gravity-driven perfusion system and illumination was applied to the entire field of view using a DG4 (Sutter) through a 20x objective (1.6 mW/mm^2 at 440 nm). pClamp software was used for both data acquisition and control of illumination.

The selection criteria for electrophysiological experiments are that a cell (i) expresses the fluorescent protein transfection marker, and (ii) responds to agonist, indicating the presence of either receptor and GIRK. Cells were not excluded unless the recording was of poor quality (e.g., unstable baseline).

1.2 Chemistry

1.2.1 3: Indane Chloride

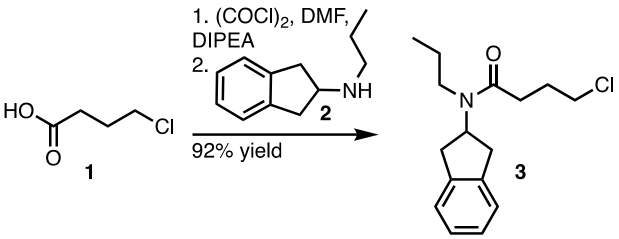

In a 25 mL rbf, 4-chlorobutyric acid (**1**) (0.201 mL, 2.013 mmol, 1.20 equiv.) and DMF (1 drop) were dissolved in THF (10 mL). The mixture was cooled in an ice-water bath, and then oxalyl chloride (0.201 mL, 2.348 mmol, 1.40 equiv.) was added dropwise under evolution of bubbles. The mixture was warmed to room temperature and stirred for another 30 min. The solvent was removed under reduced pressure and the yellow oily residue taken up in THF (1 mL). In a separate 25 mL rbf, **2** (300 mg, 1.68 mmol, 1.00 equiv.), DIPEA (0.64 mL, 3.7 mmol, 2.2 equiv.), and DMAP (10.2 mg, 0.084 mmol, 0.05 equiv.) were dissolved in THF (10 mL) and cooled in an ice-water bath. The acyl chloride solution was added dropwise under evolution of fumes. After 30 seconds, a white precipitate occurred. The reaction was stirred at room temperature for 10 min, then the solvent removed under reduced pressure. The crude white solid was subjected to FCC (24 g silica, 0 -> 60% EtOAc in hexanes), to yield the product **3** as light-yellow oil in 92% yield (430 mg, 1.54 mmol).

R_f = 0.74 (5% MeOH in DCM; UV detection).

HRMS (ESI): calc. for $C_{16}H_{22}ClNONa^+$ $[M+Na]^+$: 302.1282; found 302.1299.

LCMS (5-100% MeCN in H2O with 0.1% formic acid over 5 min) t_R = 4.265 min, 254 nm detection.

LRMS (ESI): calc. for $C_{16}H_{23}ClNO^+$ $[M+H]^+$: 280.2; found 280.2.

^{1}H NMR (400 MHz, $CDCl_3$) δ 7.17 (m, 4H), 5.13 (t, *J* = 8.2 Hz, 0.5H), 4.77 (t, *J* = 8.3 Hz, 0.5H), 3.66 (t, *J* = 6.1 Hz, 2H), 3.25 – 3.01 (m, 6H), 2.61 (t, *J* = 7.0 Hz, 1H), 2.54 (t, *J* = 7.0 Hz, 1H), 2.16 (p, *J* = 6.6 Hz, 2H), 1.66 – 1.54 (m, 2H), 0.85 (q, *J* = 7.7 Hz, 3H). *(Reported as mix of rotamers)*

^{13}C NMR (101 MHz, $CDCl_3$) δ 171.80, 171.26, 141.17, 140.27, 126.97, 126.55, 124.52, 124.39, 57.72, 55.69, 47.28, 45.05, 45.00, 44.44, 37.00, 36.62, 30.30 (*CX2*), 28.03, 24.06, 22.32, 11.54, 11.29.

(Reported as mix of rotamers)

IR (neat) 2962 (w), 2832 (w), 1635 (s), 1459 (m), 1420 (m), 1224 (w), 1119 (w), 744 (s) cm^{-1}.

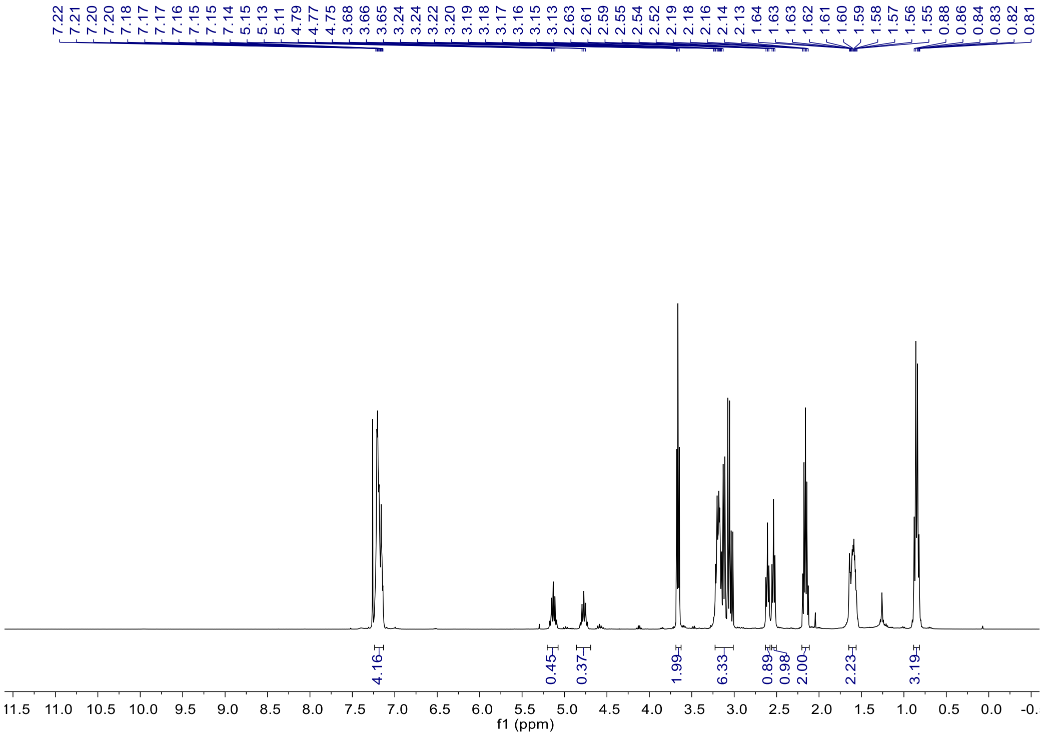
7.22
7.21
7.20
7.20
7.18
7.17
7.17
7.16
7.15
7.15
7.14
5.15
5.13
5.11
4.79
4.77
4.75
3.68
3.66
3.65
3.24
3.24
3.22
3.20
3.19
3.18
3.17
3.16
3.15
3.13
2.63
2.61
2.59
2.55
2.54
2.52
2.19
2.18
2.16
2.14
2.13
1.64
1.63
1.63
1.62
1.61
1.60
1.59
1.58
1.57
1.56
1.55
0.88
0.86
0.84
0.83
0.82
0.81
4.16
0.45
0.37
1.99
6.33
0.89
0.98
2.00
2.23
3.19
f1 (ppm)

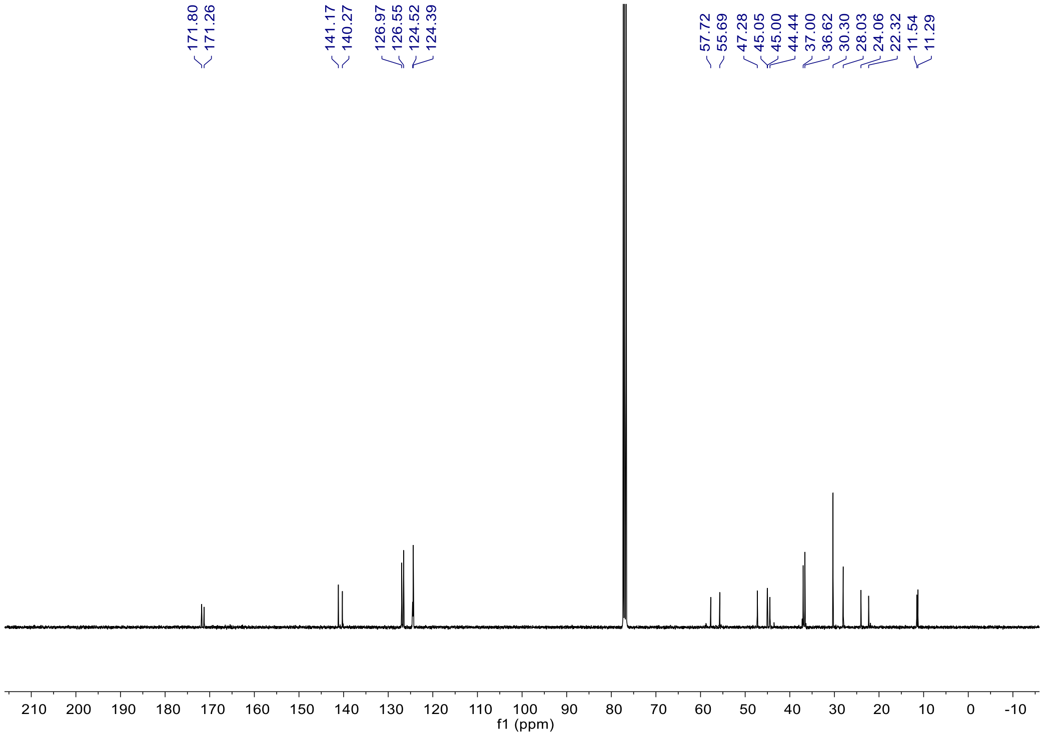
171.80
171.26
141.17
140.27
126.97
126.55
124.52
124.39
57.72
55.69
47.28
45.05
45.00
44.44
37.00
36.62
30.30
28.03
24.06
22.32
11.54
11.29
f1 (ppm)

1.2.2 4: Indane Azo

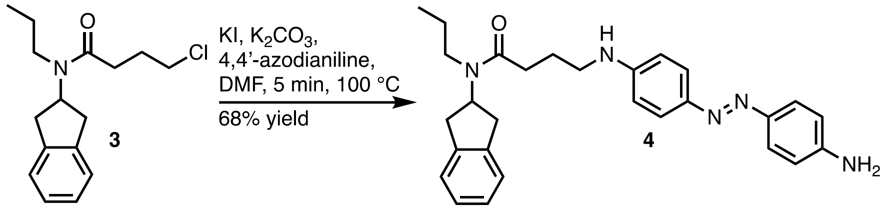

In a 20 mL scintillation vial, **3** (102 mg, 0.365 mmol, 1.00 equiv.), KI (181 mg, 1.09 mmol, 3.00 equiv.) and 4,4'-azodianiline (81.5 mg, 0.365 mmol, 1.00 equiv.) were dissolved in DMF (2.0 mL), and the orange reaction mixture heated to 60 °C. After 2h, the dark green reaction was judged complete by LCMS analysis. The solvent was removed under reduced pressure at 50 °C. The residue was taken up in DCM and washed with LiCl (10 %, aq.). The organic phase was dried over Na_2SO_4, filtered, and concentrated under reduced pressure. The red residue was subjected to FCC (24g silica, 0 -> 6% MeOH in DCM), to yield the product **4** as dark orange oil in 68% yield (113 mg, 0.247 mmol).

R_f = 0.33 (5% MeOH in DCM; UV detection + green spot).

HRMS (ESI): calc. for $C_{28}H_{33}N_5ONa^+$ [M+Na]$^+$: 478.2577; found 478.2585.

LCMS (5-100% MeCN in H_2O with 0.1% formic acid over 5 min) t_R = 4.241 min, 360 nm detection.

LRMS (ESI): calc. for $C_{28}H_{34}N_5O^+$ [M+H]$^+$: 456.3; found 456.3

^{1}H NMR (400 MHz, $CDCl_3$) δ 7.8 – 7.7 (m, 4H), 7.2 (d, *J* = 6.5 Hz, 4H), 6.7 (d, *J* = 8.4 Hz, 2H), 6.7 – 6.6 (m, 2H), 5.1 (t, *J* = 8.2 Hz, 0.5H), 4.8 – 4.7 (m, 0.5H), 3.3 (t, *J* = 6.5 Hz, 2H), 3.2 – 3.2 (m, 1H), 3.1 (td, *J* = 8.0, 3.0 Hz, 2H), 3.1 – 3.0 (m, 3H), 2.6 (t, *J* = 6.7 Hz, 1H), 2.5 (t, *J* = 6.7 Hz, 1H), 2.1 (td, *J* = 6.8, 3.8 Hz, 2H), 1.6 – 1.5 (m, 2H), 0.8 (dt, *J* = 14.7, 7.4 Hz, 3H).

(Reported as mix of rotamers)

^{13}C NMR (101 MHz, $CDCl_3$) δ 172.8, 172.3, 150.2, 148.2, 145.8, 144.6, 141.2, 140.2, 127.0, 126.6, 124.6, 124.5, 124.4, 124.1, 114.8, 112.2, 57.9, 56.0, 47.5, 44.6, 43.8, 43.7, 37.0, 36.6, 31.6, 31.5, 24.4, 24.4, 24.0, 22.3, 11.6, 11.3.

(Reported as mix of rotamers)

IR (neat) 3341 (b), 2961 (w), 1622 (m), 1594 (s), 1516 (w), 1425 (w), 1295 (w), 1147 (m), 835 (w), 746 (w) cm^{-1}.

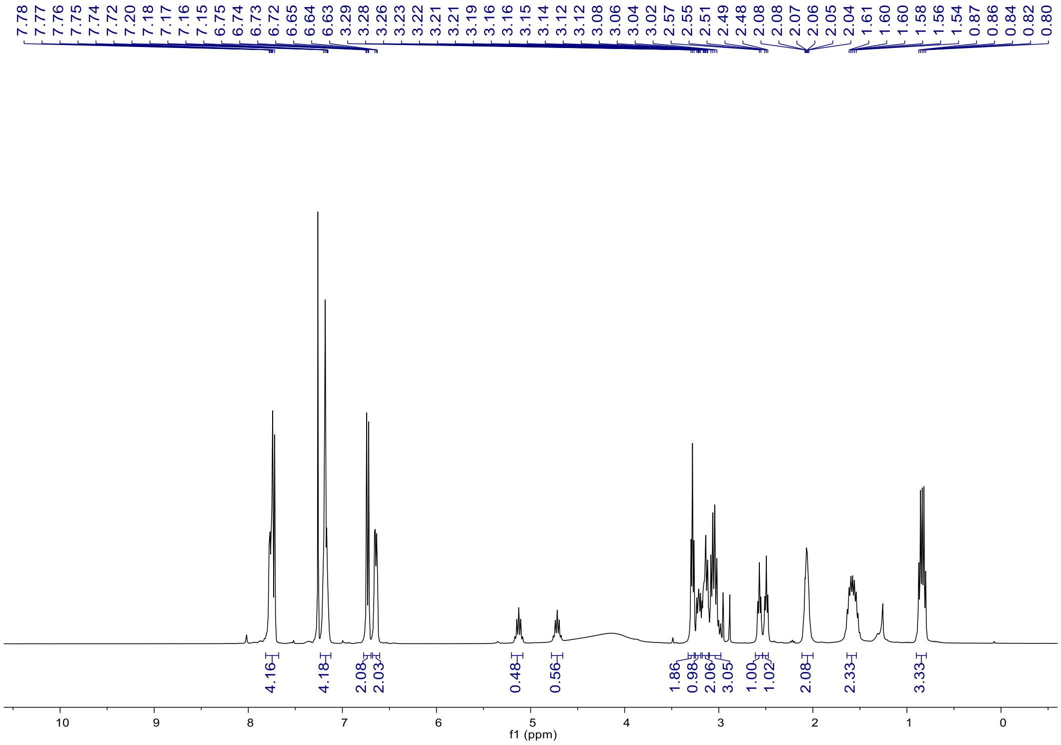
7.78 7.77 7.76 7.75 7.74 7.72 7.20 7.18 7.17 7.16 7.15 6.75 6.74 6.73 6.72 6.65 6.64 6.63 3.29 3.28 3.26 3.23 3.22 3.21 3.21 3.19 3.16 3.16 3.15 3.14 3.12 3.12 3.08 3.06 3.04 3.02 2.57 2.55 2.51 2.49 2.48 2.08 2.08 2.07 2.06 2.05 2.04 1.61 1.60 1.60 1.58 1.56 1.54 0.87 0.86 0.84 0.82 0.80
4.16 4.18 2.08 2.03 0.48 0.56 1.86 0.98 2.06 3.05 1.00 1.02 2.08 2.33 3.33
10 9 8 7 6 5 4 3 2 1 0
f1 (ppm)

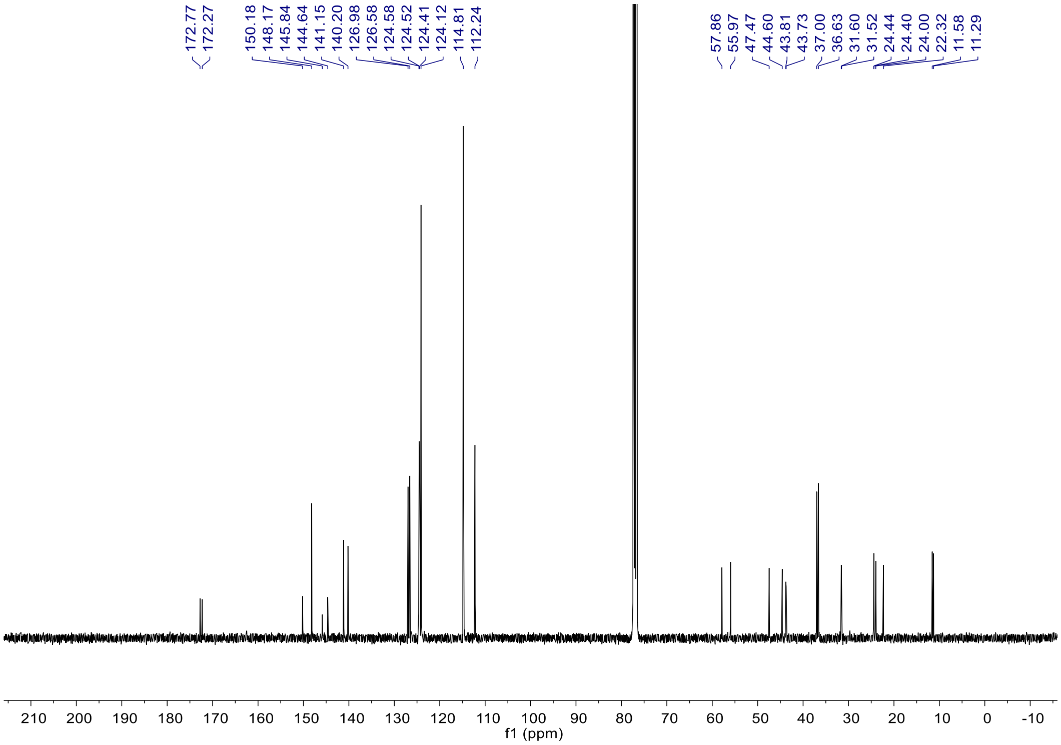
172.77 172.27 150.18 148.17 145.84 144.64 141.15 140.20 126.98 126.58 124.52 124.41 124.12 114.81 112.24 57.86 55.97 47.47 44.60 43.81 43.73 37.00 36.63 31.60 31.52 24.44 24.40 24.00 22.32 11.58 11.29
210 200 190 180 170 160 150 140 130 120 110 100 90 80 70 60 50 40 30 20 10 0 -10
f1 (ppm)

1.2.3 5: Aminoindane Azo

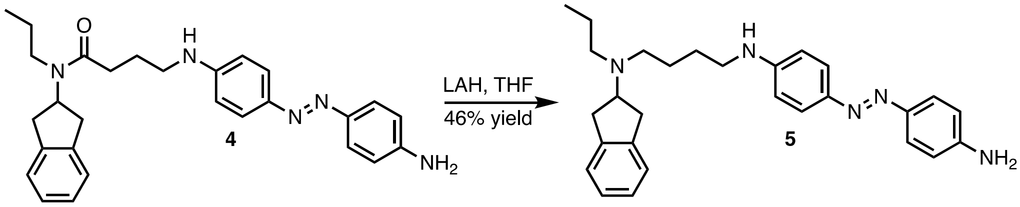

In a 5 mL rbf, LAH (1M in THF, 0.632 mL, 0.632 mmol, 8.00 equiv.) was cooled in an ice-water bath. **4** (36.0 mg, 0.079 mmol, 1.00 equiv., as solution in 0.4 mL THF) was added dropwise. The dark red solution was warmed to room temperature and stirred for another 30 min. Then, the solution was cooled in an ice-water bath, and NaOH (5 mL, 2M) was added slowly under evolution of bubbles. Then, DCM was added, and the phases separated. The organic phase was dried over Na_2SO_4, filtered, and concentrated under reduced pressure. The red oil was subjected to FCC (4g silica, 0 -> 5% MeOH in DCM) to yield the desired product **5** in 46% yield (16 mg, 0.036 mmol).

R*f* = 0.14 (5% MeOH in DCM; UV detection + brown spot).

HRMS (ESI): calc. for $C_{28}H_{36}N_5^+$ [M+H] $^+$: 442.2965; found 442.2958.

LCMS (5-100% MeCN in H_2O with 0.1% formic acid over 5 min) t_R = 3.129 min, 360 nm detection.

LRMS (ESI): calc. for $C_{28}H_{36}N_5^+$ [M+H] $^+$: 442.3; found 442.3.

^{1}H NMR (400 MHz, $CDCl_3$) δ 7.80 – 7.68 (m, 4H), 7.20 – 7.10 (m, 4H), 6.75 – 6.71 (m, 2H), 6.65 – 6.62 (m, 2H), 4.34 (br s, 1H, N*H*), 3.91 (s, 2H, N*H*), 3.71 (t, *J* = 8.2 Hz, 1H), 3.21 (t, *J* = 6.5 Hz, 2H), 3.10 – 2.88 (m, 4H), 2.66 – 2.49 (m, 4H), 1.68 (m, 4H), 1.54 (q, *J* = 7.6 Hz, 2H), 0.90 (t, *J* = 7.3 Hz, 3H).

^{13}C NMR (101 MHz, $CDCl_3$) δ 150.20, 148.15, 145.97, 144.68, 141.72, 126.35, 124.50, 124.45, 124.12, 114.80, 112.15, 62.99, 53.32, 51.05, 43.65, 36.37, 27.51, 24.95, 20.05, 11.95.

IR (neat) 3381 (b), 2930 (w), 2867 (w), 1595 (s), 1506 (m), 1292 (w), 1241 (m), 1147 (m), 1024 (w), 832 (w), 743 (w) cm^{-1}.

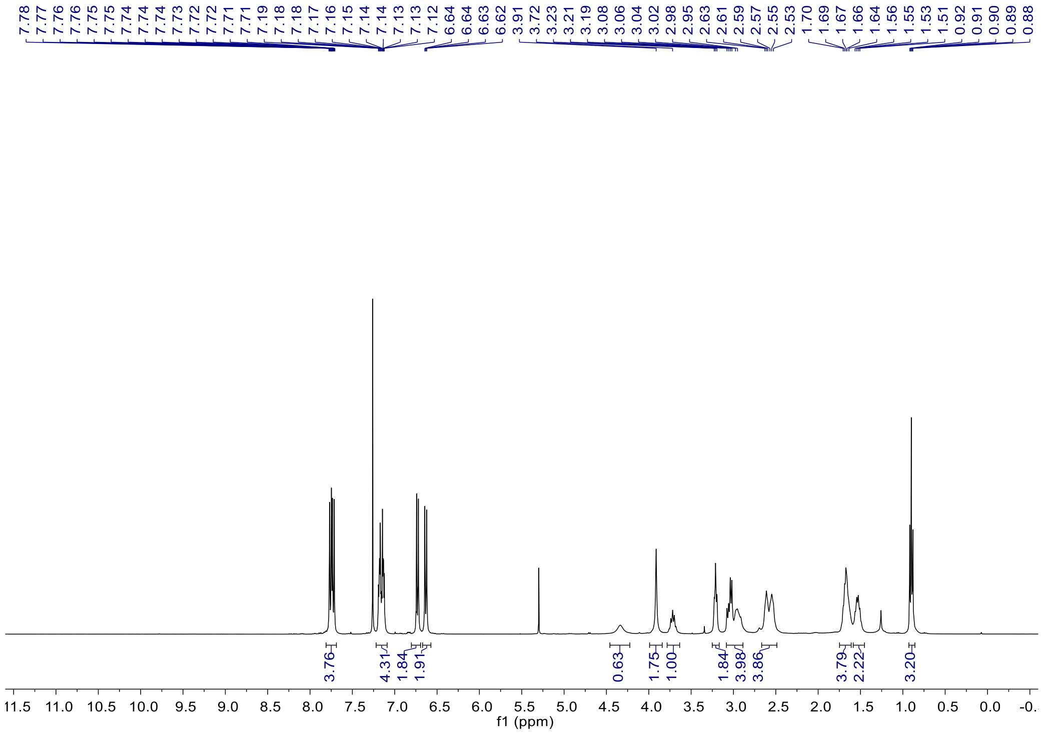
7.78 7.77 7.76 7.76 7.75 7.75 7.74 7.74 7.74 7.73 7.72 7.72 7.71 7.71 7.19 7.18 7.18 7.17 7.16 7.15 7.14 7.14 7.13 7.13 7.13 7.12 6.64 6.64 6.63 6.62 3.91 3.72 3.23 3.21 3.19 3.08 3.06 3.04 3.02 2.98 2.95 2.63 2.61 2.59 2.57 2.55 2.53 1.70 1.69 1.67 1.66 1.64 1.56 1.55 1.53 1.51 0.92 0.91 0.90 0.89 0.88
3.76 4.31 1.84 1.91 0.63 1.75 1.00 1.84 3.98 3.86 3.79 2.22 3.20
f1 (ppm)

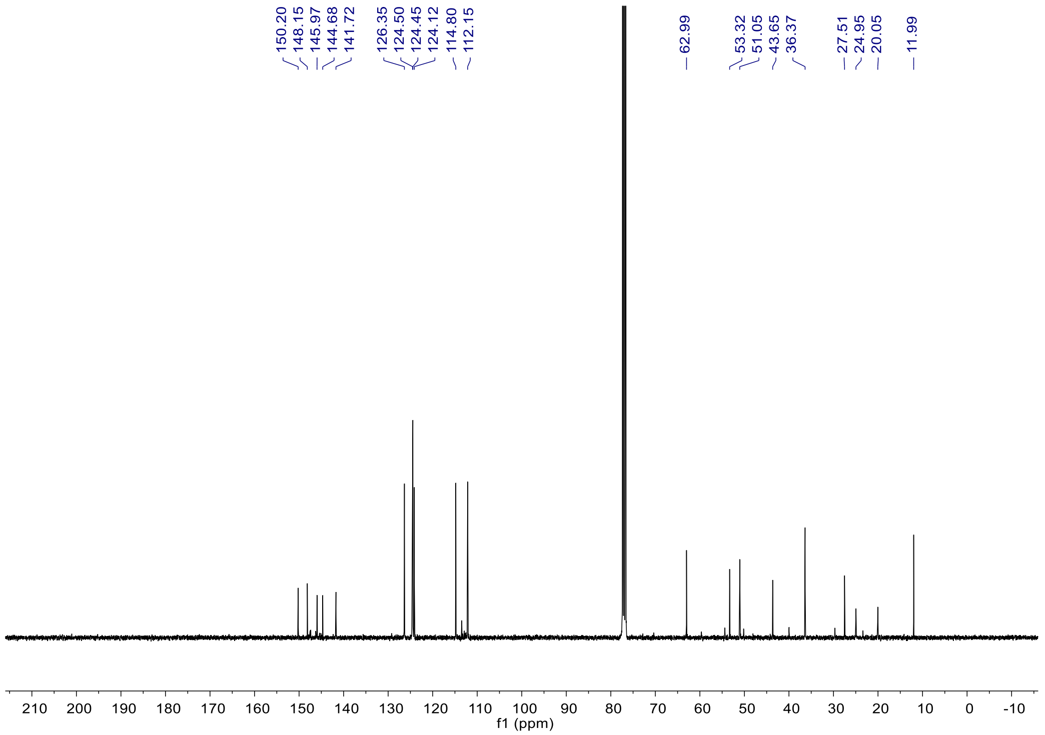
150.20 148.15 145.97 144.68 141.72 126.35 124.50 124.45 124.12 114.80 112.15 62.99 53.32 51.05 43.65 36.37 27.51 24.95 20.05 11.99
f1 (ppm)

1.2.4 6: Aminoindane Azo PEG

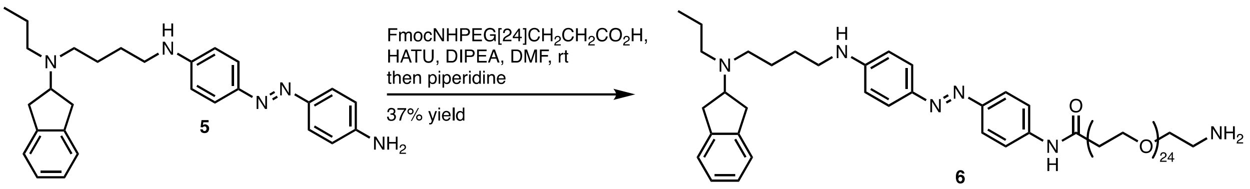

In a 20 mL vial, **5** (65 mg, 0.147 mmol, 1.00 equiv.) and FmocHNPEG[24]CH_2CH_2COOH (201 mg, 0.147 mmol, 1.00 equiv.) were dissolved in DMF (2 mL), and DIPEA (0.13 mL, 0.736 mmol, 5.00 equiv.) was added. Then, HATU (58.9 mg, 0.147 mmol, 1.00 equiv.) was added in one portion. After 10h, LCMS showed full conversion to the desired product. Piperidine (0.3 mL) was added, and LCMS confirmed complete deprotection, the crude reaction solution was diluted with MeCN and treated with AcOH (200 uL), and subjected to RP-HPLC purification (15-42% MeCN in water containing 0.1% FA, 80 mL/min, 8 min runtime, prep column, 360 nm detection, t_R = 4.598 min). After evaporation of the solvent under reduced pressure, the desired product **6** was obtained in 37% yield as orange oil (83.8 mg, 0.054 mmol).

Fmoc Intermediate:

LCMS (5-100% MeCN in H_2O with 0.1% formic acid over 5 min) t_R = 3.820 min, 360 nm detection.

LRMS (ESI): calc. for $C_{94}H_{148}N_6O_{27}^{2+}$ $[M+2H]^{2+}$: 896.5; found 896.7.

Product:

HRMS (ESI): calc. for $C_{79}H_{136}N_6O_{25}Na_2^{2+}$ $[M+2Na]^{2+}$: 818.9619; found 818.9575.

LCMS (5-100% MeCN in H_2O with 0.1% formic acid over 5 min) t_R = 3.093 min, 360 nm detection.

LRMS (ESI): calc. for $C_{79}H_{138}N_6O_{25}^{2+}$ $[M+2H]^{2+}$: 785.5; found 785.6.

^{1}H NMR (400 MHz, $CDCl_3$) δ 9.04 (s, 1H), 7.79 (m, 4H), 7.75 – 7.70 (m, 2H), 7.14 (m, 4H), 6.64 (d, *J* = 8.8 Hz, 2H), 4.63 (br s, 1H), 3.88 – 3.81 (m, 4H), 3.65 – 3.60 (m, 96H), 3.22 (m, 2H), 3.13 (t, *J* = 5.0 Hz, 3H), 3.05 (dd, *J* = 13.0, 8.0 Hz, 3H), 2.69 (m, 4H), 2.65 – 2.59 (m, 2H), 1.71 (m, 4H), 1.58 (m, 4H), 0.91 (t, *J* = 7.4 Hz, 3H).

^{13}C NMR (101 MHz, $CDCl_3$) δ 170.08, 150.79, 149.15, 144.51, 141.10, 139.85, 126.59, 125.00, 124.44, 122.97, 119.90, 112.08, 70.53, 70.51, 70.48, 70.46, 70.44, 70.42, 70.39, 70.32, 70.29, 70.14, 70.10, 70.07, 70.05, 69.96, 69.90, 69.75, 69.73, 67.57, 67.41, 67.11, 63.04, 53.09, 50.94, 46.68, 43.33, 42.55, 40.62, 37.97, 36.01, 33.57, 29.67, 27.19, 26.42, 25.52, 24.52, 11.85.

IR (neat) 3430 (b), 2865 (m), 1595 (s), 1534 (m), 1459 (w), 1298 (w), 1249 (w), 1095 (s), 947 (w), 843 (m), 749 (w) cm^{-1}.

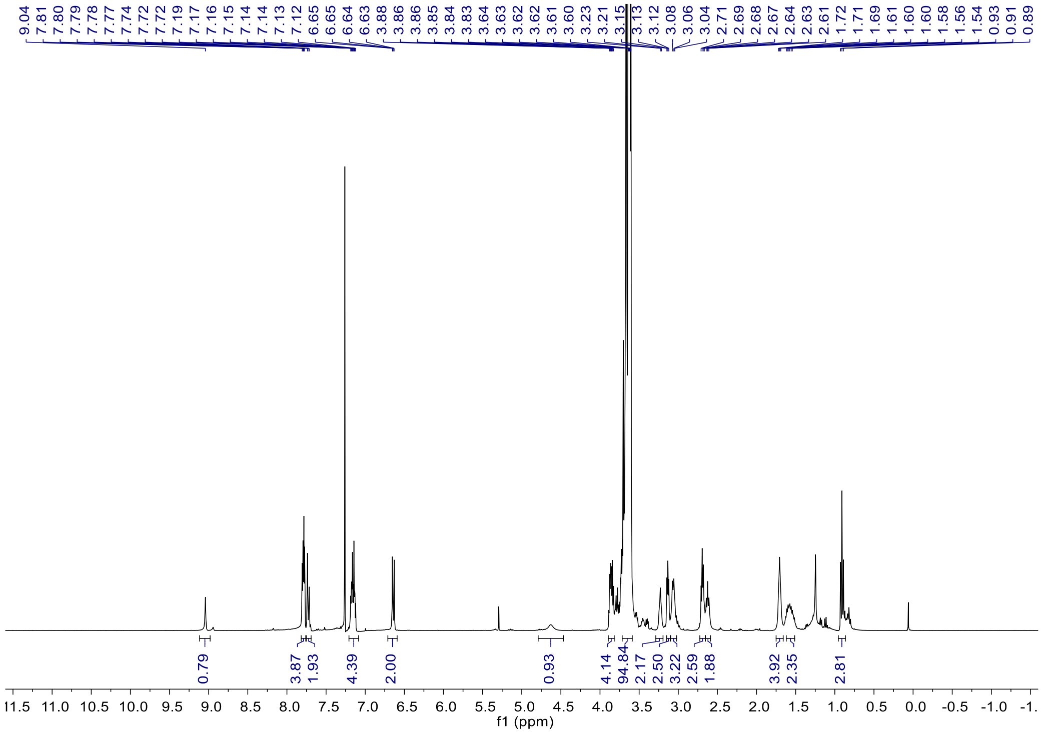

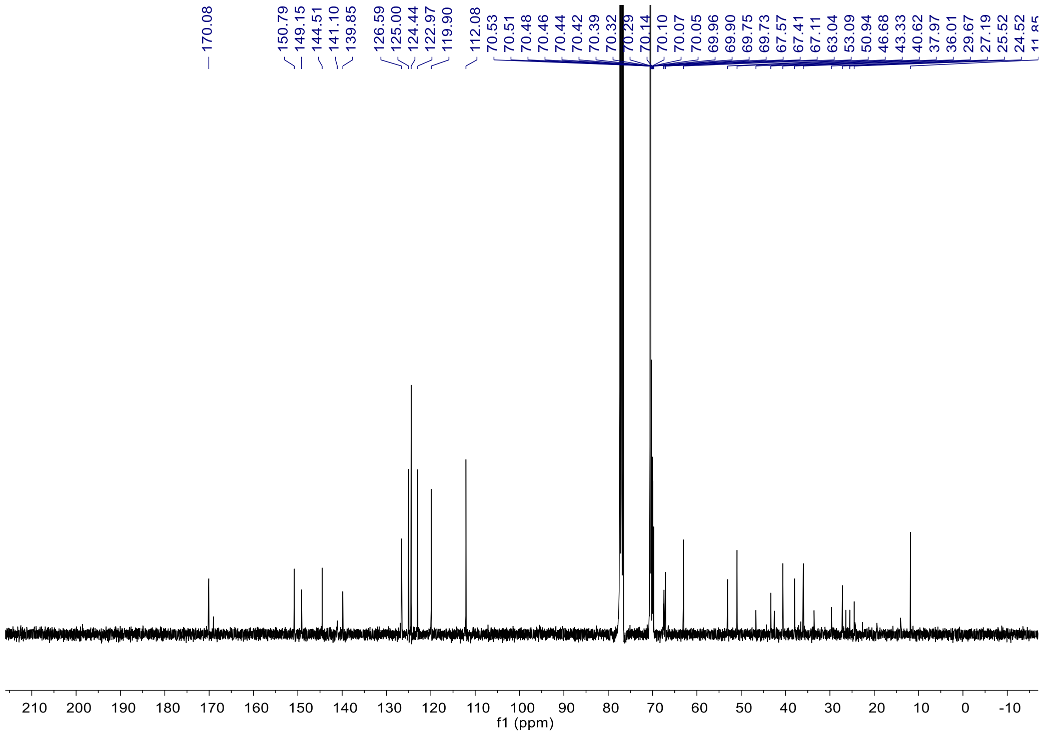

1.2.5 P-D2$_{ago}$

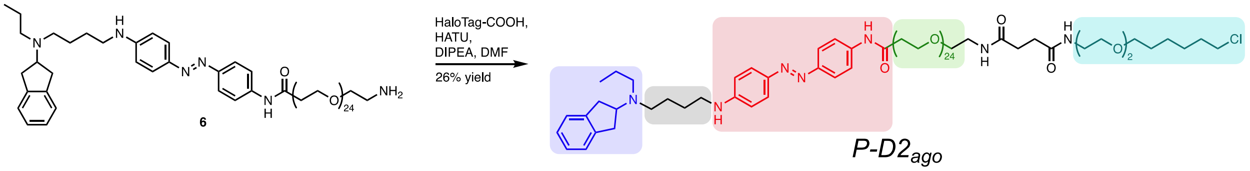

In a 4 mL vial, **6** (19.0 mg, 0.012 mmol, 1.00 equiv.) and HaloTag-COOH (4.3 mg, 0.012 mmol, 1.00 equiv.) were dissolved in DMF (0.1 mL), and DIPEA (9 uL, 0.049 mmol, 4.0 equiv.) was added. Then, HATU (4.8 mg, 0.012 mmol, 1.00 equiv.) was added in one portion. After 10h, LCMS showed full conversion to the desired product. The crude reaction solution was diluted with MeCN and treated with AcOH (20 uL), and subjected to RP-HPLC purification (10-50% MeCN in water containing 0.1% FA, 9 mL/min, 11 min runtime, prep. column, 360 nm detection, t_R = 8.140 min). After evaporation of the solvent under reduced pressure, the desired product **P-D2$_{ago}$** was obtained in 26% yield as orange oil (6.0 mg, 3.2 umol).

HRMS (ESI): calc. for $C_{93}H_{160}ClN_7Na_2O_{29}^{2+}$ [M+2Na]$^{2+}$: 960.5383; found 960.5306

LCMS (5-100% MeCN in H_2O with 0.1% formic acid over 5 min) t_R = 3.616min, 360 nm detection.

LRMS (ESI): calc. for $C_{93}H_{163}ClN_7O_{29}^{3+}$ [M+3H]$^{3+}$: 626.04; found 626.0.

^{1}H NMR (400 MHz, MeOD) δ 7.82 – 7.73 (m, 6H), 7.21 – 7.17 (m, 2H), 7.15 – 7.10 (m, 2H), 6.77 – 6.68 (m, 2H), 3.86 (t, *J* = 5.9 Hz, 2H), 3.66 – 3.62 (m, 84H), 3.55 (t, *J* = 5.4 Hz, 4H), 3.50 (t, *J* = 6.6 Hz, 2H), 3.37 (t, *J* = 5.5 Hz, 4H), 3.28 – 3.23 (m, 2H), 3.14 (dd, *J* = 15.6, 7.7 Hz, 2H), 2.94 (dd, *J* = 15.5, 8.5 Hz, 2H), 2.79 – 2.73 (m, 2H), 2.69 (m, 4H), 2.50 (s, 4H), 1.82 – 1.74 (m, 2H), 1.72 – 1.68 (m, 4H), 1.60 (q, *J* = 7.0 Hz, 4H), 1.52 – 1.39 (m, 3H), 0.95 (t, *J* = 7.3 Hz, 3H).

IR (neat) 3328 (b), 2866 (m), 1668 (w), 1600 (s), 1537 (m), 1459 (w), 1348 (w), 1300 (w), 1250 (m), 1104 (s), 946 (w), 846 (m) cm^{-1}.

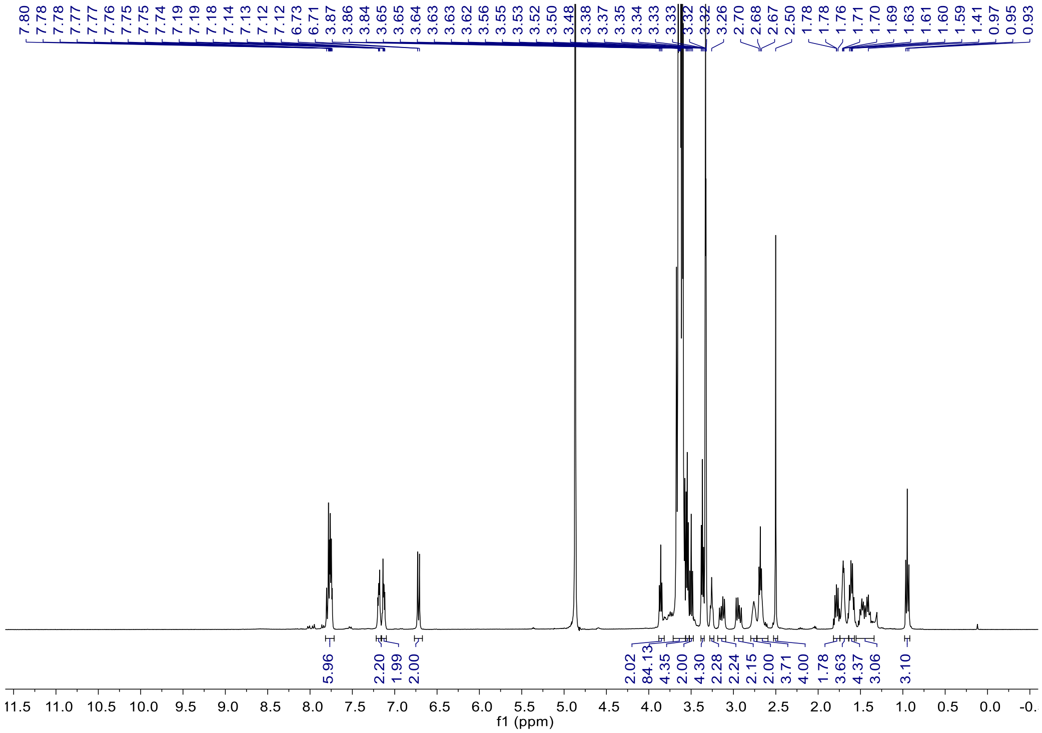
7.80 7.78 7.78 7.77 7.77 7.76 7.75 7.75 7.74 7.19 7.19 7.18 7.14 7.13 7.12 7.12 6.73 6.71 3.87 3.86 3.84 3.65 3.65 3.64 3.63 3.63 3.62 3.56 3.55 3.53 3.52 3.50 3.48 3.38 3.37 3.35 3.34 3.33 3.33 3.32 3.32 3.26 2.70 2.68 2.67 2.50 1.78 1.78 1.76 1.71 1.70 1.69 1.63 1.61 1.60 1.59 1.41 0.97 0.95 0.93
5.96 2.20 1.99 2.00 2.02 84.13 4.35 2.00 4.30 2.28 2.24 2.15 2.00 3.71 4.00 1.78 3.63 4.37 3.06 3.10
11.5 11.0 10.5 10.0 9.5 9.0 8.5 8.0 7.5 7.0 6.5 6.0 5.5 5.0 4.5 4.0 3.5 3.0 2.5 2.0 1.5 1.0 0.5 0.0 -0.
f1 (ppm)

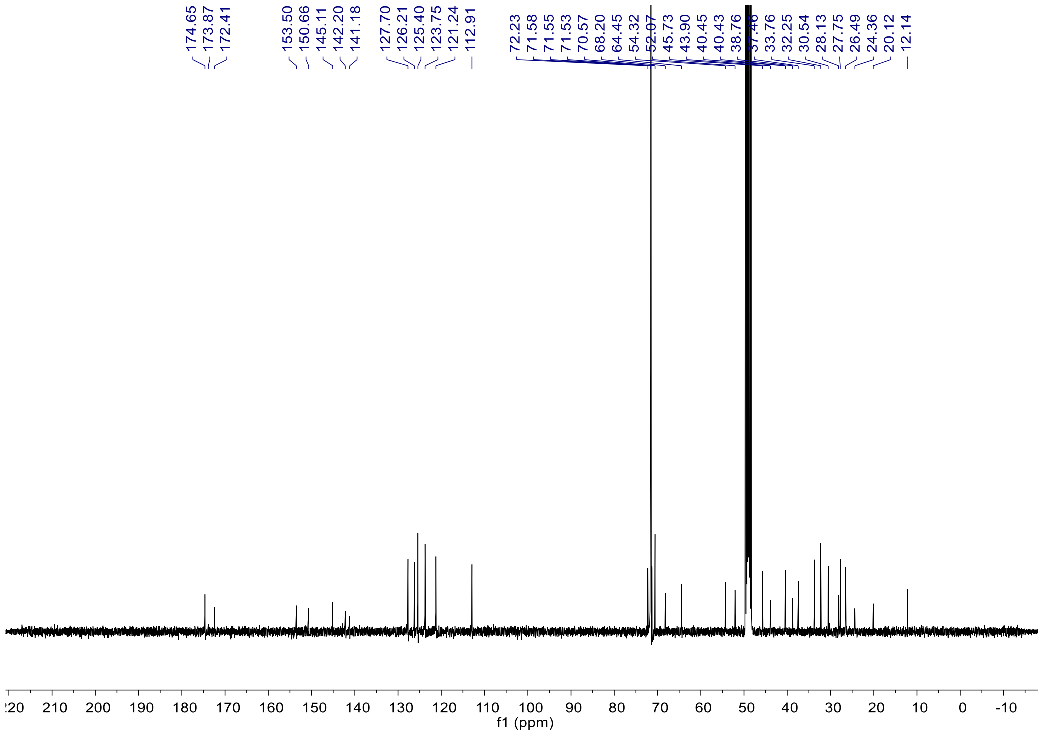
174.65 173.87 172.41 153.50 150.66 145.11 142.20 141.18 127.70 126.21 125.40 123.75 121.24 112.91 72.23 71.58 71.55 71.53 70.57 68.20 64.45 54.32 52.07 45.73 43.90 40.45 40.43 38.76 37.46 33.76 32.25 30.54 28.13 27.75 26.49 24.36 20.12 12.14
220 210 200 190 180 170 160 150 140 130 120 110 100 90 80 70 60 50 40 30 20 10 0 -10
f1 (ppm)

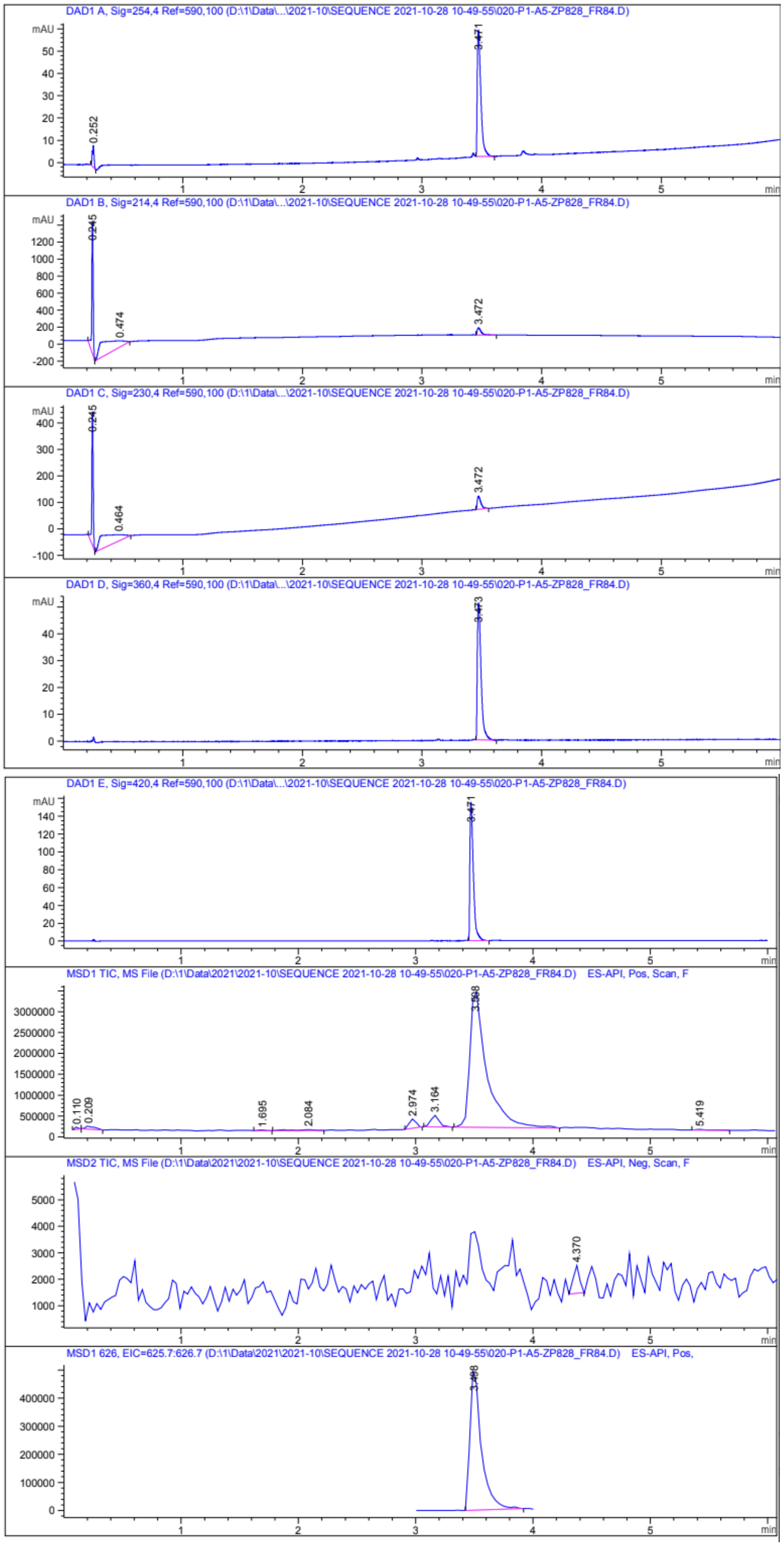
DAD1 A, Sig=254,4 Ref=590,100 (D:\1\Data\...\2021-10\SEQUENCE 2021-10-28 10-49-55\020-P1-A5-ZP828_FR84.D)
DAD1 B, Sig=214,4 Ref=590,100 (D:\1\Data\...\2021-10\SEQUENCE 2021-10-28 10-49-55\020-P1-A5-ZP828_FR84.D)
DAD1 C, Sig=230,4 Ref=590,100 (D:\1\Data\...\2021-10\SEQUENCE 2021-10-28 10-49-55\020-P1-A5-ZP828_FR84.D)
DAD1 D, Sig=360,4 Ref=590,100 (D:\1\Data\...\2021-10\SEQUENCE 2021-10-28 10-49-55\020-P1-A5-ZP828_FR84.D)
DAD1 E, Sig=420,4 Ref=590,100 (D:\1\Data\...\2021-10\SEQUENCE 2021-10-28 10-49-55\020-P1-A5-ZP828_FR84.D)
MSD1 TIC, MS File (D:\1\Data\2021\2021-10\SEQUENCE 2021-10-28 10-49-55\020-P1-A5-ZP828_FR84.D) ES-API, Pos, Scan, F
MSD2 TIC, MS File (D:\1\Data\2021\2021-10\SEQUENCE 2021-10-28 10-49-55\020-P1-A5-ZP828_FR84.D) ES-API, Neg, Scan, F
MSD1 626, EIC=625.7:626.7 (D:\1\Data\2021\2021-10\SEQUENCE 2021-10-28 10-49-55\020-P1-A5-ZP828_FR84.D) ES-API, Pos,
mAU
min

1.2.6 P-D2$_{ago}$-2X

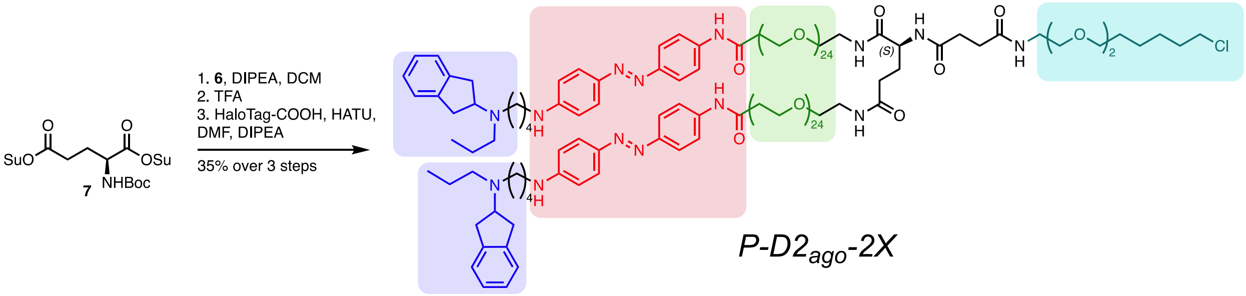

In a 20 mL vial, **6** (21.9 mg, 14.0 umol, 2.20 equiv.) and NHBoc-L-Glu-OSu (2.8 mg, 6.0 umol, 1.00 equiv.) were dissolved in DMF (100 uL), and DIPEA (5 uL, 26.0 umol, 4.00 equiv.) was added. After 10 min, LCMS showed full conversion to the desired dimer. The solvent was removed under reduced pressure, then TFA (1 mL) was added to the dark red oil, to form a magenta solution. After 5 min, TFA was removed under a gentle stream of nitrogen. This procedure was repeated twice until LCMS showed full conversion to the free amine. Then, HaloTag-COOH (2.3 mg, 7.0 umol, 1.1 equiv., as solution in 100 uL DMF) and DIPEA (20 uL, 130 umol, 20.0 equiv.) were added, forming a dark orange solution. HATU (2.5 mg, 6.0 umol, 1.0 equiv.) was added in one portion. After 12h, LCMS showed full conversion of the starting material was detected and the sample subjected to RP-HPLC purification (25-55% MeCN in water containing 0.1% FA, 9 mL/min, 11 min runtime, semiprep column, 360 nm detection, t_R = 8.968 min). After evaporation of the solvent under reduced pressure, the desired product **P-D2$_{ago}$-2X** was obtained in 35% yield as orange oil (8.0 mg, 0.0022 mmol).

<u>Intermediate NHBoc Dimer</u>

LCMS (5-100% MeCN in H_2O with 0.1% formic acid over 5 min) t_R = 3.723 min, 360 nm detection.

LRMS (ESI): calc. for $C_{168}H_{289}N_{13}O_{54}^{4+}$ $[M+4H]^{4+}$: 838.8; found 838.70.

<u>Intermediate NH2 Dimer</u>

LCMS (5-100% MeCN in H_2O with 0.1% formic acid over 5 min) t_R = 3.548 min, 420 nm detection.

LRMS (ESI): calc. for $C_{163}H_{282}N_{13}O_{52}^{5+}$ $[M+5H]^{5+}$: 651.2; found 651.0.

<u>Product</u>

LCMS (5-100% MeCN in H_2O with 0.1% formic acid over 5 min) t_R = 3.798 min, 360 nm detection.

LRMS (ESI): calc. for $C_{177}H_{307}ClN_{14}O_{56}^{6+}$ $[M+6H]^{6+}$: 593.68; found 593.6.

^{1}H NMR (400 MHz, MeOD) δ 9.93 (s, 1H, N*H*), 7.83 – 7.75 (m, 12H), 7.30 – 7.22 (m, 8H), 6.88 (s, 2H), 6.78 – 6.74 (m, 4H), 4.37 – 4.28 (m, 3H), 3.89 – 3.85 (t, *J* = 6.0 Hz, 4H), 3.69 – 3.52 (m, 196H), 3.52 – 3.35 (m, 6H), 3.26 – 3.17 (m, 10H), 2.70 – 2.66 (m, 4H), 2.64 (s, 5H), 2.59 – 2.50 (m, 3H), 2.36 – 2.30 (m, 2H), 1.97 – 1.86 (m, 2H), 1.83 – 1.72 (m, 5H), 1.64 – 1.57 (m, 2H), 1.53 – 1.38 (s, 2H), 1.36 – 1.31 (m, 8H), 1.06 (t, *J* = 7.3 Hz, 6H).

^{13}C NMR (101 MHz, MeOD) δ 174.99, 174.90, 174.55, 173.90, 172.42, 153.26, 150.49, 145.27, 141.32, 140.16, 139.85, 138.24, 131.73, 128.71, 126.27, 125.59, 123.78, 121.25, 113.06, 72.23, 71.30, 71.20, 70.61, 70.57, 70.38, 68.20, 64.98, 54.29, 54.18, 52.34, 45.77, 43.29, 40.46, 40.44, 40.41, 38.75, 35.91, 35.86, 33.76, 33.23, 32.15, 31.86, 30.55, 29.10, 28.13, 27.75, 27.17, 26.50, 23.29, 22.63, 20.84, 18.64, 11.24, 9.20.

IR (neat) 3312 (b), 2867 (m), 1670 (w), 1599 (m), 1537 (m), 1454 (w), 1348 (w), 1300 (w), 1250 (m), 1104 (s), 950 (m), 848 (m) cm^{-1}.

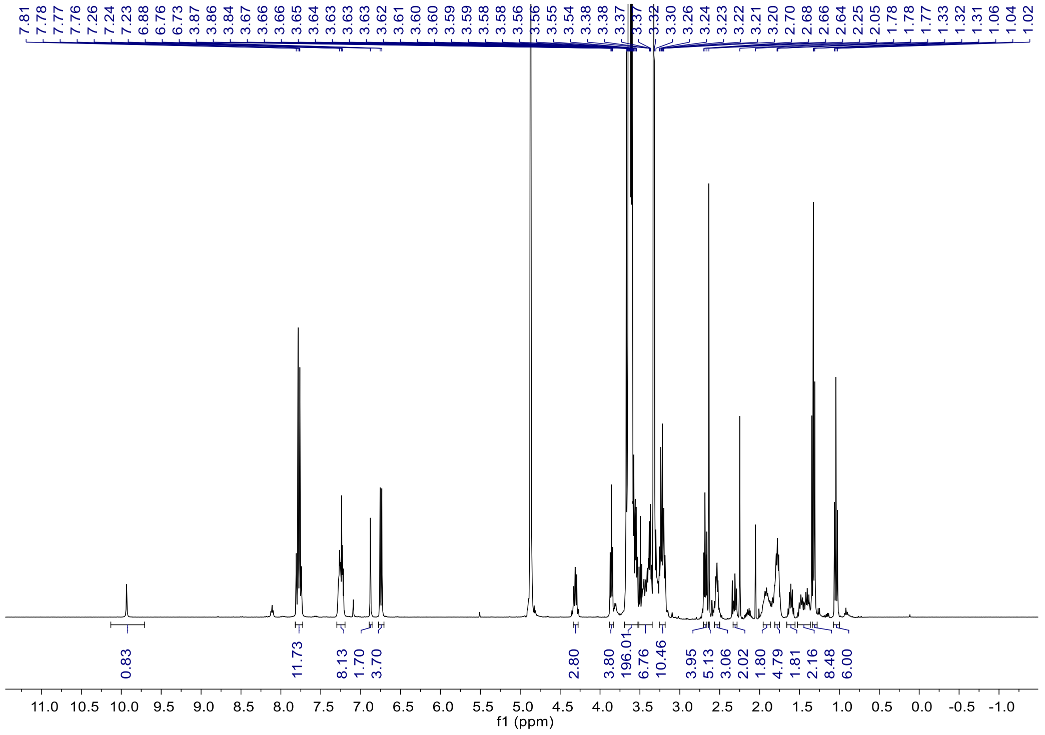

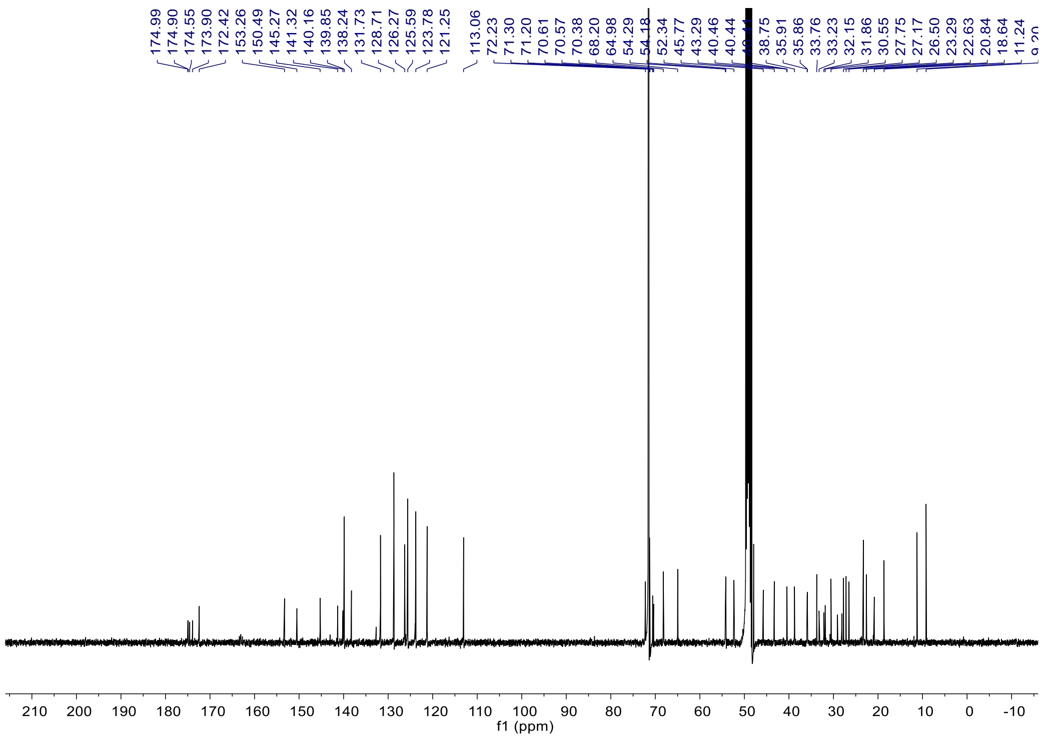

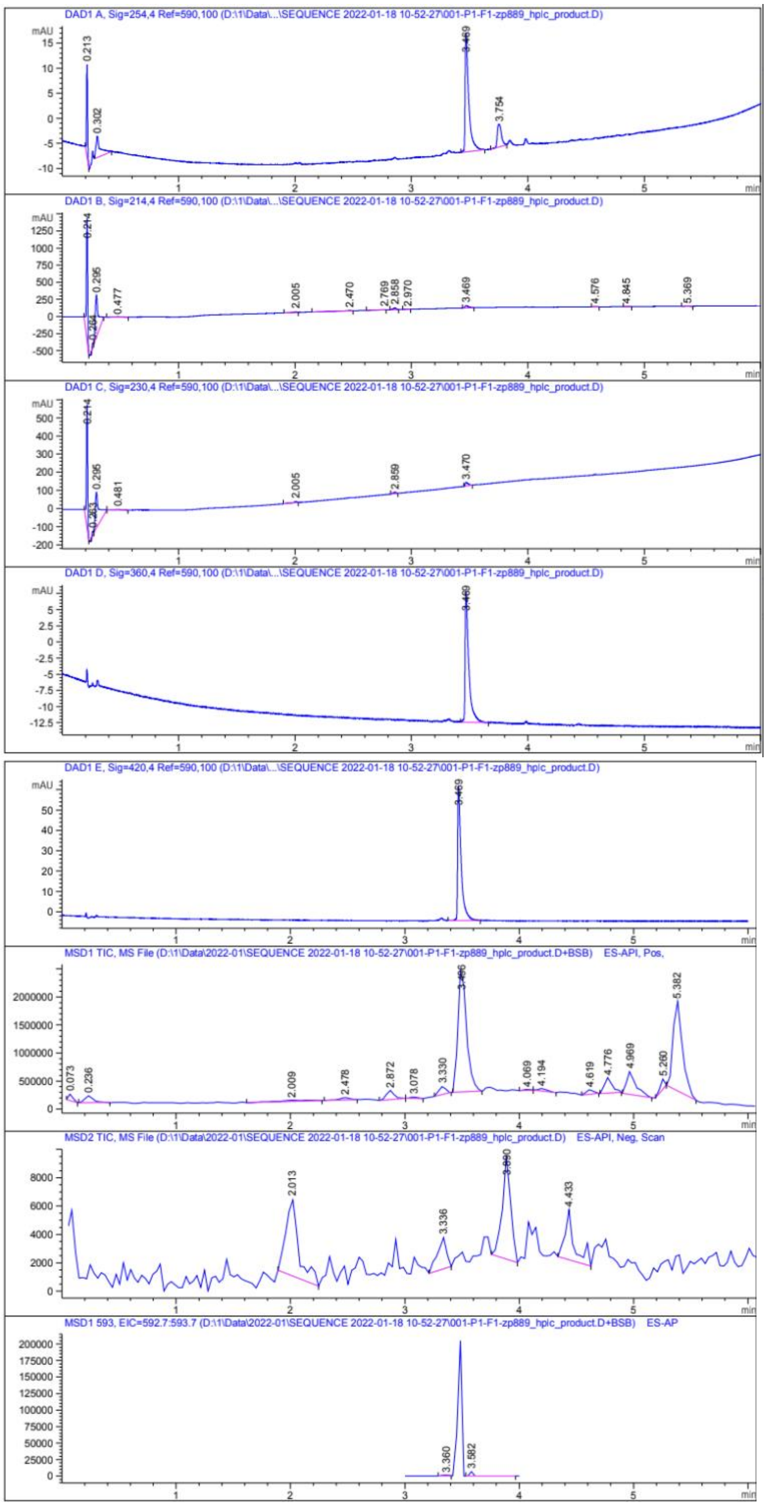
DAD1 A, Sig=254,4 Ref=590,100 (D:\1\Data\...\SEQUENCE 2022-01-18 10-52-27\001-P1-F1-zp889_hplc_product.D)
DAD1 B, Sig=214,4 Ref=590,100 (D:\1\Data\...\SEQUENCE 2022-01-18 10-52-27\001-P1-F1-zp889_hplc_product.D)
DAD1 C, Sig=230,4 Ref=590,100 (D:\1\Data\...\SEQUENCE 2022-01-18 10-52-27\001-P1-F1-zp889_hplc_product.D)
DAD1 D, Sig=360,4 Ref=590,100 (D:\1\Data\...\SEQUENCE 2022-01-18 10-52-27\001-P1-F1-zp889_hplc_product.D)
DAD1 E, Sig=420,4 Ref=590,100 (D:\1\Data\...\SEQUENCE 2022-01-18 10-52-27\001-P1-F1-zp889_hplc_product.D)
MSD1 TIC, MS File (D:\1\Data\2022-01\SEQUENCE 2022-01-18 10-52-27\001-P1-F1-zp889_hplc_product.D+BSB) ES-API, Pos,
MSD2 TIC, MS File (D:\1\Data\2022-01\SEQUENCE 2022-01-18 10-52-27\001-P1-F1-zp889_hplc_product.D) ES-API, Neg, Scan
MSD1 593, EIC=592.7:593.7 (D:\1\Data\2022-01\SEQUENCE 2022-01-18 10-52-27\001-P1-F1-zp889_hplc_product.D+BSB) ES-AP

1.2.7 S2: Dichlorophenylpiperazine Chloride

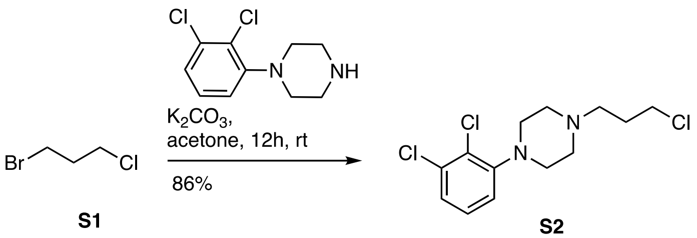

S2 was prepared accordingly to literature reported procedures (*Bioorg. Med. Chem.* **2016**, *24*, 2137-2145) and matched physicochemical properties.

In a 5 mL rbf, 2,3-dichlorophenylpiperazine (500 mg, 2.16 mmol, 1.00 equiv.) and K_2CO_3 (448 mg, 3.25 mmol, 1.50 equiv.) were treated with acetone (11 mL). 1-Bromo-3-chloropropane (**S1**) (0.728 mL, 7.35 mmol, 3.40 equiv.) was added under ambient conditions, and the reaction stirred for 12h. Then, the solids were filtered off, and the solvent removed under reduced pressure. The crude clear oil was purified by FCC (24g SiO_2, 0 -> 5% MeOH in DCM). The desired product **S2** was obtained as clear oil in 86% yield (573 mg, 1.86 mmol).

R_f = 0.61 (5% MeOH in DCM; UV detection).

HRMS (ESI): calc. for $C_{13}H_{17}Cl_3N_2Na^+$ $[M+Na]^+$: 329.0350; found 329.0350.

LCMS (5-100% MeCN in H_2O with 0.1% formic acid over 5 min) t_R = 2.789 min, 420 nm detection.

LRMS (ESI): calc. for $C_{13}H_{18}Cl_3N_2^+$ $[M+H]^+$: 307.1/309.1; found 308.0.

^{1}H NMR (400 MHz, $CDCl_3$) δ 7.2 – 7.1 (m, 2H), 7.0 (dd, *J* = 6.7, 2.9 Hz, 1H), 3.6 (t, *J* = 6.5 Hz, 2H), 3.1 (t, *J* = 4.7 Hz, 4H), 2.7 – 2.5 (m, 6H), 2.0 (p, *J* = 6.8 Hz, 2H).

^{13}C NMR (101 MHz, $CDCl_3$) δ 151.3, 134.2, 127.6, 127.6, 124.7, 118.7, 55.5, 53.4, 51.3, 43.3, 29.9.

IR (neat) 2944 (w), 2816 (w), 1679 (w), 1577 (s, 1445 (s), 1420 (m), 1373 (m), 1301 (w), 1259 (s), 1130 (s), 1044 (m), 1010 (m), 963 (s), 943 (s), 777 (s) cm^{-1}.

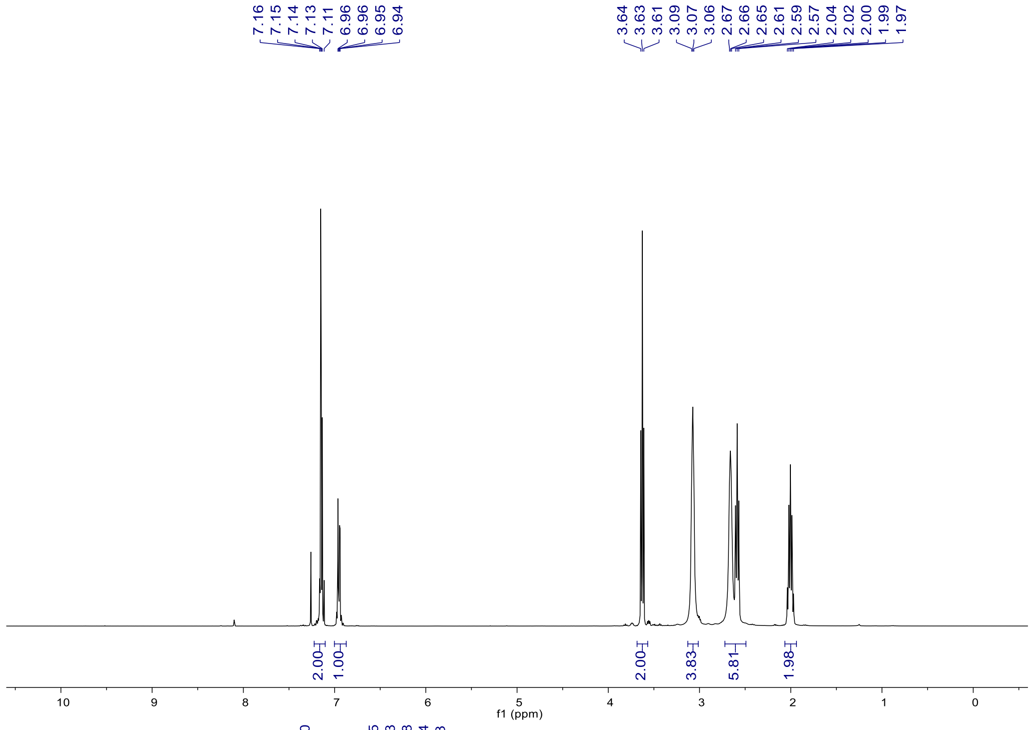
7.16
7.15
7.14
7.13
7.11
6.96
6.96
6.95
6.94
3.64
3.63
3.61
3.09
3.07
3.06
2.67
2.66
2.65
2.61
2.59
2.57
2.04
2.02
2.00
1.99
1.97
2.00
1.00
2.00
3.83
5.81
1.98
10
9
8
7
6
5
4
3
2
1
0
f1 (ppm)

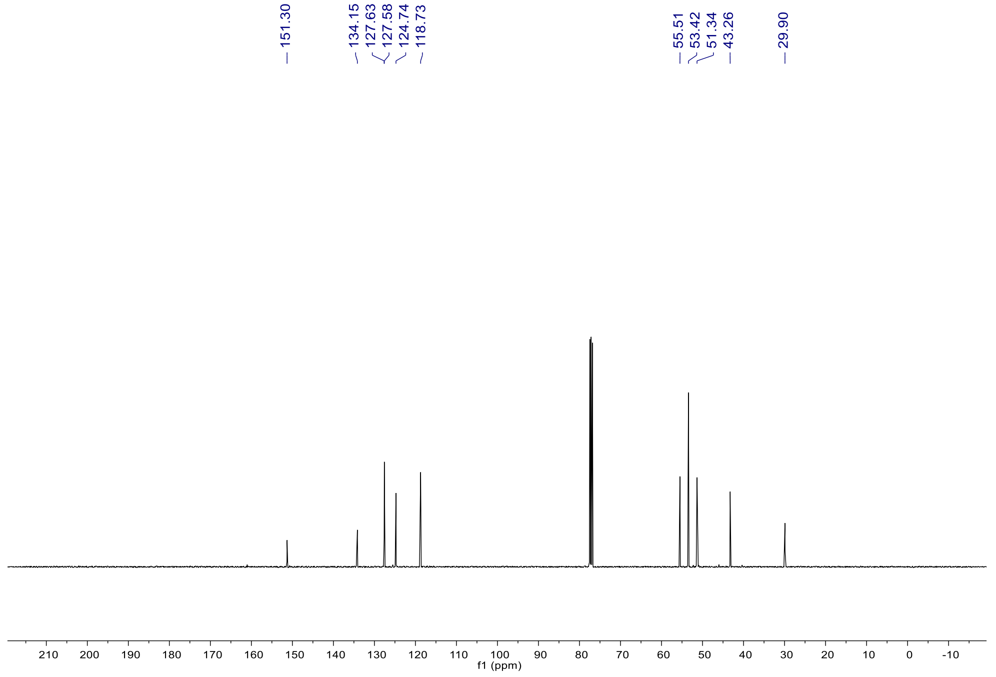
151.30
134.15
127.63
127.58
124.74
118.73
55.51
53.42
51.34
43.26
29.90
210
200
190
180
170
160
150
140
130
120
110
100
90
80
70
60
50
40
30
20
10
0
-10
f1 (ppm)

1.2.8 S3: Dichlorophenylpiperazine Azo

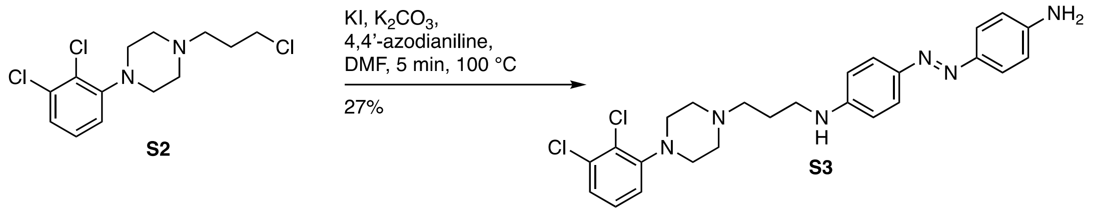

In a 20 mL vial, **S2** (250 mg, 0.813 mmol, 1.00 equiv.), KI (135 mg, 0.813 mmol, 1.00 equiv.), K_2CO_3 (225 mg, 1.63 mmol, 2.00 equiv.) and 4,4'-azodianiline (173 mg, 0.813 mmol, 1.00 equiv.) were dissolved in DMF (800 uL). The reaction was warmed to 100 °C for 5 min, and then let cool back to ambient temperature. The reaction mixture was then filtered, and the solvent removed under reduced pressure. The crude red oil was purified by FCC (24g, 0 -> 5% MeOH in DCM) to yield the desired product **S3** as red oil in 27% yield (106 mg, 0.219 mmol).

$\mathbf{R}_{\boldsymbol{f}}$ = 0.40 (5% MeOH in DCM, yellow spot).

HRMS (ESI): calc. for $C_{25}H_{29}Cl_2N_6^+$ $[M+H]^+$: 483.1825; found 483.1847.

LCMS (5-100% MeCN in H_2O with 0.1% formic acid over 5 min) t_R = 3.188 min, 420 nm detection.

LRMS (ESI): calc. for $C_{25}H_{29}Cl_2N_6^+$ $[M+H]^+$: 483.2; found 483.2.

^{1}H NMR (400 MHz, $CDCl_3$) δ 7.8 – 7.5 (m, 4H), 7.1 – 7.0 (m, 2H), 6.9 (dd, *J* = 7.0, 2.6 Hz, 1H), 6.7 – 6.5 (m, 4H), 3.2 (t, *J* = 6.3 Hz, 2H), 3.1 – 2.9 (m, 4H), 2.8 – 2.4 (m, 6H), 1.8 (t, *J* = 6.4 Hz, 2H).

^{13}C NMR (101 MHz, $CDCl_3$) δ 151.0, 150.5, 148.4, 146.0, 144.7, 134.1, 127.6, 127.5, 124.8, 124.6, 124.2, 118.7, 114.9, 112.2, 57.0, 53.3, 51.2, 43.0, 25.3.

IR (neat) 3383 (b), 3215 (b), 2943 (w), 2819 (w), 1722 (w), 1592 (s), 1514 (m), 1446 (m), 1303 (m), 1239 (s), 1145 (s), 1944 (w), 961 (w), 907 (w), 839 (m), 731 (m) cm^{-1}.

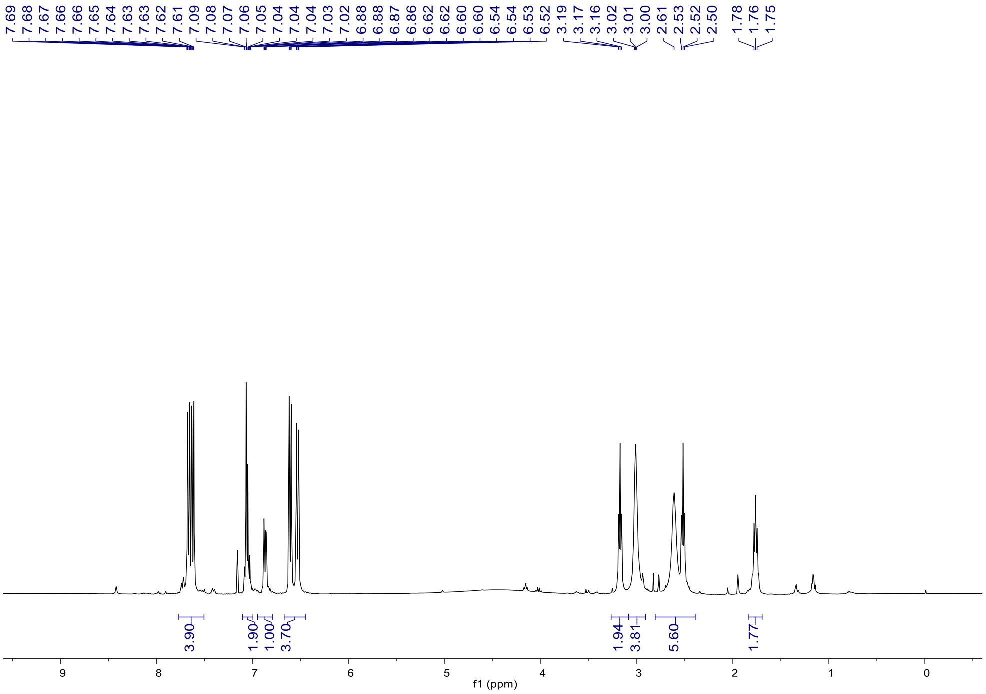
7.69 7.68 7.67 7.66 7.66 7.65 7.64 7.63 7.63 7.62 7.61 7.09 7.08 7.07 7.06 7.05 7.04 7.04 7.04 7.03 7.02 6.88 6.88 6.87 6.86 6.62 6.62 6.60 6.60 6.54 6.53 6.52 3.19 3.17 3.16 3.02 3.01 3.00 2.61 2.53 2.52 2.50 1.78 1.76 1.75
3.90 1.90 1.00 3.70 1.94 3.81 5.60 1.77
9 8 7 6 5 4 3 2 1 0
f1 (ppm)

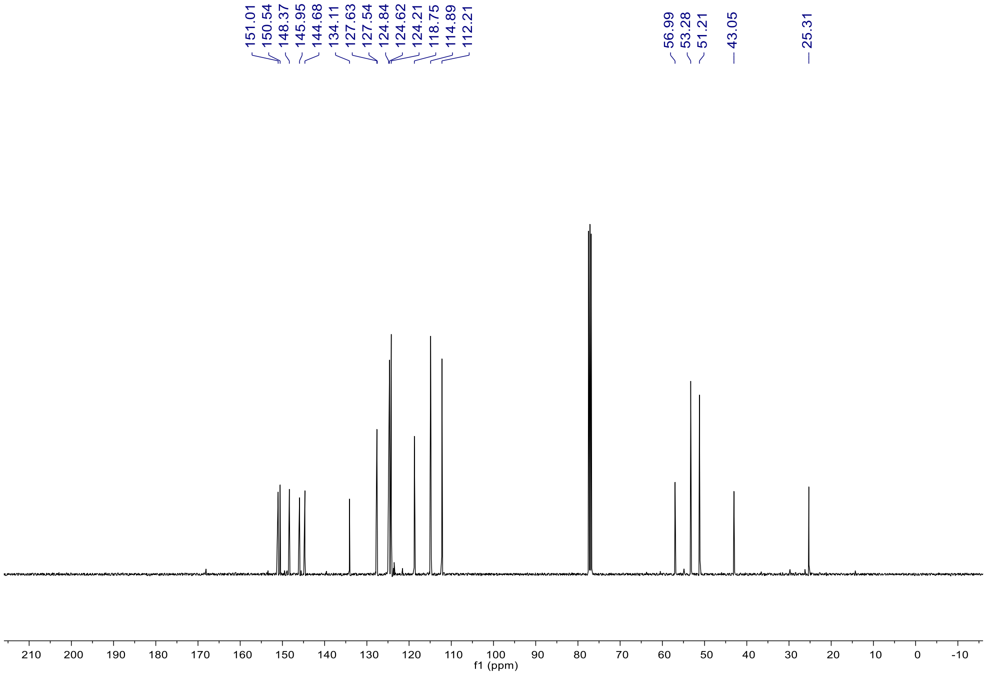
151.01 150.54 148.37 145.95 144.68 134.11 127.63 127.54 124.84 124.62 124.21 118.75 114.89 112.21
56.99 53.28 51.21 43.05 25.31
210 200 190 180 170 160 150 140 130 120 110 100 90 80 70 60 50 40 30 20 10 0 -10
f1 (ppm)

1.2.9 S4: Dichlorophenylpiperazine Azo PEG

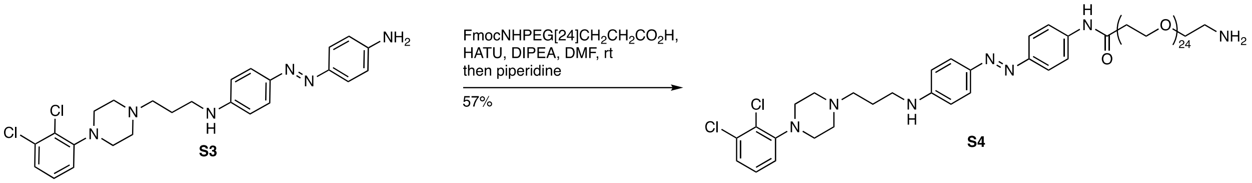

In a 20 mL vial, **S3** (10.0 mg, 0.021 mmol, 1.00 equiv.), NHFmocPEG[24]CH_2CH_2COOH (28.0 mg, 0.021 mmol, 1.00 equiv.), HATU (7.9 mg, 0.021 mmol, 1.00 equiv.) and DIPEA (18.0 uL, 0.103 mmol, 5.00 equiv.) were dissolved in DMF (300 uL). The reaction was stirred for 12h at ambient temperature until full conversion to the desired product was determined by LCMS, then, piperidine (40.0 uL, 0.410 mmol, 20.0 equiv.) was added. Deprotection was confirmed by LCMS and the reaction mixture subjected to RP-HPLC purification (semiprep column, 9 mL/min, 360 nm detection, 11 min runtime, 10-50% MeCN in water, 0.1% formic acid, t_R = 7.251) to yield the desired product **S4** as red oil in 57% yield (19.1 mg, 0.012 mmol).

<u>**NHFmoc intermediate:**</u>

LCMS (5-100% MeCN in H_2O with 0.1% formic acid over 5 min) t_R = 3.71 min, 360 nm detection.

LRMS (ESI): calc. for $C_{91}H_{141}Cl_2N_7O_{27}^{2+}$ $[M+2H]^{2+}$: 917.9; found 917.1.

<u>**Product**</u>:

HRMS (ESI): calc. for $C_{76}H_{131}Cl_2N_7O_{25}^{2+}$ $[M+2H]^{2+}$: 805.9280; found 805.9289.

LCMS (5-100% MeCN in H_2O with 0.1% formic acid over 5 min) t_R = 3.029 min, 360 nm detection.

LRMS (ESI): calc. for $C_{76}H_{131}Cl_2N_7O_{25}^{2+}$ $[M+2H]^{2+}$: 805.9; found 806.7.

^{1}H NMR (400 MHz, MeOD) δ 7.8 – 7.7 (m, 6H), 7.3 – 7.2 (m, 2H), 7.2 – 7.1 (m, 1H), 6.7 (d, *J* = 8.6 Hz, 2H), 3.9 – 3.8 (m, 2H), 3.8 (t, *J* = 5.1 Hz, 2H), 3.7 – 3.6 (m, 92H), 3.4 (s, 2H), 3.3 – 3.2 (m, 6H), 3.0 (s, 4H), 2.9 (t, *J* = 7.6 Hz, 2H), 2.7 (t, *J* = 5.9 Hz, 2H), 2.0 (t, *J* = 7.6 Hz, 2H).

^{13}C NMR (101 MHz, MeOD) δ 172.4, 153.2, 151.9, 150.6, 145.3, 141.3, 135.0, 129.2, 128.4, 126.3, 126.2, 123.8, 121.2, 120.3, 113.0, 71.6, 71.5, 71.4, 71.4, 71.4, 71.3, 71.3, 71.2, 71.2, 71.2, 71.1, 71.0, 70.8, 68.2, 68.0, 56.9, 54.1, 51.2, 42.0, 40.7, 38.7, 26.2.

IR (neat) 2866 (m), 1685 (w), 1595 (m), 1534 (m), 1448 (m), 1419 (w), 1346 (m), 1299 (m), 1245 (m), 1090 (s), 945 (m), 842 (s), 784 (w) cm^{-1}.

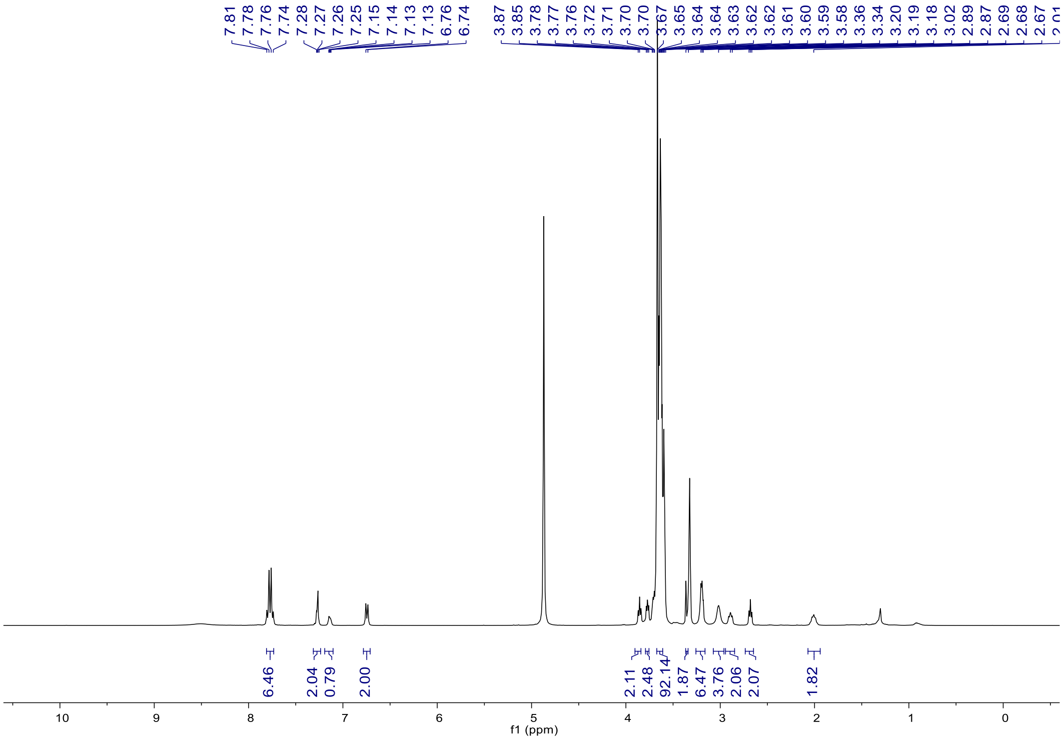
7.81
7.78
7.76
7.74
7.28
7.27
7.26
7.25
7.15
7.14
7.13
7.13
6.76
6.74
3.87
3.85
3.78
3.77
3.76
3.72
3.71
3.70
3.70
3.67
3.65
3.64
3.64
3.63
3.62
3.62
3.61
3.60
3.59
3.58
3.36
3.34
3.20
3.19
3.18
3.02
2.89
2.87
2.69
2.68
2.67
6.46
2.04
0.79
2.00
2.11
2.48
92.14
1.87
6.47
3.76
2.06
2.07
1.82
10
9
8
7
6
5
4
3
2
1
0
f1 (ppm)

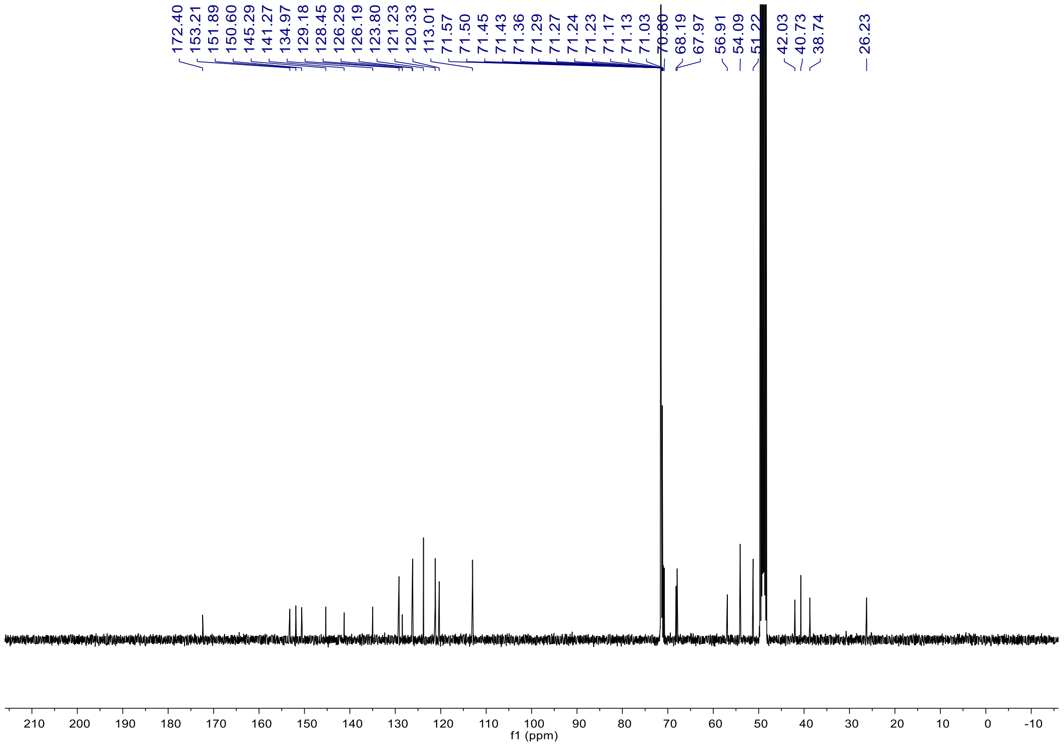
172.40
153.21
151.89
150.60
145.29
141.27
134.97
129.18
128.45
126.29
126.19
123.80
121.23
120.33
113.01
71.57
71.50
71.45
71.43
71.36
71.29
71.27
71.24
71.23
71.17
71.13
71.03
70.80
68.19
67.97
56.91
54.09
51.22
42.03
40.73
38.74
26.23
210
200
190
180
170
160
150
140
130
120
110
100
90
80
70
60
50
40
30
20
10
0
-10
f1 (ppm)

1.2.10 P-D2$_{p.ago}$

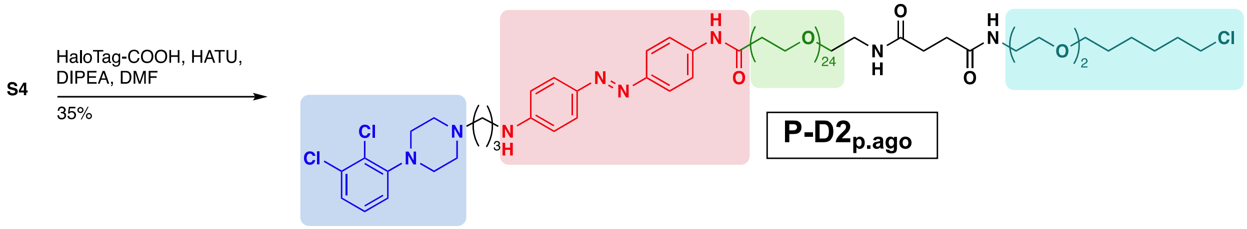

In a 4 mL vial, HaloTag-COOH (2.1 mg, 0.006 mmol, 1.00 equiv.) and **S4** (10.0 mg, 0.006 mmol, 1.00 equiv.) were dissolved in DMF (200 uL), and DIPEA (10 uL, 0.058 mmol, 9 equiv.) was added. The red solution was stirred for 1 h. Full conversion was determined by LCMS analysis, and the mixture was diluted and subjected to RP-HPLC (semiprep, 7 min run, 15-60% MeCN in water, containing 0.1% formic acid, 9 mL/min, t_R = 5.710 min, 360 nm detection). After evaporation of the solvent under reduced pressure, the desired product **P-D2$_{p.ago}$** was obtained as clear orange oil in 35% yield (4.1 mg, 2.1 umol).

HRMS (ESI): calc. for $C_{90}H_{153}Cl_3N_8K_2O_{29}^{2+}$ $[M+2K]^{2+}$: 993.4536; found 996.4520.

LCMS (5-100% MeCN in H_2O with 0.1% formic acid over 5 min) t_R = 3.640 min, 360 nm detection.

LRMS (ESI): calc. for $C_{90}H_{156}Cl_3N_8O_{29}^{3+}$ $[M+3H]^{3+}$: 640.2; found 639.3.

^{1}H NMR (400 MHz, MeOD) δ 7.8 – 7.7 (m, 6H), 7.3 – 7.2 (m, 2H), 7.1 (dd, *J* = 7.0, 2.6 Hz, 1H), 6.8 – 6.7 (m, 2H), 3.9 (t, *J* = 6.0 Hz, 2H), 3.7 – 3.5 (m, 112H), 3.4 – 3.4 (m, 2H), 3.2 – 3.1 (m, 4H), 3.0 – 2.8 (m, 4H), 2.8 (dd, *J* = 9.0, 6.3 Hz, 2H), 2.7 (t, *J* = 5.9 Hz, 2H), 2.6 (s, 1H), 2.5 (s, 4H), 2.0 – 1.9 (m, 2H), 1.8 – 1.8 (m, 2H), 1.6 – 1.6 (m, 2H), 1.5 – 1.5 (m, 2H), 1.4 – 1.4 (m, 2H).

^{13}C NMR (101 MHz, MeOD) δ 173.2, 171.0, 151.9, 150.8, 149.2, 143.8, 139.8, 133.5, 127.7, 127.0, 124.8, 124.7, 122.4, 119.8, 118.9, 111.6, 70.8, 70.2, 70.2, 70.1, 70.1, 70.0, 69.9, 69.9, 69.8, 69.8, 69.2, 66.8, 52.9, 50.3, 44.3, 44.3, 40.9, 39.0, 39.0, 37.4, 32.4, 30.8, 29.1, 26.3, 25.2, 25.1.

IR (neat) 3327 (b), 2866 (m), 1668 (w), 1599 (m), 1537 (m), 1449 (w), 1348 (m)., 1250 (w), 1136 (s), 1106 (s), 950 (w), 849 (w) cm^{-1}.

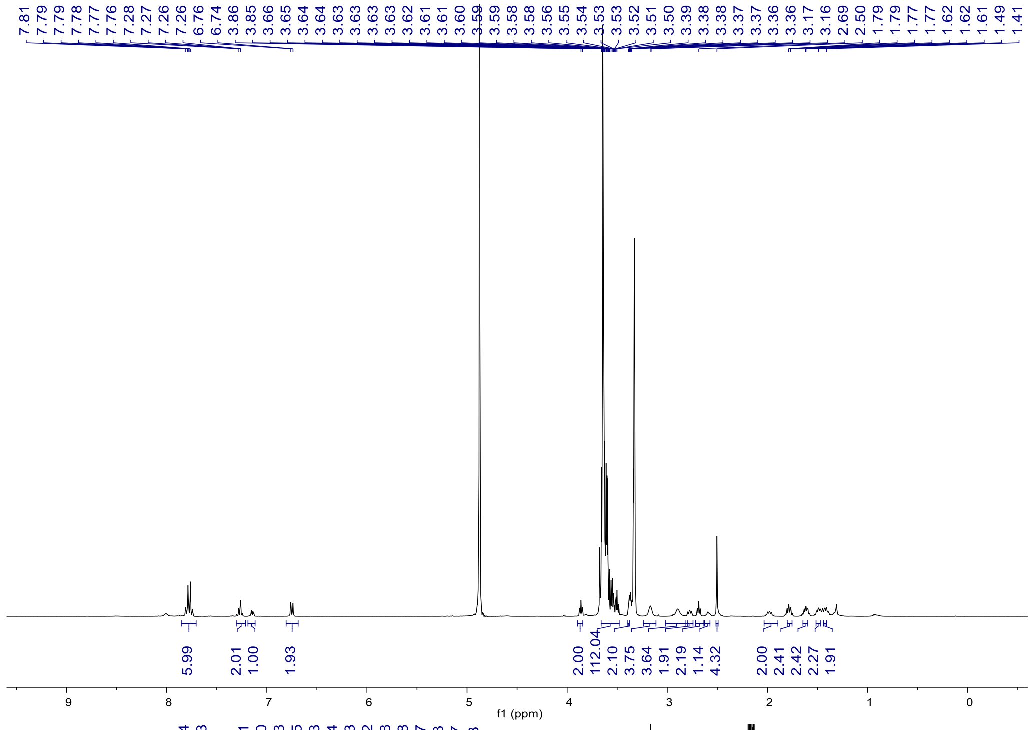
7.81 7.79 7.79 7.78 7.77 7.76 7.28 7.27 7.26 7.26 6.76 6.74 3.86 3.85 3.65 3.64 3.63 3.63 3.63 3.63 3.62 3.61 3.61 3.60 3.59 3.59 3.58 3.58 3.56 3.55 3.54 3.53 3.53 3.52 3.51 3.50 3.39 3.38 3.37 3.37 3.36 3.36 3.17 3.16 2.69 2.50 1.79 1.79 1.77 1.77 1.62 1.61 1.49 1.41
5.99 2.01 1.00 1.93 2.00 112.04 2.10 3.75 3.64 1.91 2.19 1.14 4.32 2.00 2.41 2.42 2.27 1.91
9 8 7 6 5 4 3 2 1 0
f1 (ppm)

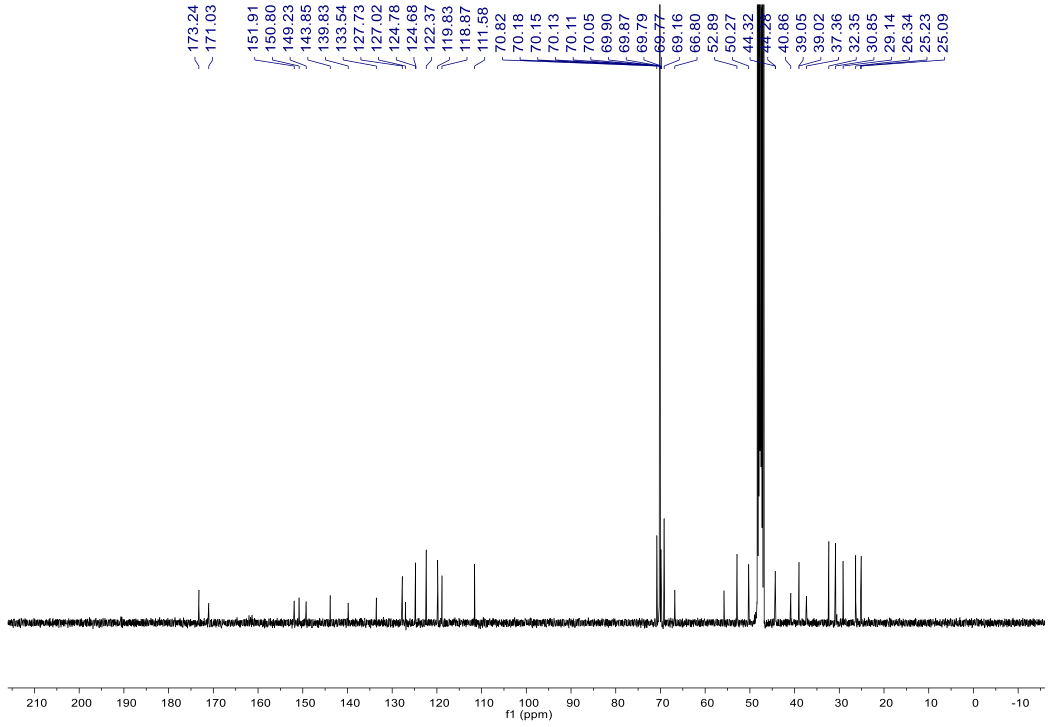
173.24 171.03 151.91 150.80 149.23 143.85 139.83 133.54 127.73 127.02 124.78 124.68 122.37 119.83 118.87 111.58 70.82 70.18 70.15 70.13 70.11 70.05 69.90 69.87 69.79 69.77 69.16 66.80 52.89 50.27 44.32 44.26 40.86 39.05 39.02 37.36 32.35 30.85 29.14 26.34 25.23 25.09
210 200 190 180 170 160 150 140 130 120 110 100 90 80 70 60 50 40 30 20 10 0 -10
f1 (ppm)

1.2.11 S5: 2X Dichlorophenylpiperazine Azo

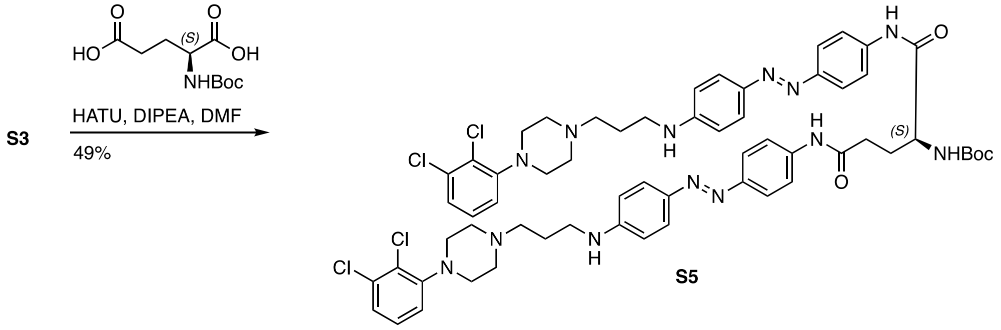

In a 4 mL vial, **S3** (64.5 mg, 0.133 mmol, 2.20 equiv), HATU (50.7 mg, 0.133 mmol, 2.20 equiv) and NHBoc-L-Glu-OH (15.0 mg, 0.061 mmol, 1.00 equiv) were dissolved in DMF (2 mL) and DIPEA (0.73 mL, 0.303 mmol, 5 equiv) was added. After 24h, the reaction mixture was subjected to FCC, and **S5** was isolated as red solid in 49% yield (35.7 mg, 0.030 mmol).

HRMS (ESI): calc. for $C_{60}H_{71}Cl_3N_{13}O_4^{2+}$ [M+H]$^{2+}$: 588.7248; found 588.7256.

LCMS (5-100% MeCN in H_2O with 0.1% formic acid over 5 min) t_R = 3.775 min, 360 nm detection.

LRMS (ESI): calc. for $C_{60}H_{71}Cl_3N_{13}O_4^{2+}$ [M+H]$^{2+}$: 588.8; found 589.8.

^{1}H NMR (400 MHz, $CDCl_3$) δ 7.8 (td, *J* = 6.2, 3.0 Hz, 7H), 7.7 (d, *J* = 8.5 Hz, 4H), 7.2 – 7.1 (m, 4H), 7.0 (dd, *J* = 6.0, 3.6 Hz, 2H), 6.6 (dd, *J* = 8.9, 2.1 Hz, 4H), 4.4 (dt, *J* = 12.1, 6.4 Hz, 1H), 3.3 (q, *J* = 5.7 Hz, 4H), 3.1 (s, 7H), 2.8 – 2.5 (m, 13H), 2.4 – 2.0 (m, 3H), 2.0 – 1.8 (m, 4H), 1.5 (s, 10H).

^{13}C NMR (101 MHz, $CDCl_3$) δ 172.0, 171.5, 171.4, 169.9, 153.1, 151.7, 151.3, 151.0, 149.8, 149.8, 144.6, 144.6, 138.9, 135.4, 134.2, 129.1, 127.7, 127.7, 125.7, 125.3, 125.0, 124.5, 123.3, 123.2, 120.2, 118.8, 112.3, 112.2, 112.1, 80.8, 57.1, 57.1, 53.4, 51.3, 51.2, 43.1, 29.8, 28.5, 28.4, 25.2, 25.2.

FTIR (neat) ν = 3320 (b), 2930 (w), 2822 (w), 1686 (m), 1599 (s), 1524 (m), 1448 (w), 1366 (w), 1303 (w), 1243 (m), 1138 (m), 961 (w), 845 (m).

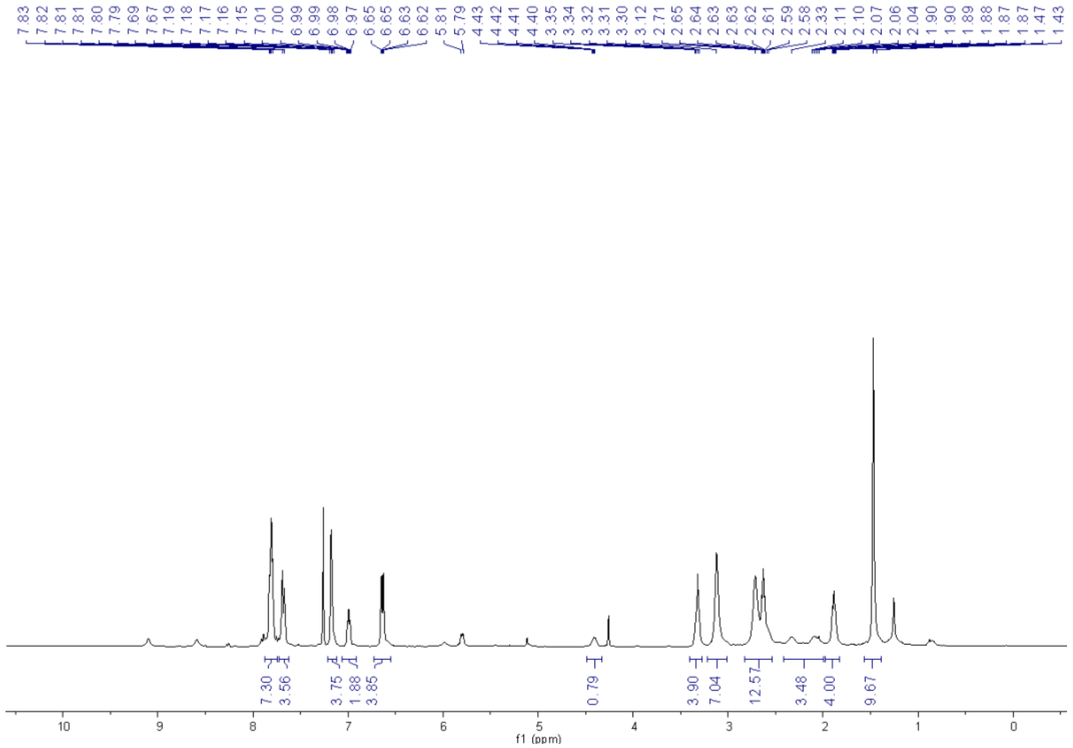
7.83 7.82 7.81 7.81 7.80 7.79 7.69 7.67 7.19 7.18 7.17 7.16 7.15 7.01 7.00 6.99 6.99 6.98 6.97 6.65 6.65 6.63 6.62 5.81 5.79 4.43 4.42 4.41 4.40 3.35 3.34 3.32 3.31 3.30 3.12 2.71 2.65 2.64 2.63 2.63 2.62 2.61 2.59 2.58 2.33 2.11 2.10 2.07 2.06 2.04 1.90 1.90 1.89 1.88 1.87 1.87 1.47 1.43
7.30 3.56 3.75 1.88 3.85 0.79 3.90 7.04 12.57 3.48 4.00 9.67
10 9 8 7 6 5 4 3 2 1 0
f1 (ppm)

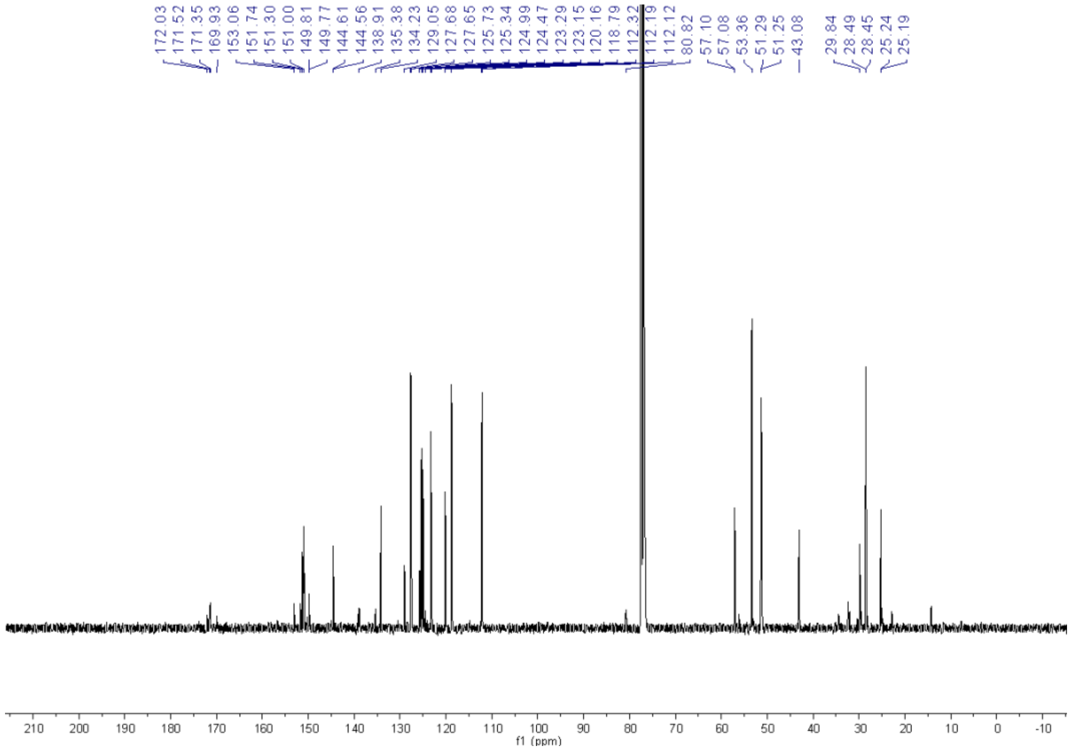
172.03 171.52 171.35 169.93 153.06 151.74 151.30 151.00 149.81 149.77 144.61 144.56 138.91 135.38 134.23 129.05 127.68 127.65 125.73 125.34 124.99 124.47 123.29 123.15 120.16 118.79 112.32 112.19 112.12 80.82 57.10 57.08 53.36 51.29 51.25 43.08 29.84 28.49 28.45 25.24 25.19
210 200 190 180 170 160 150 140 130 120 110 100 90 80 70 60 50 40 30 20 10 0 -10
f1 (ppm)

1.2.12 S6: 2X Dichlorophenylpiperazine Azo PEG

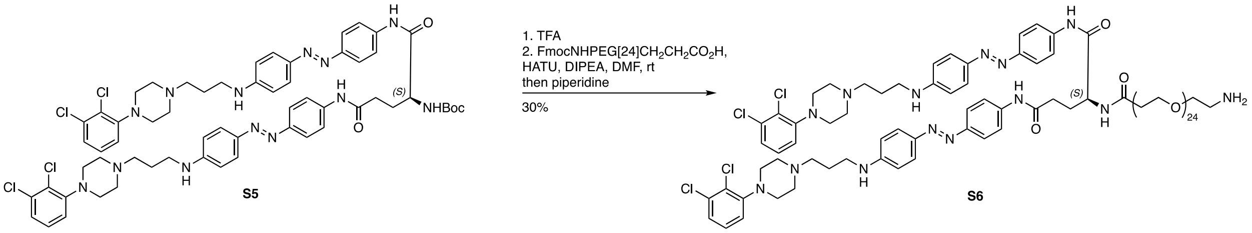

In a 4 mL vial, **S5** (9.5 mg, 0.008 mmol, 1.0 equiv), was dissolved in DCM (0.5 mL), and TFA (0.5 mL) was added. After, 1 min, LCMS showed full conversion to the amine. The solvent was removed under reduced pressure, then the crude mixture was taken up in DMF (1 mL). HATU (3.2 mg, 0.008 mmol, 1.0 equiv), FmocNHPEG[24]CH_2CH_2COOH (12.8 mg, 0.009 mmol, 1.1 equiv) and DIPEA (15 uL, 0.085 mmol, 10 equiv) were added. After 24h, LCMS showed full conversion to the product, and piperidine (28 uL, 0.28 mmol, 33 equiv) was added to the reaction mixture. After 1h, the mixture was concentrated under reduced pressure, and the crude material subjected to RP-HPLC (20-50% MeCN in water, containing 0.1% forminc acid) to yield **S6** after lyophilization in 30% yield (5.6 mg, 0.003 mmol).

HRMS (ESI): calc. for $C_{106}H_{164}Cl_4N_{14}O_{27}^{2+}$ $[M+2H]^{2+}$: 1103.0332; found 1103.0426.

LCMS (5-100% MeCN in H_2O with 0.1% formic acid over 5 min) t_R = 3.410 min, 360 nm detection.

LRMS (ESI): calc. for $C_{106}H_{165}Cl_4N_{14}O_{27}^{3+}$ $[M+3H]^{3+}$: 553.2; found 552.5.

^{1}H NMR (400 MHz, $CDCl_3$) δ 7.8 (s, 11H), 7.2 (d, *J* = 1.4 Hz, 4H), 7.0 (dd, *J* = 5.4, 4.2 Hz, 2H), 6.8 – 6.6 (m, 4H), 4.7 (s, 1H), 3.9 – 3.4 (m, 91H), 3.3 (t, *J* = 6.3 Hz, 4H), 3.1 (s, 7H), 2.7 – 2.6 (m, 13H), 2.2 (q, *J* = 7.3 Hz, 13H), 1.9 (d, *J* = 6.4 Hz, 4H).

^{13}C NMR (101 MHz, $CDCl_3$) δ 172.5, 172.1, 170.0, 151.3, 151.2, 149.4, 149.1, 144.6, 140.5, 134.2, 127.7, 125.3, 125.2, 124.9, 123.2, 123.0, 120.3, 119.7, 118.7, 112.3, 112.2, 70.7, 70.7, 70.6, 70.6, 70.4, 70.4, 70.3, 57.4, 53.5, 51.6, 43.4, 37.2, 25.5.

FTIR (neat) ν = 3319 (b), 2871 (w), 1683 (w), 1596 (s), 1533 (m), 1448 (w), 1346 (w), 1301 (m),1243 (m), 1136 (s), 1104 (s), 1044 (w), 949 (w), 845 (m), 781 (m).

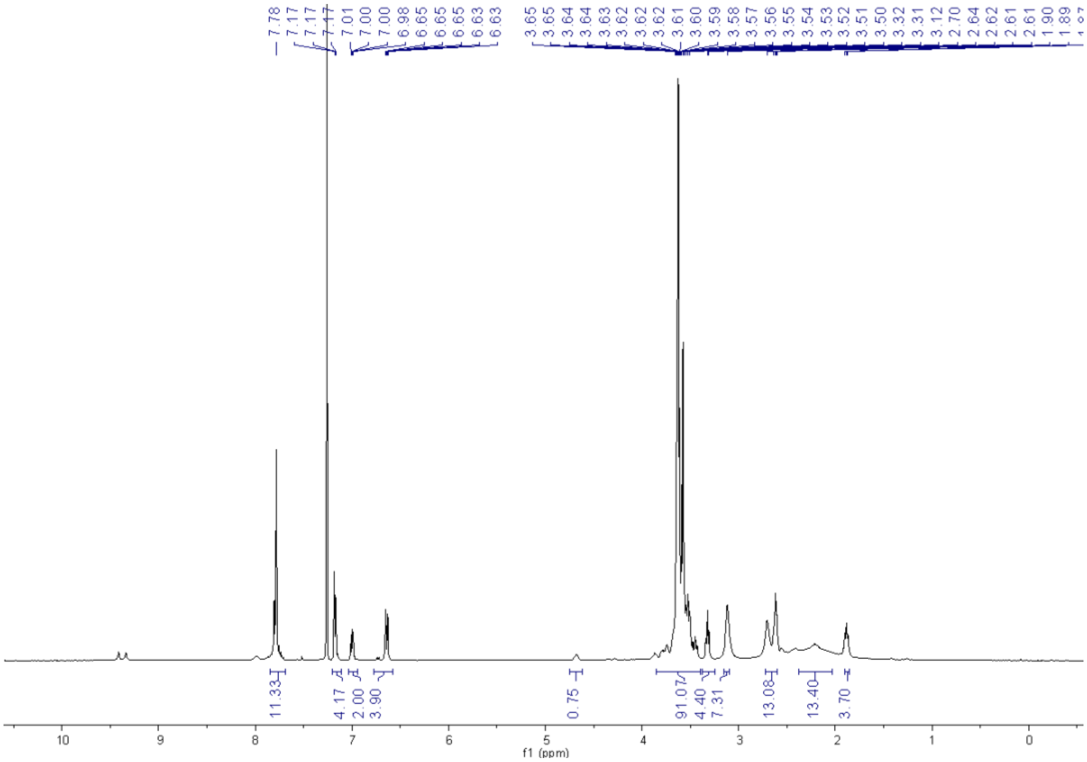
7.78
7.17
7.17
7.17
7.01
7.00
7.00
6.98
6.65
6.65
6.65
6.63
6.63
3.65
3.65
3.64
3.64
3.63
3.62
3.62
3.62
3.61
3.60
3.59
3.58
3.57
3.56
3.55
3.54
3.53
3.52
3.51
3.50
3.32
3.31
3.12
2.70
2.64
2.62
2.61
2.61
1.90
1.89
11.33
4.17
2.00
3.90
0.75
91.07
4.40
7.31
13.08
13.40
3.70
10
9
8
7
6
5
4
3
2
1
0
f1 (ppm)

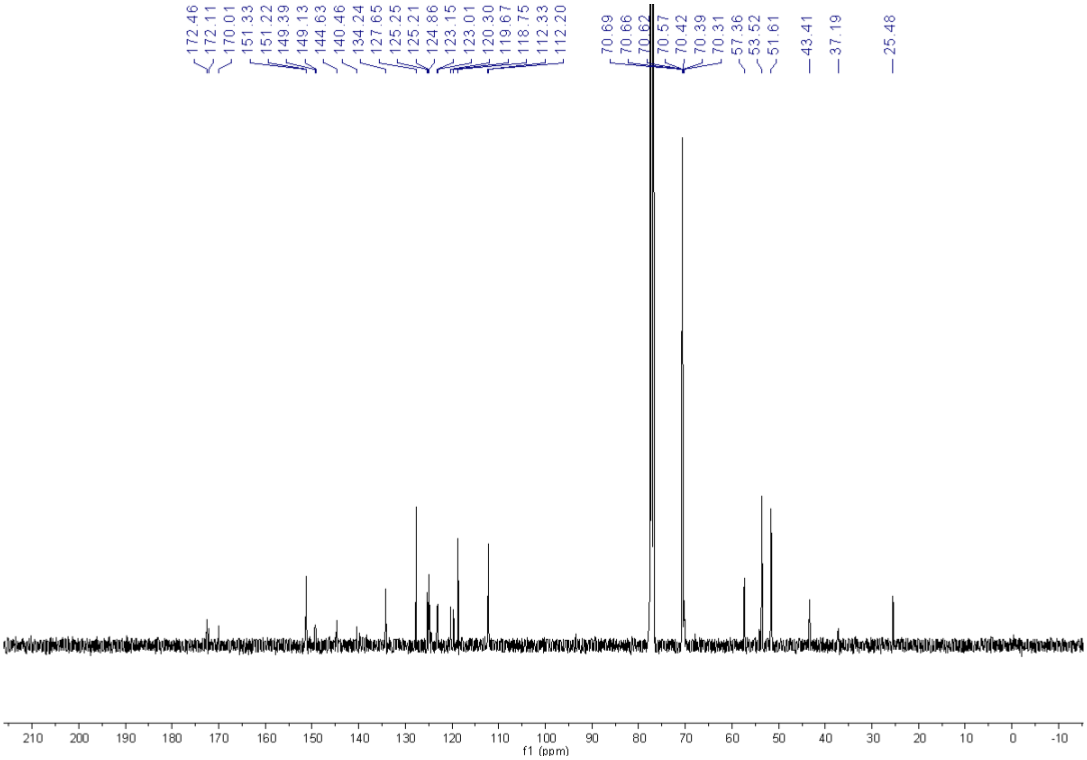
172.46
172.11
170.01
151.33
151.22
149.39
149.13
144.63
140.46
134.24
127.65
125.25
125.21
124.86
123.15
123.01
120.30
119.67
118.75
112.33
112.20
70.69
70.66
70.62
70.57
70.42
70.39
70.31
57.36
53.52
51.61
43.41
37.19
25.48
210
200
190
180
170
160
150
140
130
120
110
100
90
80
70
60
50
40
30
20
10
0
-10
f1 (ppm)

1.2.13 P-D2$_{p.ago}$-2X

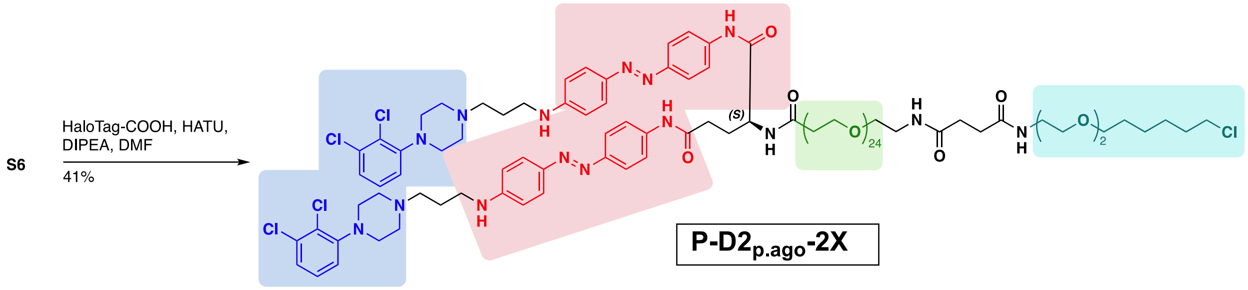

In a 4 mL vial, **S6** (5.3 mg, 0.002 mmol, 1.0 equiv), HATU (0.8 mg, 0.002 mmol, 1.0 equiv) and HaloTag-COOH (0.9 mg, 0.002 mmol, 1.0 equiv) were dissolved in DMF (0.5 mL) and DIPEA (2 µL, 0.002 mmol, 5 equiv) was added. After 24h, the reaction mixture was subjected to RP-HPLC, and **P-D2$_{p.ago}$-2X** was isolated as red solid in 41% yield (2.5 mg, 0.001 mmol).

HRMS (ESI): calc. for $C_{120}H_{186}Cl_5N_{15}O_{31}^{2+}$[M+2]$^{2+}$: 1255.0938; found 1255.0952.

LCMS (5-100% MeCN in H2O with 0.1% formic acid over 5 min) t_R = 3.737min, 360 nm detection.

LRMS (ESI): calc. for $C_{120}H_{189}C_{l5}N_{15}O_{31}^{3+}$ [M+3H]$^{3+}$: 838.1; found 838.6.

^{1}H NMR (400 MHz, $CDCl_3$) δ 7.8 (d, *J* = 9.4 Hz, 11H), 7.2 – 7.2 (m, 4H), 7.0 (dd, *J* = 5.9, 3.6 Hz, 2H), 6.7 – 6.6 (m, 4H), 4.7 (d, *J* = 4.8 Hz, 1H), 3.9 (s, 1H), 3.8 (dd, *J* = 8.8, 4.8 Hz, 2H), 3.7 – 3.5 (m, 107H), 3.4 (t, *J* = 6.3 Hz, 4H), 3.2 (s, 7H), 2.8 – 2.6 (m, 15H), 2.5 (s, 4H), 2.3 (dd, *J* = 87.1, 7.6 Hz, 4H), 1.9 (s, 2H), 1.8 (s, 2H), 1.6 (s, 2H), 1.5 (d, *J* = 7.9 Hz, 2H), 1.4 (d, *J* = 7.2 Hz, 2H).

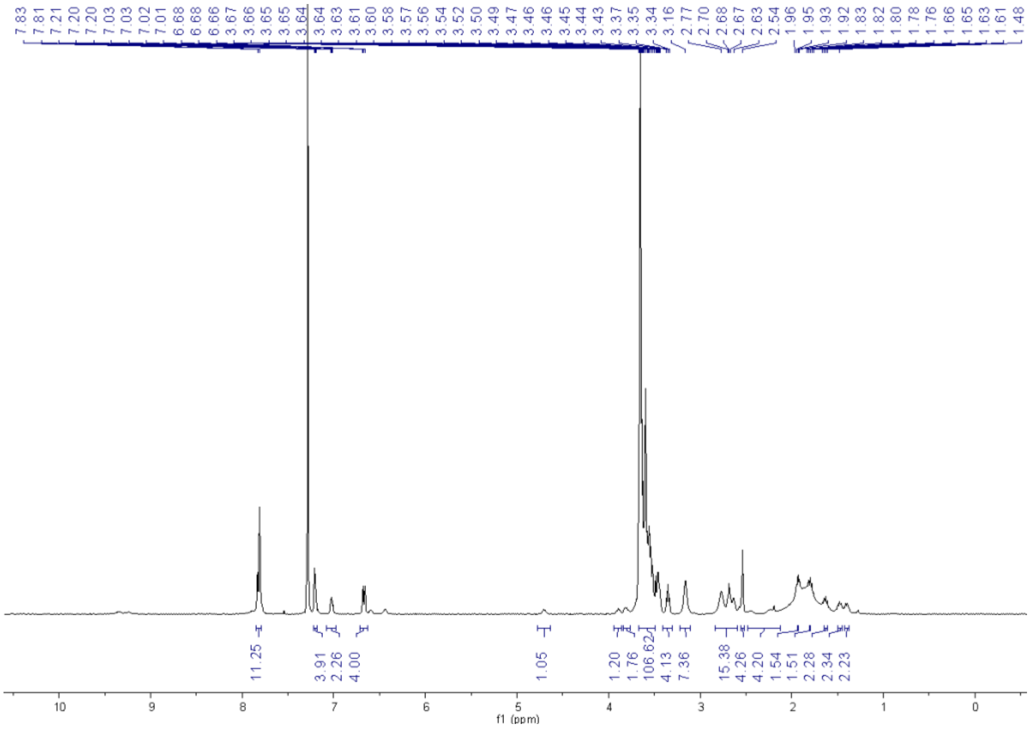

7.83 7.81 7.21 7.20 7.20 7.03 7.03 7.02 7.01 6.68 6.68 6.66 3.67 3.66 3.65 3.65 3.64 3.64 3.63 3.61 3.60 3.58 3.57 3.56 3.54 3.52 3.50 3.49 3.47 3.46 3.46 3.45 3.44 3.43 3.37 3.35 3.34 3.16 2.77 2.70 2.68 2.67 2.63 2.54 1.96 1.95 1.93 1.92 1.83 1.82 1.80 1.78 1.76 1.66 1.65 1.63 1.61 1.48
11.25 3.91 2.26 4.00 1.05 1.20 1.76 106.62 4.13 7.36 15.38 4.26 4.20 1.54 1.51 2.28 2.34 2.23
10 9 8 7 6 5 4 3 2 1 0
f1 (ppm)

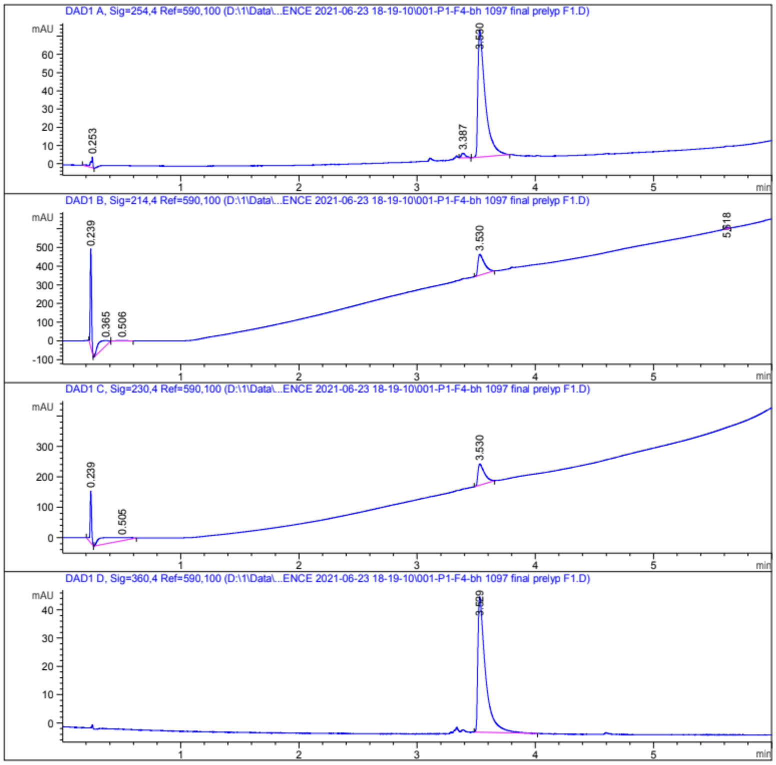
DAD1 A, Sig=254,4 Ref=590,100 (D:\1\Data\...ENCE 2021-06-23 18-19-10\001-P1-F4-bh 1097 final prelyp F1.D)
DAD1 B, Sig=214,4 Ref=590,100 (D:\1\Data\...ENCE 2021-06-23 18-19-10\001-P1-F4-bh 1097 final prelyp F1.D)
DAD1 C, Sig=230,4 Ref=590,100 (D:\1\Data\...ENCE 2021-06-23 18-19-10\001-P1-F4-bh 1097 final prelyp F1.D)
DAD1 D, Sig=360,4 Ref=590,100 (D:\1\Data\...ENCE 2021-06-23 18-19-10\001-P1-F4-bh 1097 final prelyp F1.D)

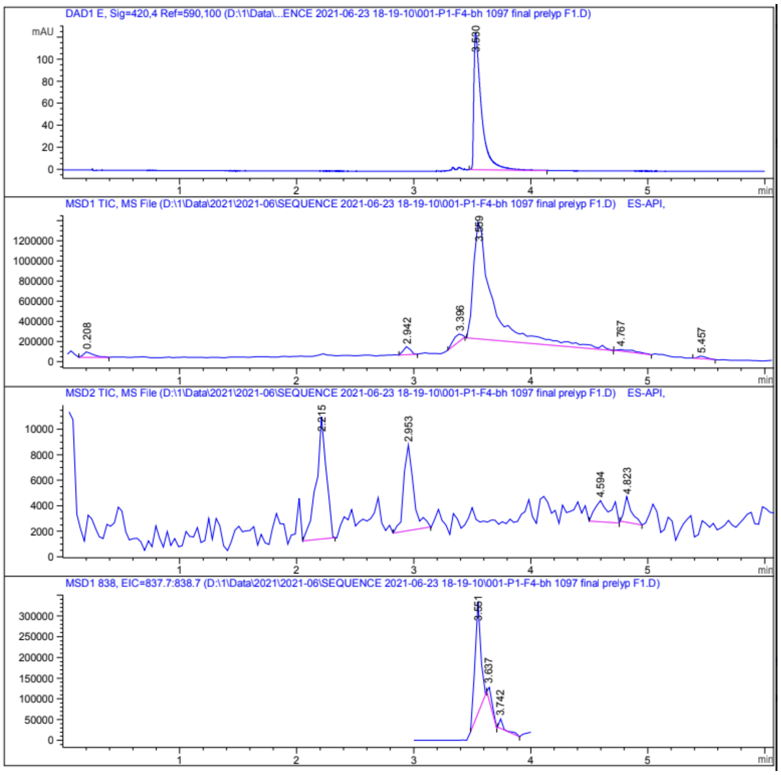
DAD1 E, Sig=420,4 Ref=590,100 (D:\1\Data\...ENCE 2021-06-23 18-19-10\001-P1-F4-bh 1097 final prelyp F1.D)
MSD1 TIC, MS File (D:\1\Data\2021\2021-06\SEQUENCE 2021-06-23 18-19-10\001-P1-F4-bh 1097 final prelyp F1.D) ES-API,
MSD2 TIC, MS File (D:\1\Data\2021\2021-06\SEQUENCE 2021-06-23 18-19-10\001-P1-F4-bh 1097 final prelyp F1.D) ES-API,
MSD1 838, EIC=837.7:838.7 (D:\1\Data\2021\2021-06\SEQUENCE 2021-06-23 18-19-10\001-P1-F4-bh 1097 final prelyp F1.D)

1.2.14 S7: Methoxyphenylpiperazine Chloride

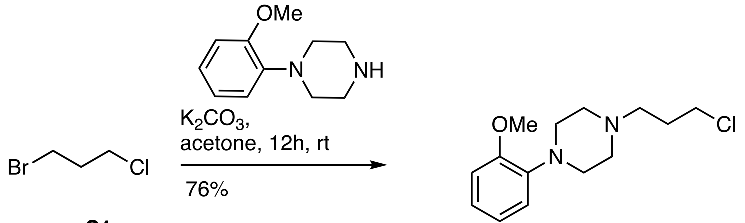

S1 S7

13 was prepared accordingly to literature reported procedures (*Bioorg. Chem*. **2018**, *77*, 125-135) and matched physicochemical properties.

In a 5 mL rbf, 1-(2-methoxyphenyl)piperazine (200 mg, 1.04 mmol, 1.00 equiv.) and K_2CO_3 (215 mg, 1.56 mmol, 1.50 equiv.) were treated with acetone (5.2 mL). 1-Bromo-3-chloropropane **S1** (113 uL, 1.14 mmol, 1.10 equiv.) was added dropwise under ambient conditions, and the reaction stirred for 5h. Then, more **S1** (31 uL, 0.31 mmol, 0.30 equiv.) was added, and the reaction was stirred for 12h. Then, the solids were filtered off, and the solvent removed under reduced pressure. The crude clear oil was purified by FCC (4g SiO_2, 0 -> 5% MeOH in DCM). The desired product **S7** was obtained as clear oil in 76% yield (212 mg, 0.789 mmol).

HRMS (ESI): calc. for $C_{14}H_{22}ClN_2O^+$ [M+H]$^+$: 269.1415; found 269.1424.

LCMS (5-100% MeCN in H_2O with 0.1% formic acid over 5 min) t_R = 2.112 min, 254 nm detection.

LRMS (ESI): calc. for $C_{14}H_{22}ClN_2O^+$ [M+H]$^+$: 269.1; found 269.1.

^{1}H NMR (400 MHz, $CDCl_3$) δ 7.0 – 6.8 (m, 4H), 3.9 (s, 3H), 3.6 (t, *J* = 6.6 Hz, 2H), 3.1 (s, 4H), 2.6 (t, *J* = 4.8 Hz, 4H), 2.6 (t, *J* = 7.1 Hz, 2H), 2.0 – 2.0 (m, 2H).

^{13}C NMR (101 MHz, $CDCl_3$) δ 152.3, 141.4, 122.9, 121.0, 118.2, 111.2, 55.6, 55.4, 53.5, 50.7, 43.3, 30.0.

IR (neat) 2940 (w), 2812 (w), 1593 (m) 1498 (s), 1446 (m), 1355 (w), 1300 (m), 1238 (s), 1179 (m), 1133 (s), 1058 (m), 1024 (m), 955 (w), 924 (w) cm^{-1}.

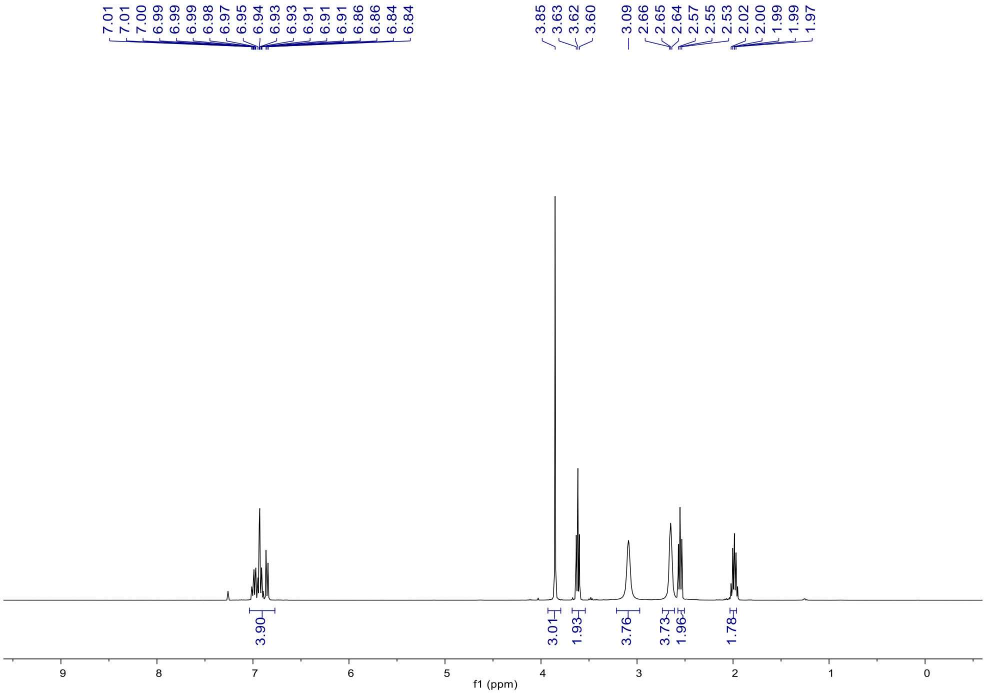
7.01 7.01 7.00 6.99 6.99 6.99 6.98 6.97 6.95 6.94 6.93 6.93 6.91 6.91 6.91 6.86 6.86 6.84 6.84
3.85 3.63 3.62 3.60
3.09 2.66 2.65 2.64 2.57 2.55 2.53 2.02 2.00 1.99 1.99 1.97
3.90
3.01 1.93 3.76 3.73 1.96 1.78
f1 (ppm)

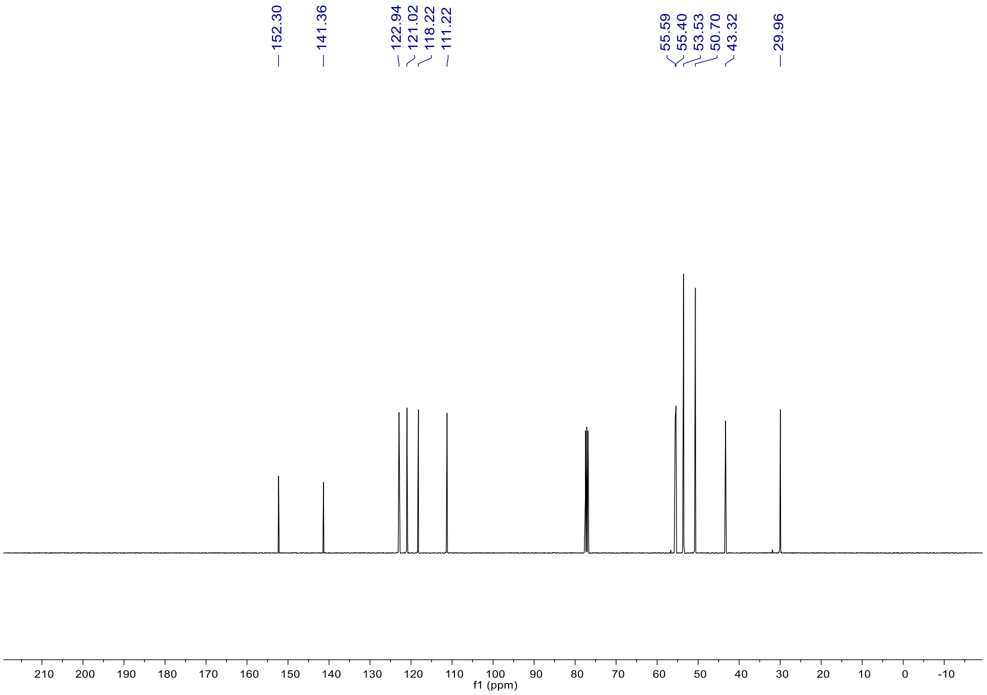
152.30
141.36
122.94 121.02 118.22 111.22
55.59 55.40 53.53 50.70 43.32
29.96
f1 (ppm)

1.2.15 S8: Methoxyphenylpiperazine Azo

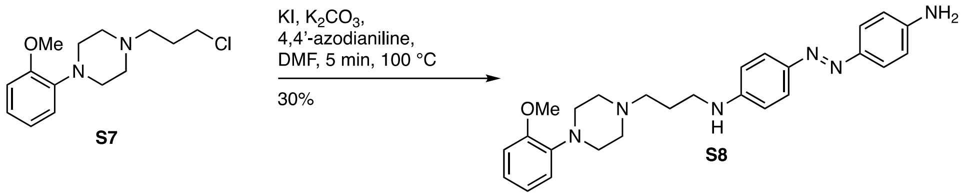

In a 20 mL vial, **S7** (118 mg, 0.440 mmol, 1.00 equiv.), KI (73.1mg, 0.440 mmol, 1.00 equiv.), K_2CO_3 (122 mg, 0.881 mmol, 2.00 equiv.) and 4,4'-azodianiline (93.5 mg, 0.440 mmol, 1.00 equiv.) were dissolved in DMF (440 uL). The reaction was warmed to 100 °C for 1h. The reaction mixture was then cooled and filtered, and the solvent removed under reduced pressure. The crude red oil was purified by FCC (4g, 0-5% MeOH in DCM) to yield the desired product **S8** as red oil in 30% yield (58.5 mg, 0.132 mmol).

HRMS (ESI): calc. for $C_{26}H_{32}N_6ONa^+$ $[M+Na]^+$: 467.2530; found 467.2533.

LCMS (5-100% MeCN in H_2O with 0.1% formic acid over 5 min) t_R = 2.744 min, 360 nm detection.

LRMS (ESI): calc. for for $C_{26}H_{33}N_6O^+$ $[M+H]^+$: 445.3; found 445.3.

^{1}H NMR (400 MHz, MeOD) δ 7.7 – 7.5 (m, 4H), 7.1 – 6.8 (m, 4H), 6.8 – 6.6 (m, 4H), 3.8 (s, 3H), 3.2 (t, *J* = 6.7 Hz, 2H), 3.1 (s, 4H), 2.7 (s, 4H), 2.6 (dd, *J* = 8.9, 6.3 Hz, 2H), 1.9 (t, *J* = 7.5 Hz, 2H).

^{13}C NMR (101 MHz, MeOD) δ 153.8, 152.4, 151.7, 146.2, 145.3, 142.1, 125.3, 125.0, 124.7, 122.2, 119.4, 115.5, 113.1, 112.8, 57.5, 55.9, 54.3, 51.5, 42.7, 26.9.

IR (neat) 3338 (b), 2941 (w), 2818 (w), 1666 (m), 1593 (s), 1498 (m), 1449 (w), 1385 (w), 1332 (w), 1303 (w), 1238 (s), 1180 (w), 1144 (s), 1025 (m), 906 (m), 832 (m) cm^{-1}.

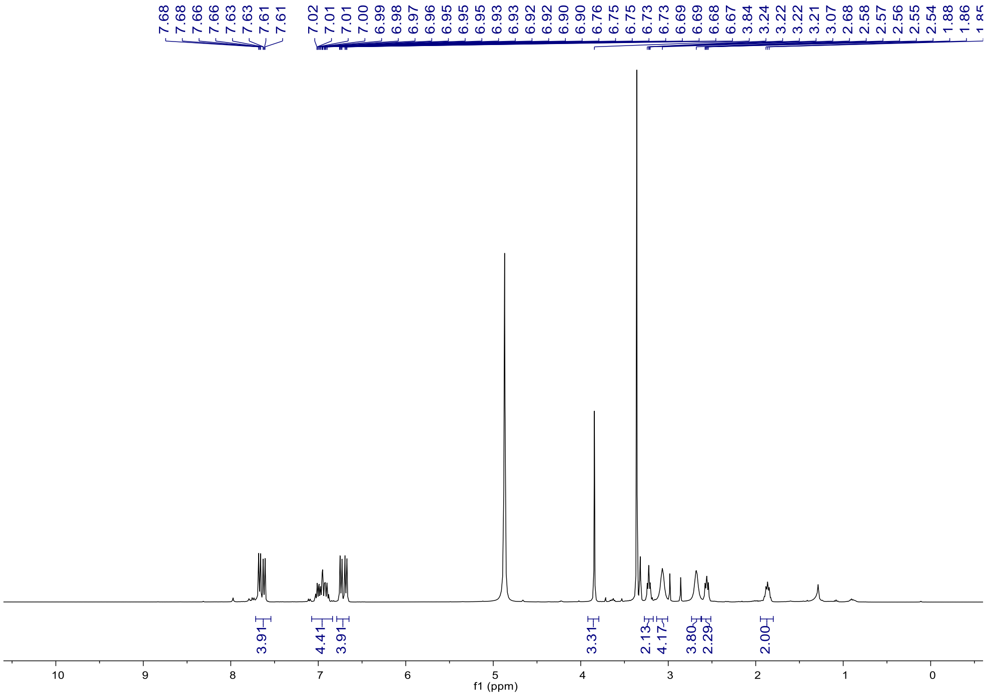
7.68
7.68
7.66
7.66
7.63
7.63
7.61
7.61
7.02
7.01
7.01
7.00
6.99
6.98
6.97
6.96
6.95
6.95
6.95
6.93
6.93
6.92
6.92
6.90
6.90
6.76
6.75
6.75
6.73
6.73
6.69
6.69
6.68
6.67
3.84
3.24
3.22
3.22
3.21
3.07
2.68
2.58
2.57
2.56
2.55
2.54
1.88
1.86
3.91
4.41
3.91
3.31
2.13
4.17
3.80
2.29
2.00
10
9
8
7
6
5
4
3
2
1
0
f1 (ppm)

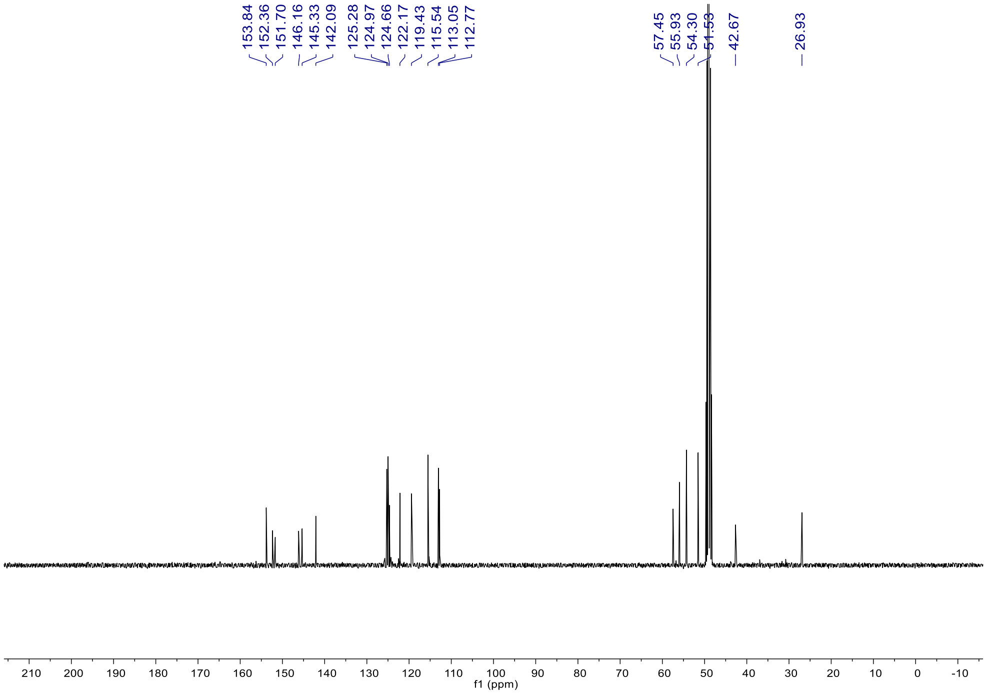
153.84
152.36
151.70
146.16
145.33
142.09
125.28
124.97
124.66
122.17
119.43
115.54
113.05
112.77
57.45
55.93
54.30
51.53
42.67
26.93
210
200
190
180
170
160
150
140
130
120
110
100
90
80
70
60
50
40
30
20
10
0
-10
f1 (ppm)

1.2.16 S9: Methoxyphenylpiperazine Azo PEG

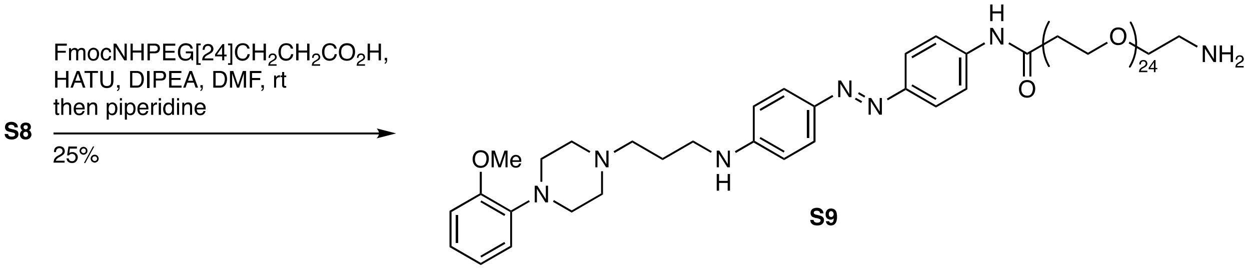

In a 10 mL rbf, $FmocNHCH_2CH_2PEG[24]CO_2H$ (96 mg, 0.070 mmol, 1.2 equiv.) and **S8**(26.0 mg, 0.058 mmol, 1.00 equiv.) were dissolved in DMF (600 uL) and DIPEA (25.0 uL, 0.144 mmol, 2.45 equiv.) was added. Then, HATU (22.2 mg, 0.058 mmol, 1.00 equiv.) was added in one portion to the orange solution. After 12h, LCMS showed full conversion to the desired NHFmoc intermediate. Piperidine (46.0 uL, 0.486 mmol, 8.00 equiv.) was added in one go to the clear dark orange reaction mixture and after 15 min, LCMS showed full conversion to the desired product. The reaction mixture was treated with AcOH (50 uL) and directly subjected to RP-HPLC purification (5-40% MeCN in water, containing 0.1% formic acid, t_R= 7.515 min, 360 nm). After evaporation of the solvent under reduced pressure, the desired product **S9** was obtained as clear orange oil in 25% yield (8.8 mg, 0.006 mmol).

Intermediated NHFmoc:

LCMS (5-100% MeCN in H_2O with 0.1% formic acid over 5 min) t_R = 3.603 min, 360 nm detection.

LRMS (ESI): calc. for for $C_{92}H_{145}N_7O_{28}^{2+}$ $[M+2H]^{2+}$: 898.5; found 898.2.

Product:

HRMS (ESI): calc. for $C_{77}H_{134}N_7O_{26}^{+}$ $[M+H]^{+}$: 1572.9373; found 1572.9301.

LCMS (5-100% MeCN in H_2O with 0.1% formic acid over 5 min) t_R = 2.777 min, 360 nm detection.

LRMS (ESI): calc. for for $C_{77}H_{133}N_7O_{26}Na_3^{3+}$ $[M+3Na]^{3+}$: 546.9; found 547.9.

^{1}H NMR (400 MHz, MeOD) δ 7.8 – 7.7 (m, 6H), 7.1 – 6.9 (m, 4H), 6.7 (d, *J* = 8.7 Hz, 2H), 3.9 (d, *J* = 6.3 Hz, 5H), 3.8 (t, *J* = 5.1 Hz, 2H), 3.7 – 3.6 (m, 96H), 3.4 – 3.3 (m, 2H), 3.2 – 3.2 (m, 4H), 3.0 (s, 4H), 2.9 – 2.8 (m, 2H), 2.7 (t, *J* = 5.9 Hz, 2H), 2.0 (t, *J* = 7.6 Hz, 2H).

^{13}C NMR (101 MHz, MeOD) δ 172.4, 153.9, 153.3, 150.6, 145.3, 141.6, 141.3, 126.2, 125.0, 123.8, 122.2, 121.2, 119.6, 113.0, 112.9, 71.5, 71.5, 71.5, 71.4, 71.4, 71.3, 71.3, 71.3, 71.2, 71.2, 71.2, 71.1, 71.0, 70.8, 68.2, 68.0, 57.0, 56.0, 54.1, 50.8, 42.1, 40.7, 38.7, 26.3.

IR (neat) 2867 (b), 1594 (m), 1541 (w), 1500 (m), 1452 (m), 1348 (m), 1300 (m), 1244 (m), 1098 (s), 949 (m), 850 (m) cm^{-1}.

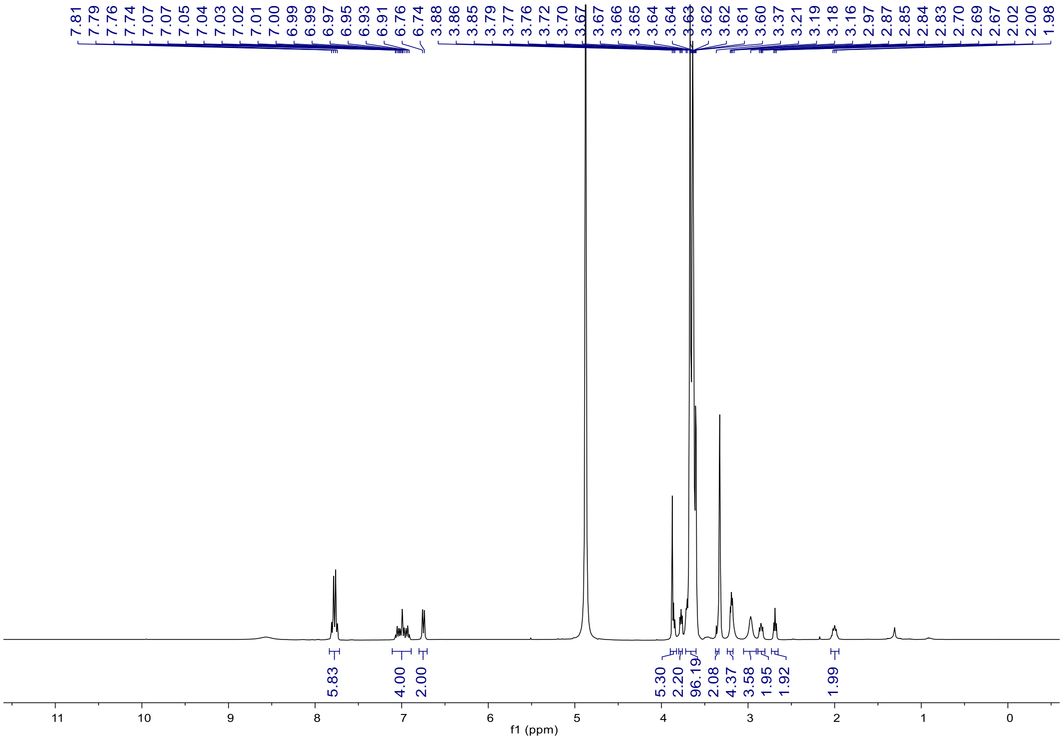
7.81 7.79 7.76 7.74 7.07 7.07 7.05 7.04 7.03 7.02 7.01 7.00 6.99 6.99 6.97 6.95 6.93 6.91 6.76 6.74 3.88 3.86 3.85 3.79 3.77 3.76 3.72 3.70 3.67 3.67 3.66 3.65 3.64 3.64 3.63 3.62 3.62 3.61 3.60 3.37 3.21 3.19 3.18 3.16 2.97 2.87 2.85 2.84 2.83 2.70 2.69 2.67 2.02 2.00 1.98
5.83 4.00 2.00 5.30 2.20 96.19 2.08 4.37 3.58 1.95 1.92 1.99
11 10 9 8 7 6 5 4 3 2 1 0
f1 (ppm)
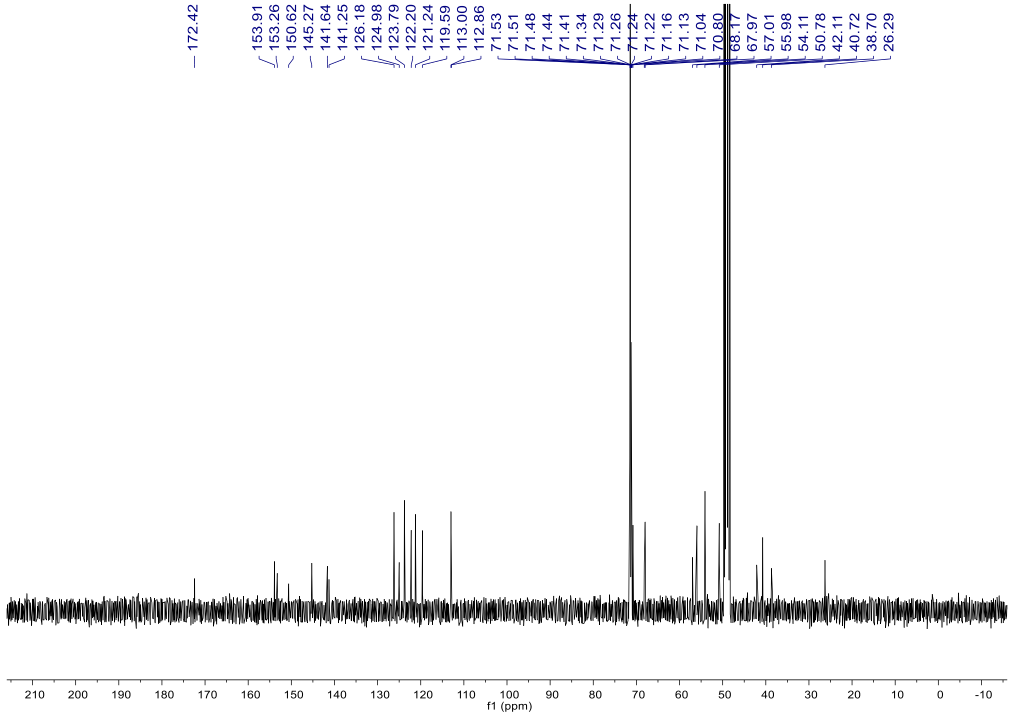
172.42
153.91 153.26 150.62 145.27 141.64 141.25 126.18 124.98 123.79 122.20 121.24 119.59 113.00 112.86 71.53 71.51 71.48 71.44 71.41 71.34 71.29 71.26 71.24 71.22 71.16 71.13 71.04 70.80 68.17 67.97 57.01 55.98 54.11 50.78 42.11 40.72 38.70 26.29
210 200 190 180 170 160 150 140 130 120 110 100 90 80 70 60 50 40 30 20 10 0 -10
f1 (ppm)

1.2.17 P-D2$_{block}$(C3)

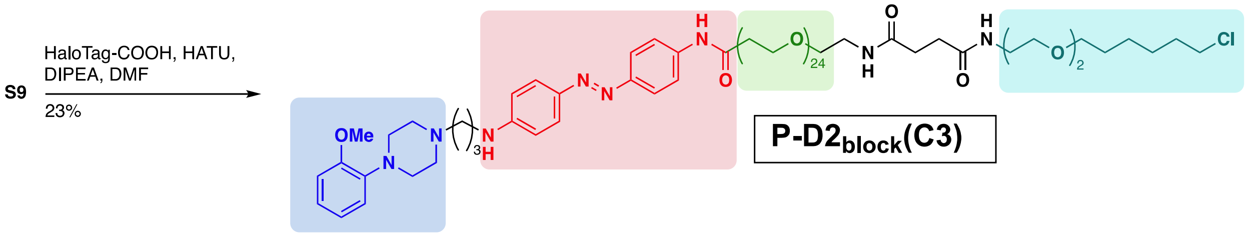

In a 4 mL vial, HaloTag-COOH (2.1 mg, 0.006 mmol, 1.00 equiv.) and **S9** (10.0 mg, 0.006 mmol, 1.00 equiv.) were dissolved in DMF (200 uL), and DIPEA (10 uL, 0.058 mmol, 9 equiv.) was added. The red solution was stirred for 1h. Full conversion was determined by LCMS analysis, and the mixture was diluted and subjected to RP-HPLC (semiprep, 7 min run, 15-60% MeCN in water, containing 0.1% formic acid, 9 mL/min, t_R = 5.348 min, 360 nm detection). After evaporation of the solvent under reduced pressure, the desired product **P-D2$_{block}$(C3)** was obtained as clear orange oil in 23% yield (2.8 mg, 1.35 umol).

HRMS (ESI): calc. for $C_{91}H_{157}ClN_8K_2O_{30}^{2+}$ $[M+2K]^{2+}$: 977.4979; found 977.4969.

LCMS (5-100% MeCN in H2O with 0.1% formic acid over 5 min) t_R = 3.464 min, 360 nm detection.

LRMS (ESI): calc. for for $C_{91}H_{160}ClN_8O_{30}^{3+}$ $[M+3H]^{3+}$: 626.7; found 626.8.

^{1}H NMR (400 MHz, MeOD) δ 7.8 – 7.7 (m, 6H), 7.1 – 6.9 (m, 4H), 6.8 – 6.7 (m, 2H), 3.9 – 3.8 (m, 5H), 3.6 – 3.5 (m, 108H), 3.4 (s, 2H), 3.2 – 3.0 (m, 4H), 2.9 (s, 4H), 2.8 (t, *J* = 7.7 Hz, 2H), 2.7 (t, *J* = 5.9 Hz, 2H), 2.5 (s, 4H), 2.0 – 1.9 (m, 2H), 1.8 – 1.7 (m, 2H), 1.6 – 1.6 (m, 2H), 1.5 – 1.4 (m, 4H).

^{13}C NMR (101 MHz, MeOD) δ 174.6, 172.4, 163.4, 162.7, 153.9, 153.3, 150.6, 145.3, 141.7, 141.3, 126.2, 124.9, 123.8, 122.2, 121.2, 119.6, 113.0, 112.9, 72.2, 71.6, 71.6, 71.5, 71.5, 71.3, 71.3, 71.2, 70.6, 68.2, 57.2, 56.0, 54.2, 51.0, 45.7, 42.2, 40.5, 40.4, 38.8, 33.8, 32.3, 30.5, 27.8, 26.5.

IR (neat) 3335 (b), 2868 (m), 1660 (w), 1590 (m), 1501 (m), 1348 (w), 1242 (w), 1137 (s), 1109 (s), 844 (w) cm^{-1}.

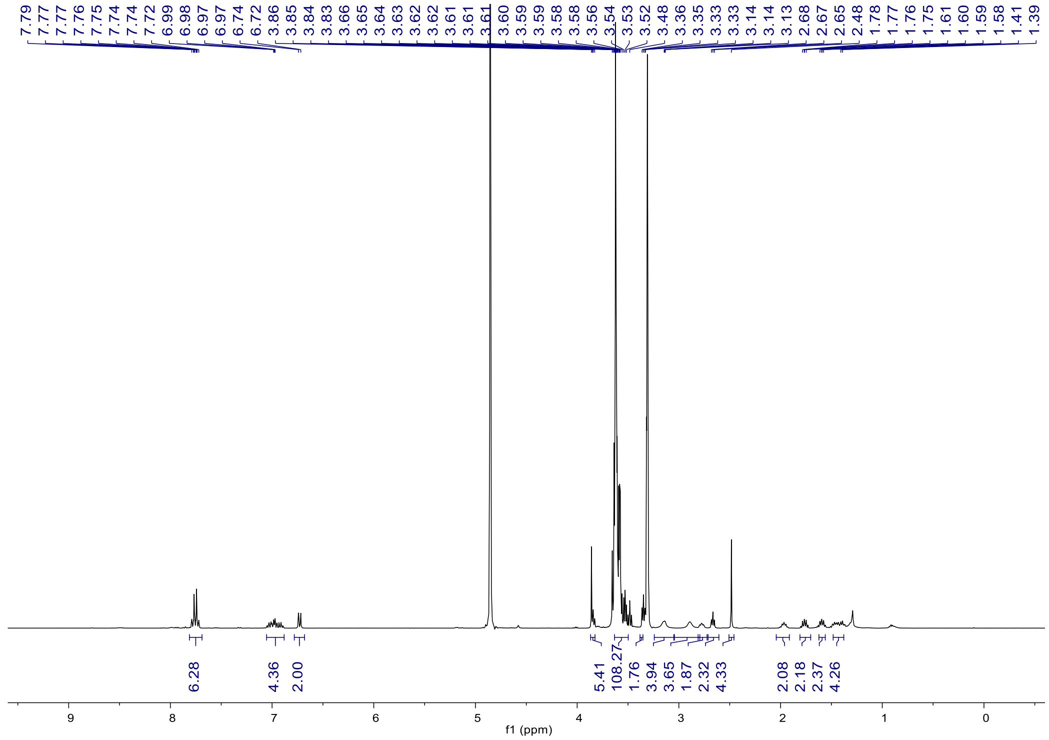
7.79 7.77 7.77 7.76 7.75 7.74 7.74 7.72 6.99 6.98 6.97 6.97 6.74 6.72 3.86 3.85 3.84 3.83 3.66 3.65 3.64 3.63 3.62 3.62 3.61 3.61 3.61 3.60 3.59 3.59 3.58 3.58 3.56 3.54 3.53 3.52 3.48 3.36 3.35 3.33 3.33 3.14 3.14 3.13 2.68 2.67 2.65 2.48 1.78 1.77 1.76 1.75 1.61 1.60 1.59 1.58 1.41 1.39
6.28 4.36 2.00 5.41 108.27 1.76 3.94 3.65 1.87 2.32 4.33 2.08 2.18 2.37 4.26
9 8 7 6 5 4 3 2 1 0
f1 (ppm)
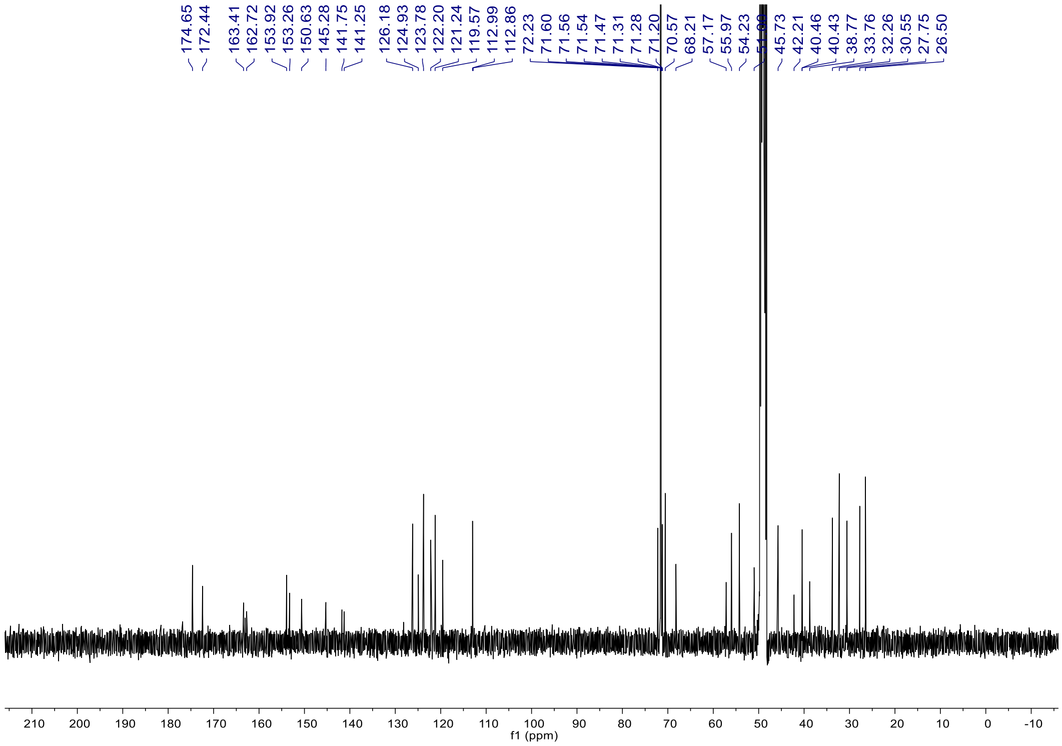
174.65 172.44 163.41 162.72 153.92 153.26 150.63 145.28 141.75 141.25 126.18 124.93 123.78 122.20 121.24 119.57 112.99 112.86 72.23 71.60 71.56 71.54 71.47 71.31 71.28 71.20 70.57 68.21 57.17 55.97 54.23 45.73 42.21 40.46 40.43 38.77 33.76 32.26 30.55 27.75 26.50
210 200 190 180 170 160 150 140 130 120 110 100 90 80 70 60 50 40 30 20 10 0 -10
f1 (ppm)

1.2.18 S11: C4-Methoxyphenylpiperazine Chloride

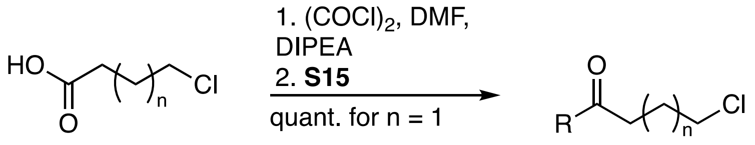

S10 (n = 1) **S11** (n = 1)

2 has previously been used (*J. Med. Chem.* **1997**, *40*, 2653–2656).

In a 5 mL rbf, 4-chlorobutyric acid (**S10**) (62 uL, 0.624 mmol, 1.20 equiv.) and DMF (1 drop) were dissolved in THF (2.6 mL). The mixture was cooled in an ice-water bath, and then oxalyl chloride (62 uL, 0.728 mmol, 1.40 equiv.) was added dropwise under evolution of bubbles. The mixture was warmed to room temperature and stirred for another 30 min. The solvent was removed under reduced pressure and the yellow oily residue taken up in THF (1 mL). In a separate 5 mL rbf, 1-(2-methoxyphenyl)piperazine (100 mg, 0.520 mmol, 1.00 equiv.), DIPEA (181 uL, 1.04 mmol, 2.00 equiv.), and DMAP (1 grain) were dissolved in THF (2.6 mL), and cooled in an ice-water bath. The acyl chloride solution was added dropwise under evolution of fumes. After 30 seconds, a white precipitate occurred. The reaction was stirred at room temperature for 10 min, then the solvent removed under reduced pressure. The crude white solid was subjected to FCC (24 g silica, DCM-5% MeOH in DCM), to yield the product **S11** as light yellow oil in quant. yield (158 mg, 0.532 mmol).

R_f = 0.65 (5% MeOH in DCM; UV detection).

HRMS (ESI): calc. for $C_{15}H_{22}ClN_2O_2^+$ [M+H]$^+$: 297.1364; found 297.1362.

LCMS (5-100% MeCN in H_2O with 0.1% formic acid over 5 min) t_R = 3.139 min, 254 nm detection.

LRMS (ESI): calc. for $C_{15}H_{22}ClN_2O_2^+$ [M+H]$^+$: 297.1; found 297.1.

^{1}H NMR (400 MHz, $CDCl_3$) δ 7.1 – 7.0 (m, 1H), 7.0 – 6.8 (m, 3H), 3.9 (s, 3H), 3.8 (t, *J* = 5.2 Hz, 2H), 3.7 – 3.6 (m, 4H), 3.1 – 3.0 (m, 4H), 2.6 (t, *J* = 7.1 Hz, 2H), 2.2 – 2.1 (m, 2H).

^{13}C NMR (101 MHz, $CDCl_3$) δ 170.3, 152.4, 140.8, 123.8, 121.2, 118.6, 111.5, 55.6, 51.2, 50.8, 45.1, 42.0, 29.9, 28.1.

IR (neat) 3404 (b), 2956 (w), 2832 (w), 2724 (w), 2492 (w), 1764 (m), 1622 (m), 1593 (m), 1499 (s), 1452 (m), 1381 (w), 1239 (s), 1177 (s), 1151 (m), 1115 (m), 1057 (s), 925 (m), 749 (m) cm^{-1}.

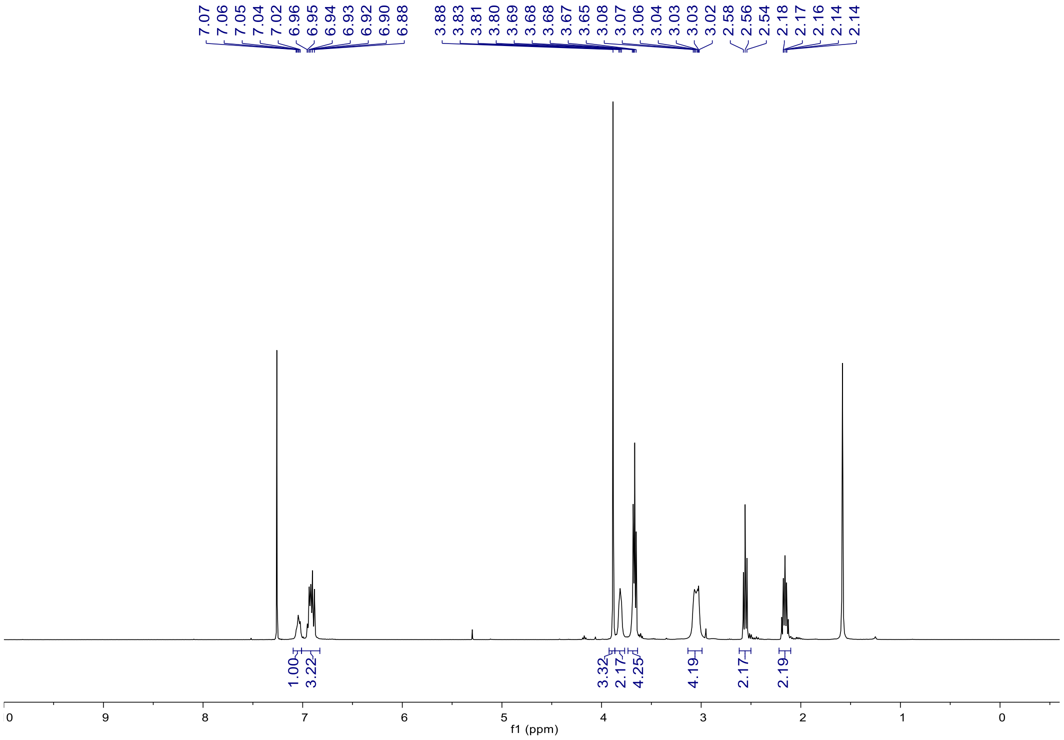
7.07
7.06
7.05
7.04
7.02
6.96
6.95
6.94
6.93
6.92
6.90
6.88
3.88
3.83
3.81
3.80
3.69
3.68
3.68
3.67
3.65
3.08
3.07
3.06
3.04
3.03
3.03
3.02
2.58
2.56
2.54
2.18
2.17
2.16
2.14
2.14
1.00
3.22
3.32
2.17
4.25
4.19
2.17
2.19
f1 (ppm)

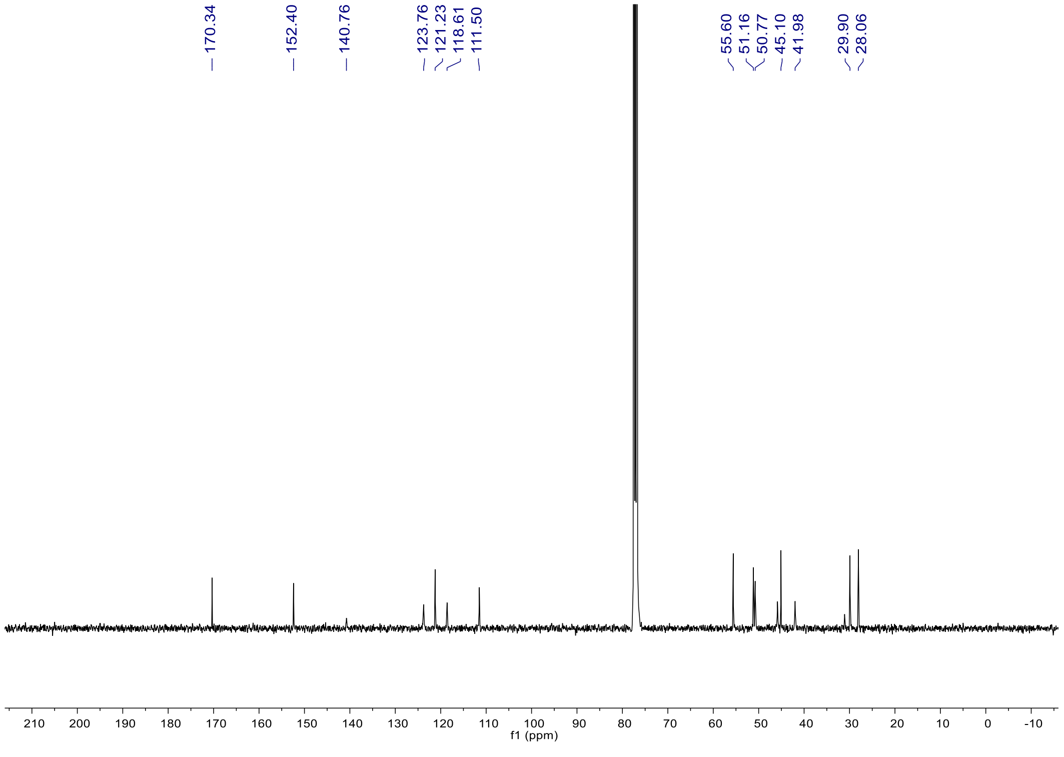
170.34
152.40
140.76
123.76
121.23
118.61
111.50
55.60
51.16
50.77
45.10
41.98
29.90
28.06
f1 (ppm)

1.2.19 S12: C4-Methoxyphenylpiperazine Amide Azo

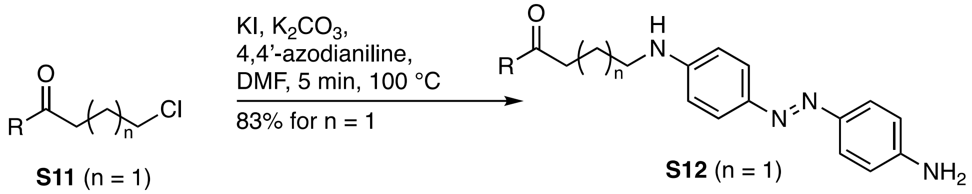

In a 20 mL scintillation vial, **S11** (30.0 mg, 0.101 mmol, 1.00 equiv.), KI (50.3 mg, 0.303 mmol, 3.00 equiv.) and 4,4'-azodianiline (21.5 mg, 0.101 mmol, 1.00 equiv.) were dissolved in DMF (0.5 mL), and the orange reaction mixture heated to 60 °C. After 3h, the dark green reaction was judged complete by LCMS analysis. The solvent was removed under reduced pressure at 50 °C. The residue was taken up in DCM and washed with LiCl (10 %, aq., 5x). The organic phase was dried over Na_2SO_4, filtered, and concentrated under reduced pressure. The red residue was subjected to FCC (4g silica, 0 -> 5% MeOH in DCM), to yield the product **S12** as dark orange oil in 83% yield (39.4 mg, 0.083 mmol).

$\mathbf{R}_{\boldsymbol{f}}$ = 0.23 (5% MeOH in DCM; red spot).

HRMS (ESI): calc. for $C_{27}H_{33}N_6O_2^+$ $[M+H]^+$: 473.2660; found 473.2659.

LCMS (5-100% MeCN in H_2O with 0.1% formic acid over 5 min) t_R = 3.463 min, 360 nm detection.

LRMS (ESI): calc. for $C_{27}H_{33}N_6O_2^+$ $[M+H]^+$: 473.3; found 473.3.

^{1}H NMR (400 MHz, $CDCl_3$) δ 7.8 – 7.7 (m, 4H), 7.1 – 7.0 (m, 1H), 7.0 – 6.8 (m, 3H), 6.8 – 6.6 (m, 4H), 3.9 (s, 3H), 3.8 (t, *J* = 5.1 Hz, 2H), 3.6 (t, *J* = 4.9 Hz, 2H), 3.3 (t, *J* = 6.5 Hz, 2H), 3.0 – 2.9 (m, 4H), 2.5 (t, *J* = 6.8 Hz, 2H), 2.1 – 1.9 (m, 2H).

^{13}C NMR (101 MHz, $CDCl_3$) δ 171.1, 152.3, 150.3, 148.4, 145.9, 144.7, 140.6, 124.6, 124.2, 123.6, 121.1, 118.5, 114.8, 112.2, 111.4, 55.5, 50.9, 50.6, 45.8, 43.5, 42.0, 30.9, 24.5.

IR (neat) 3346 (b), 2923 (w), 1595 (s), 1500 (m), 1445 (w), 1336 (w), 1301 (w), 1241 (m), 1149 (m), 1027 (m), 836 (w), 752 (w) cm^{-1}.

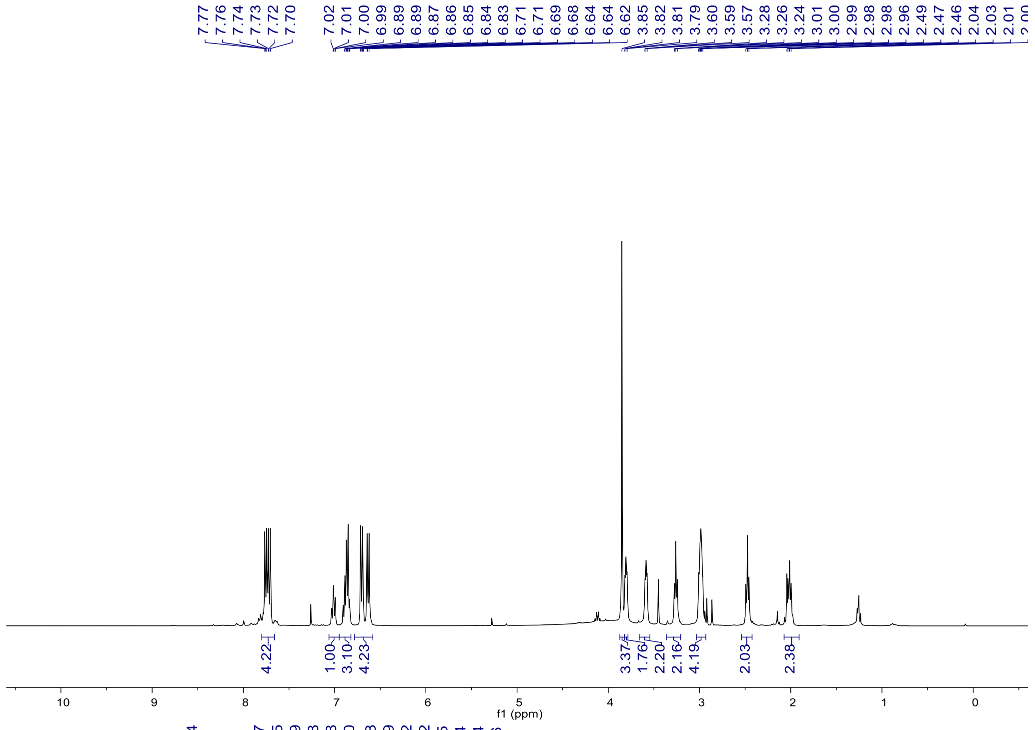
7.77
7.76
7.74
7.73
7.72
7.70
7.02
7.01
7.00
6.99
6.89
6.89
6.87
6.86
6.85
6.84
6.83
6.71
6.69
6.68
6.64
6.64
6.62
3.85
3.82
3.81
3.79
3.60
3.59
3.57
3.28
3.26
3.24
3.01
3.00
2.99
2.98
2.98
2.96
2.49
2.47
2.46
2.04
2.03
2.01
4.22
1.00
3.10
4.23
3.37
1.76
2.20
2.16
4.19
2.03
2.38
f1 (ppm)

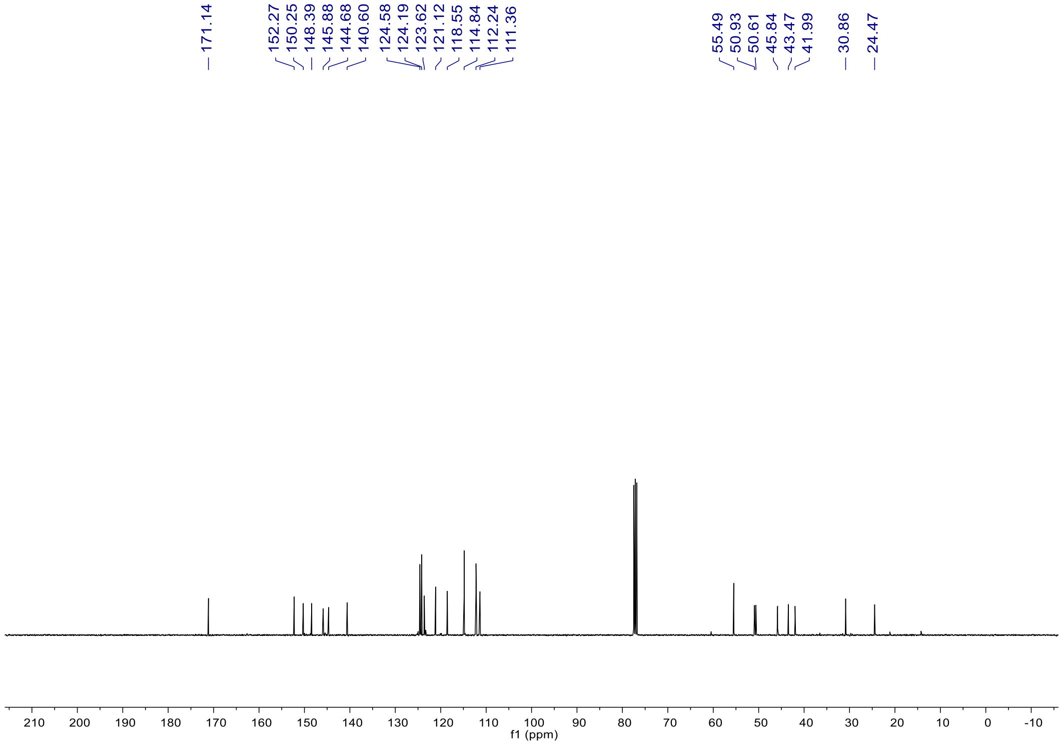
171.14
152.27
150.25
148.39
145.88
144.68
140.60
124.58
124.19
123.62
121.12
118.55
114.84
112.24
111.36
55.49
50.93
50.61
45.84
43.47
41.99
30.86
24.47
f1 (ppm)

1.2.20 S13: C4-Methoxyphenylpiperazine Azo

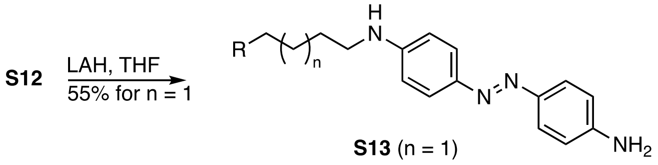

In a 25 mL rbf, LAH (1M in THF, 567 uL, 0.567 mmol, 4.00 equiv.) was cooled in an ice-water bath. **S12** (67.0 mg, 0.142 mmol, 1.00 equiv., as solution in 2 mL THF) was added dropwise. The dark red solution was warmed to room temperature and stirred for another 30 min. Then, the solution was cooled in an ice-water bath, and NaOH (5 mL, 2M) was added slowly under evolution of bubbles. Then, DCM was added, and the phases separated. The organic phase was dried over Na_2SO_4, filtered, and concentrated under reduced pressure. The red oil was subjected to FCC (4g silica, 0 -> 5% MeOH in DCM) to yield the desired product **S13** in 55% yield (36 mg, 0.078 mmol).

R_f = 0.21 (5% MeOH in DCM; red spot).

HRMS (ESI): calc. for $C_{27}H_{36}N_6O^{2+}$ [M+2H] $^{2+}$: 230.1470; found 230.1477.

LCMS (5-100% MeCN in H_2O with 0.1% formic acid over 5 min) t_R = 2.797 min, 360 nm detection.

LRMS (ESI): calc. for $C_{27}H_{35}N_6O^+$ [M+H] $^+$: 459.3; found 459.3.

^{1}H NMR (400 MHz, $CDCl_3$) δ 7.9 – 7.6 (m, 4H), 7.1 – 6.8 (m, 4H), 6.8 – 6.6 (m, 4H), 3.9 (s, 3H), 3.3 – 3.2 (m, 2H), 3.1 (s, 4H), 2.7 (s, 4H), 2.5 (t, J = 6.9 Hz, 2H), 1.8 – 1.6 (m, 4H).

^{13}C NMR (101 MHz, $CDCl_3$) δ 152.4, 150.4, 148.3, 146.1, 144.8, 141.3, 124.6, 124.3, 123.1, 121.1, 118.4, 114.9, 112.2, 111.3, 58.1, 55.5, 53.5, 50.7, 43.7, 27.3, 24.6.

IR (neat) 3340 (b), 2932 (w), 2829 (w), 1595 (s), 1500 (m), 1450 (w), 1336 (w), 1299 (w), 1341 (m), 1147 (m), 1025 (w), 835 (w), 751 (w) cm^{-1}.

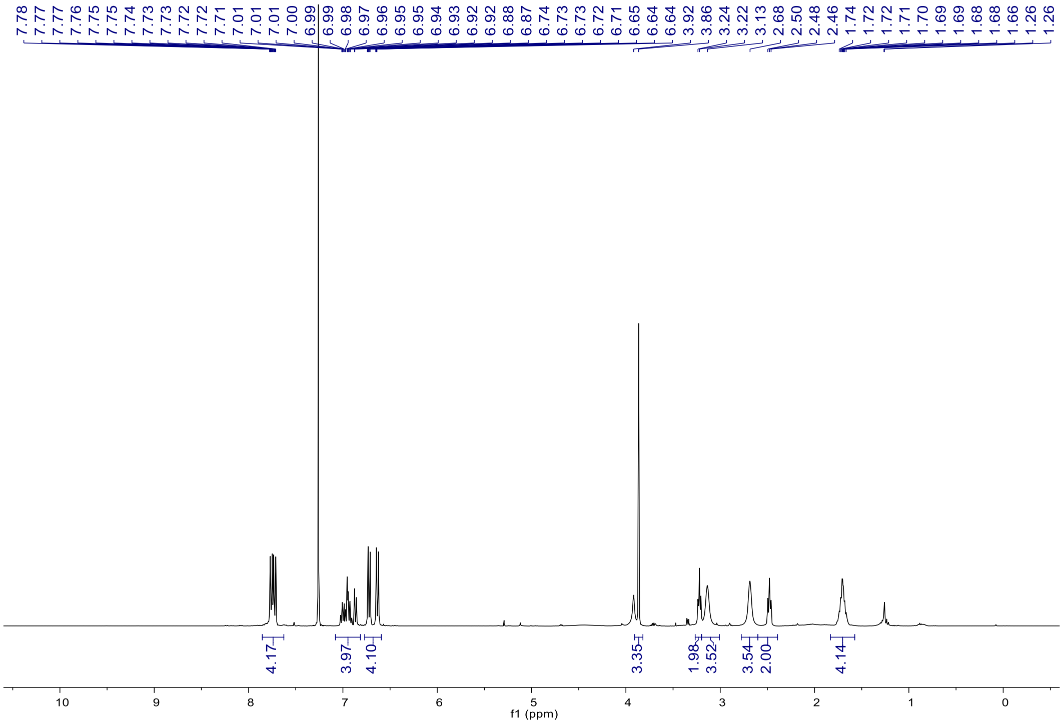
7.78 7.77 7.77 7.76 7.75 7.74 7.73 7.73 7.72 7.72 7.71 7.01 7.01 7.00 6.99 6.99 6.98 6.97 6.96 6.95 6.94 6.93 6.92 6.92 6.88 6.87 6.74 6.73 6.73 6.72 6.71 6.65 6.64 6.64 3.92 3.86 3.24 3.22 3.13 2.68 2.50 2.48 2.46 1.74 1.72 1.72 1.71 1.70 1.69 1.69 1.68 1.68 1.66 1.26 1.26
4.17 3.97 4.10 3.35 1.98 3.52 3.54 2.00 4.14
10 9 8 7 6 5 4 3 2 1 0
f1 (ppm)

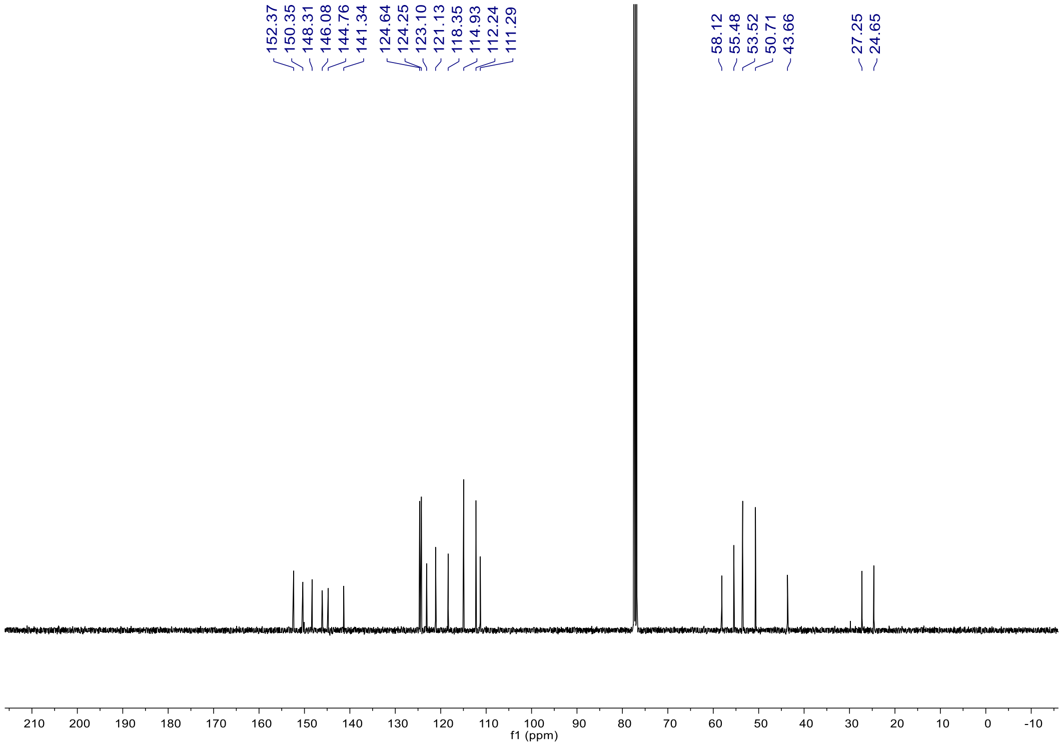
152.37 150.35 148.31 146.08 144.76 141.34 124.64 124.25 123.10 121.13 118.35 114.93 112.24 111.29
58.12 55.48 53.52 50.71 43.66
27.25 24.65
210 200 190 180 170 160 150 140 130 120 110 100 90 80 70 60 50 40 30 20 10 0 -10
f1 (ppm)

1.2.21 S14: C4-Methoxyphenylpiperazine Azo PEG

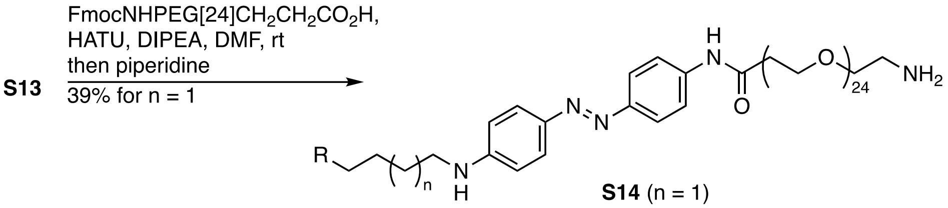

S14 (n = 1)

In a 20 mL vial, **S13** (36.0 mg, 0.078 mmol, 1.00 equiv.) and FmocHNPEG[24]CH_2CH_2COOH (107 mg, 0.078 mmol, 1.00 equiv.) were dissolved in DMF (1.3 mL), and DIPEA (68 uL, 0.392 mmol, 5.00 equiv.) was added. Then, HATU (29.8 mg, 0.078 mmol, 1.00 equiv.) was added in one portion. After 10h, LCMS showed full conversion to the desired product. Piperidine (155 uL) was added. After LCMS confirmed complete deprotection, the crude reaction solution was diluted with MeCN and treated with AcOH (200 uL), and subjected to RP-HPLC purification (15-42% MeCN in water containing 0.1% FA, 80 mL/min, 6 min runtime, prep column, 360 nm detection, t_R = 4.263 min). After evaporation of the solvent under reduced pressure, the desired product **S14** was obtained in 39% yield as orange oil (48.6 mg, 0.031 mmol).

Fmoc Intermediate:

LCMS (5-100% MeCN in H_2O with 0.1% formic acid over 5 min) t_R = 3.745 min, 360 nm detection.

LRMS (ESI): calc. for $C_{93}H_{147}N_7O_{28}^{2+}$ $[M+2H]^{2+}$: 905.5; found 905.5.

Product:

HRMS (ESI): calc. for $C_{78}H_{137}N_7O_{26}^{2+}$ $[M+2H]^{2+}$: 793.9801; found 793.9812.

LCMS (5-100% MeCN in H_2O with 0.1% formic acid over 5 min) t_R = 3.058 min, 360 nm detection.

LRMS (ESI): calc. for $C_{78}H_{136}N_7O_{26}Na^{2+}$ $[M+Na+H]^{2+}$: 804.9; found 805.0.

^{1}H NMR (400 MHz, MeOD) δ 7.8 – 7.7 (m, 6H), 7.1 – 6.9 (m, 4H), 6.7 (d, *J* = 8.6 Hz, 2H), 3.9 (s, 5H), 3.8 (t, *J* = 5.1 Hz, 2H), 3.7 – 3.6 (m, 90H), 3.4 (s, 8H), 3.3 (s, 4H), 3.2 (t, *J* = 5.2 Hz, 4H), 2.7 (t, *J* = 5.9 Hz, 2H), 1.9 (s, 2H), 1.7 (t, *J* = 7.3 Hz, 2H).

^{13}C NMR (101 MHz, MeOD) δ 172.4, 153.9, 153.3, 150.6, 145.2, 141.3, 140.7, 126.2, 125.5, 123.8, 122.2, 121.2, 119.8, 113.0, 112.9, 71.5, 71.4, 71.4, 71.4, 71.4, 71.3, 71.3, 71.2, 71.2, 71.2, 71.1, 71.1, 71.0, 70.8, 68.1, 67.9, 57.8, 56.0, 53.4, 43.4, 40.7, 38.7, 27.3, 22.8.

IR (neat) 3432 (b), 2871 (m), 1596 (s), 1539 (m), 1501 (w), 1454 1347 (m), 1301 (w), 1246 (w), 1100 (s), 949 (w), 845 (m), 755 (w) cm^{-1}.

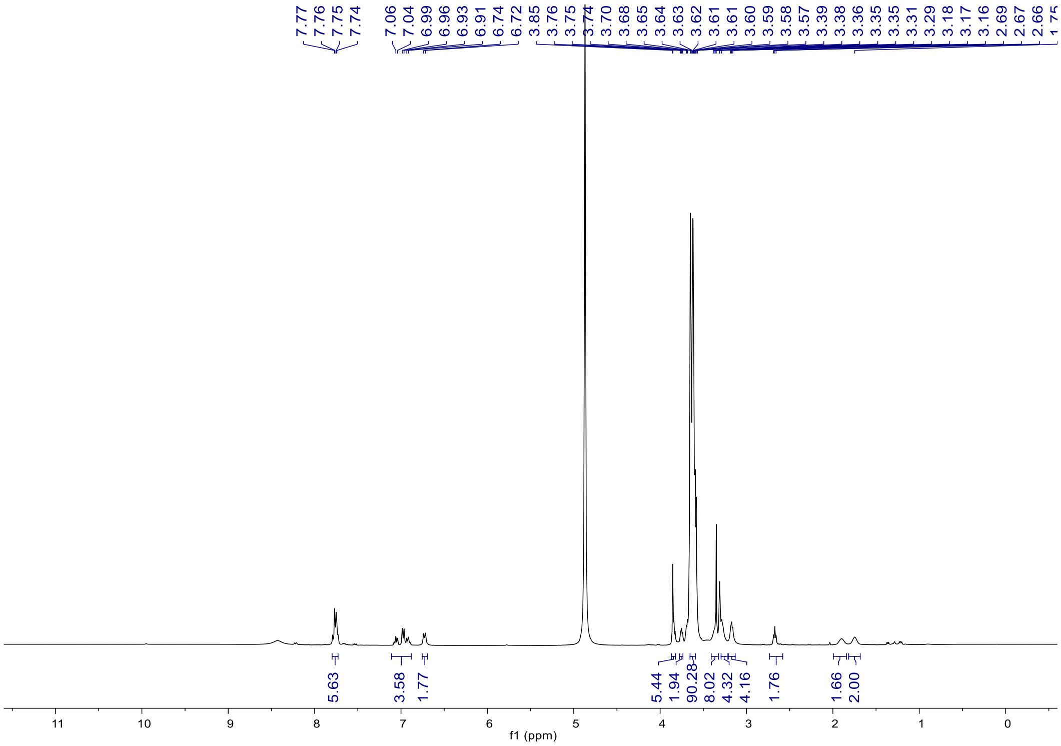

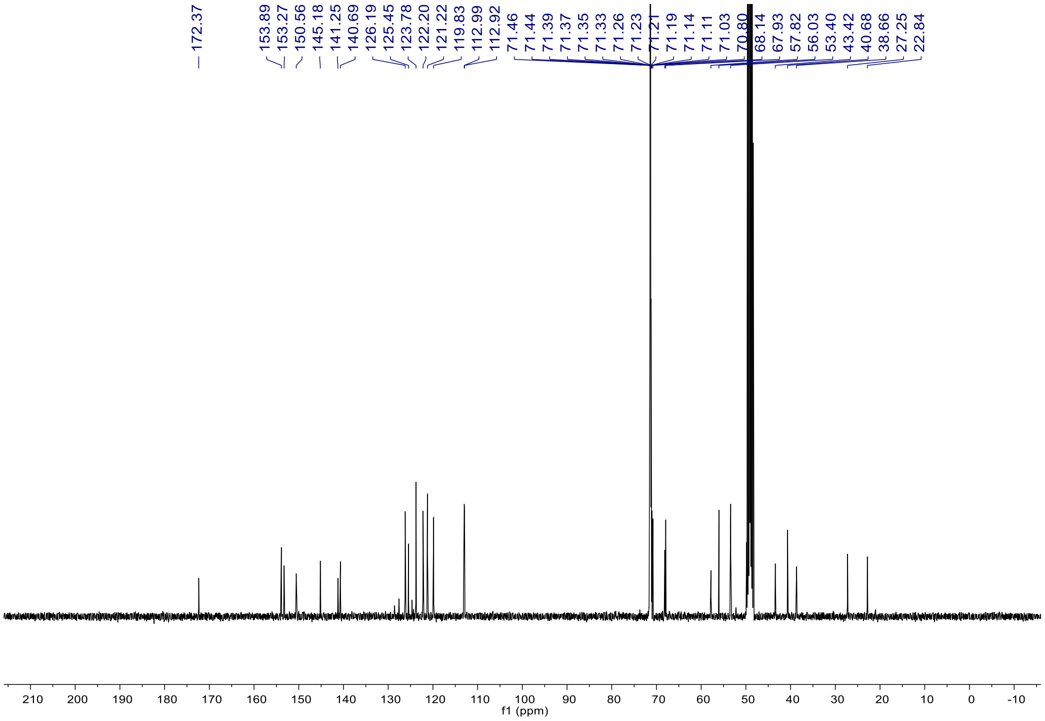

1.2.22 P-D2$_{block}$(C4)

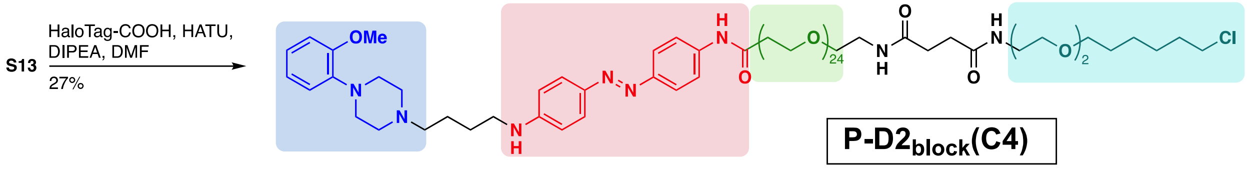

In a 4 mL vial, **S13** (10.0 mg, 6.3 umol, 1.00 equiv.) and HaloTag-COOH (2.0 mg, 6.3 umol, 1.00 equiv.) were dissolved in DMF (100 uL), and DIPEA (10 uL, 57 umol, 9 equiv.) was added. Then, HATU (2.4 mg, 6.4 umol, 1.00 equiv.) was added in one portion. After 10h, LCMS showed full conversion to the desired product. The crude reaction solution was diluted with MeCN and treated with AcOH (20 uL), and subjected to RP-HPLC purification (15-60% MeCN in water containing 0.1% FA, 9 mL/min, 7 min runtime, semiprep column, 360 nm detection, t_R = 5.410 min). After evaporation of the solvent under reduced pressure, the desired product **P-D2$_{block}$(C4)** was obtained in 27% yielded as orange oil (3.2 mg, 1.7 umol).

HRMS (ESI): calc. for $C_{92}H_{161}ClN_8O_{30}^{2+}$ $[M+2H]^{2+}$: 946.5498; found 946.5481.

LCMS (5-100% MeCN in H_2O with 0.1% formic acid over 5 min) t_R = 3.457min, 360 nm detection.

LRMS (ESI): calc. for $C_{92}H_{159}ClN_8O_{30}Na_2^{2+}$ $[M+2Na]^{2+}$: 969.0; found 969.2.

^{1}H NMR (600 MHz, MeOD) δ 7.9 – 7.7 (m, 6H), 7.1 – 6.9 (m, 4H), 6.8 – 6.6 (m, 2H), 3.9 (s, 5H), 3.6 – 3.5 (m, 112H), 3.3 (s, 2H), 3.1 (s, 4H), 2.8 (s, 4H), 2.7 (t, *J* = 5.9 Hz, 2H), 2.6 (t, *J* = 7.0 Hz, 2H), 2.5 (s, 4H), 1.8 – 1.7 (m, 6H), 1.6 (d, *J* = 6.8 Hz, 2H), 1.5 – 1.5 (m, 2H), 1.4 – 1.4 (m, 2H).

^{13}C NMR (151 MHz, MeOD) δ 174.7, 174.7, 172.5, 153.9, 153.5, 150.6, 145.1, 141.9, 141.2, 126.2, 126.2, 124.9, 123.7, 122.2, 121.2, 119.5, 112.9, 112.8, 72.2, 72.2, 71.6, 71.6, 71.5, 71.5, 71.5, 71.5, 71.5, 71.4, 71.4, 71.3, 71.3, 71.3, 71.3, 71.2, 71.2, 71.2, 71.2, 70.6, 70.6, 70.6, 70.6, 68.2, 59.2, 56.0, 54.2, 51.2, 45.7, 45.7, 43.9, 40.4, 40.4, 38.7, 33.7, 32.2, 30.5, 28.0, 27.7, 26.5, 24.7.

IR (neat) 3348 (b), 2868 (m), 1652 (w), 1599 (s), 1540 (m)., 1454 (w), 1348 (w), 1300 (w), 1244 (m), 1104 (s), 949 (w), 845 (w) cm^{-1}.

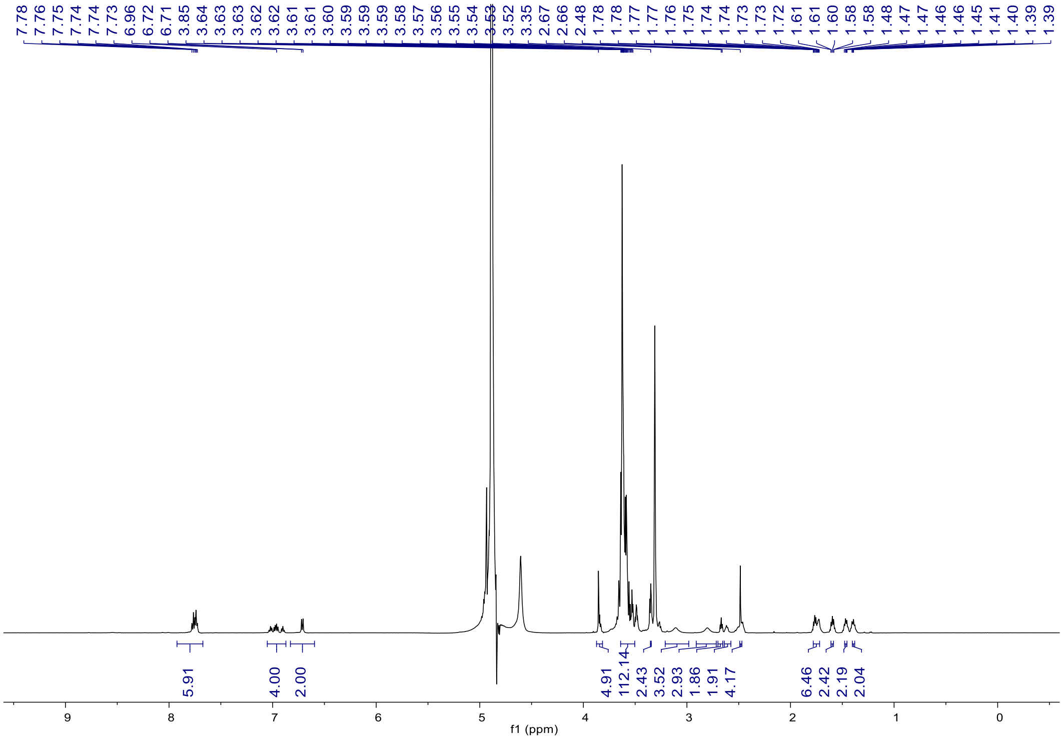

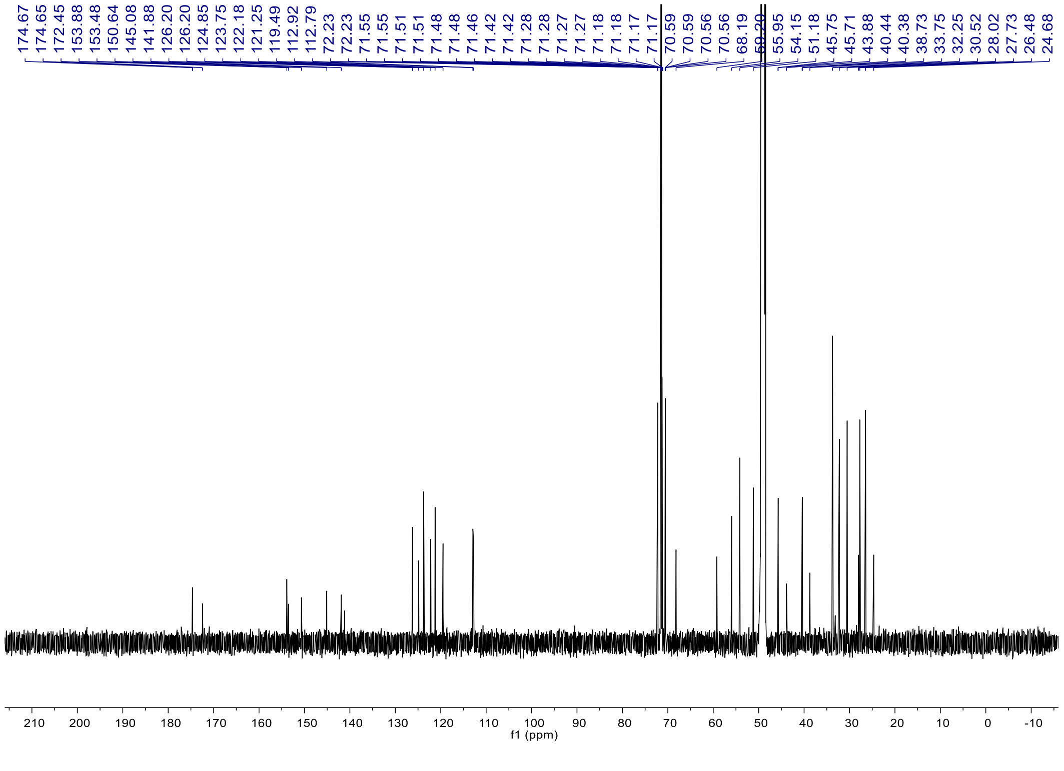

1.2.23 P-D2$_{block}$(C4,2x)

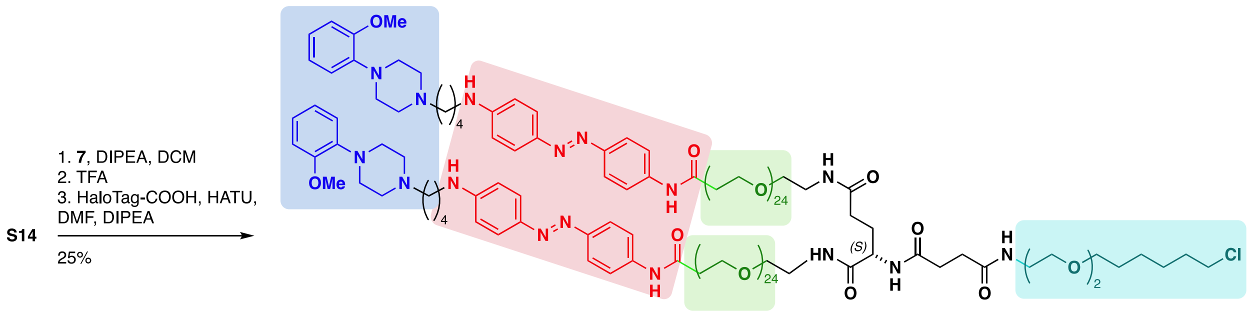

P-D2$_{block}$(C4)-2X

NHBoc-L-Glu-Osu (**7**) was prepared according to: Biomacromolecules, **2015** *16*, 3491-3498 and matched spectroiscopic data.

In a 20 mL vial, **S14** (12.0 mg, 17.7 mmol, 2.20 equiv.) and NHBoc-L-Glu-OSu (1.5 mg, 3.4 umol, 1.00 equiv.) were dissolved in DCM (35 uL), and DIPEA (3 uL, 13 umol, 4 equiv.) was added. After 10 min, LCMS showed full conversion to the desired dimer. The solvent was removed under reduced pressure, then TFA (1 mL) was added to the dark red oil, to form a magenta solution. After 5 min, TFA was removed under a gentle stream of nitrogen. This procedure was repeated twice until LCMS showed full conversion to the free amine. Then, HaloTag-COOH (1.1 mg, 3.4 umol, 1.0 equiv., as solution in 100 uL DMF) and DIPEA (12 uL, 68 umol, 20 equiv.) were added, forming a dark orange solution. HATU (1.3 mg, 3.4 umol, 1.0 equiv.) was added in one portion. After 12h, LCMS showed full conversion of the starting material was detected and the sample subjected to RP-HPLC purification (10-40% MeCN in water containing 0.1% FA, 9 mL/min, 10 min runtime, semiprep column, 360 nm detection, t_R = 8.565 min). After evaporation of the solvent under reduced pressure, the desired product **P-D2$_{block}$(C4)-2X** was obtained in 25% yield as orange oil (3.0 mg, 0.84 umol).

Intermediate NHBoc Dimer

LCMS (5-100% MeCN in H_2O with 0.1% formic acid over 5 min) t_R = 3.362 min, 360 nm detection.

LRMS (ESI): calc. for $C_{166}H_{287}N_{15}O_{56}^{4+}$ $[M+4H]^{4+}$: 847.2; found 847.2.

Intermediate NH2 Dimer

LCMS (5-100% MeCN in H_2O with 0.1% formic acid over 5 min) t_R = 3.123 min, 360 nm detection.

LRMS (ESI): calc. for $C_{161}H_{280}N_{15}O_{54}^{5+}$ $[M+5H]^{5+}$: 657.8; found 657.9.

Product

HRMS (ESI): calc. for $C_{175}H_{304}ClN_{16}O_{58}^{5+}$ $[M+5H]^{5+}$: 718.6198; found 718.6173

LCMS (5-100% MeCN in H_2O with 0.1% formic acid over 5 min) t_R = 3.418 min, 360 nm detection.

LRMS (ESI): calc. for $C_{175}H_{305}ClN_{16}O_{58}^{6+}$ $[M+6H]^{6+}$: 599.3; found 599.3.

^{1}H NMR (400 MHz, MeOD) δ 7.8 – 7.7 (m, 12H), 7.1 – 6.9 (m, 8H), 6.7 (d, *J* = 8.7 Hz, 4H), 4.3 (dd, *J* = 9.1, 5.0 Hz, 1H), 3.9 – 3.8 (m, 12H), 3.7 – 3.5 (m, 211H), 3.4 (d, *J* = 5.8 Hz, 7H), 3.3 – 3.1 (m, 4H), 2.7 (t, *J* = 5.9 Hz, 4H), 2.6 – 2.4 (m, 6H), 2.3 (t, *J* = 7.5 Hz, 2H), 2.2 – 2.1 (m, 1H), 2.0 – 1.9 (m, 5H), 1.8 – 1.7 (m, 6H), 1.7 – 1.6 (m, 3H), 1.5 – 1.4 (m, 6H).

^{13}C NMR (101 MHz, MeOD) δ 173.6, 173.5, 173.2, 172.5, 171.1, 152.5, 151.8, 149.2, 143.9, 139.9, 139.3, 124.8, 124.1, 122.4, 120.8, 119.9, 118.5, 111.6, 111.6, 70.8, 70.1, 70.0, 69.9, 69.8, 69.1, 69.0, 66.8, 56.6, 54.6, 52.2, 44.4, 44.3, 42.1, 39.0, 39.0, 37.4, 32.3, 31.8, 30.7, 30.5, 29.1, 29.1, 26.3, 25.9, 25.1, 21.7.

IR (neat) 3321 (b), 872 (m), 1656 (w), 1599 (m), 1537 (m), 1501 (w), 1450 (w), 1349 (w), 1299 (w), 1242 (m), 1104 (s), 949 (m), 846 (m) cm^{-1}.

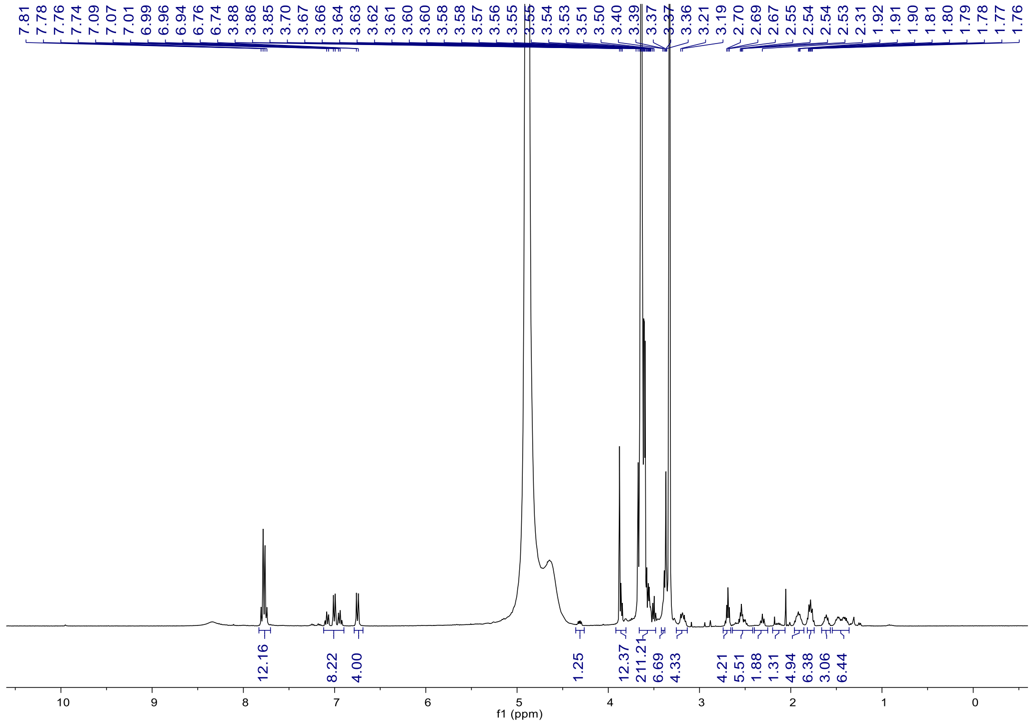

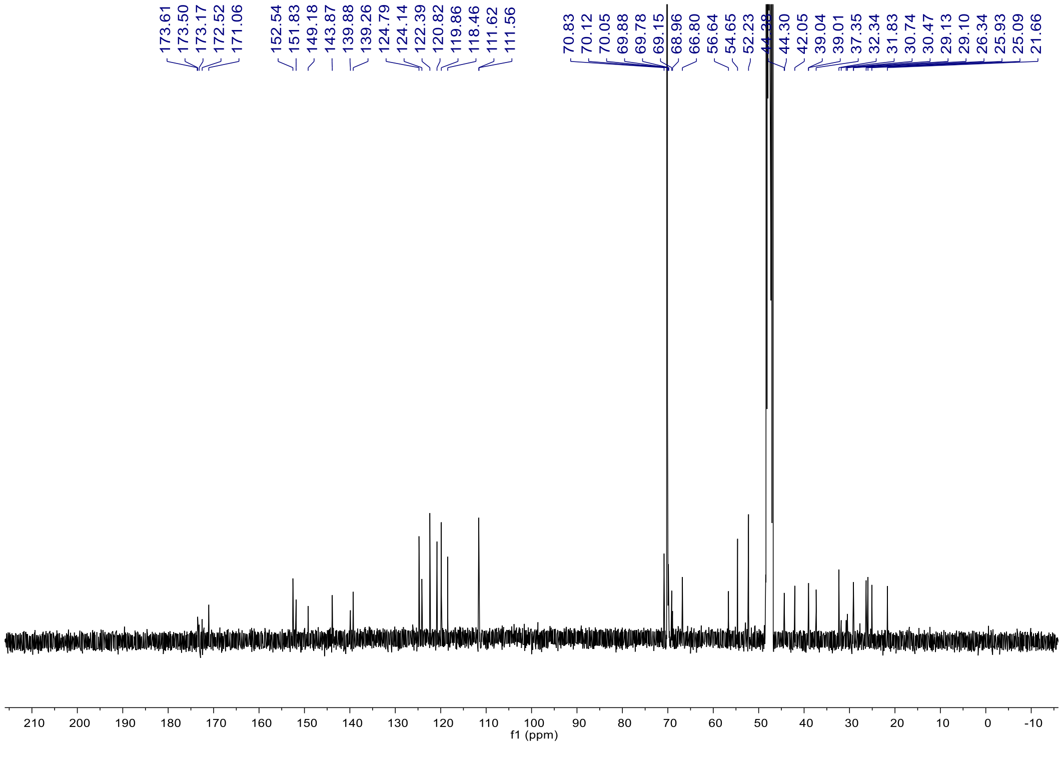

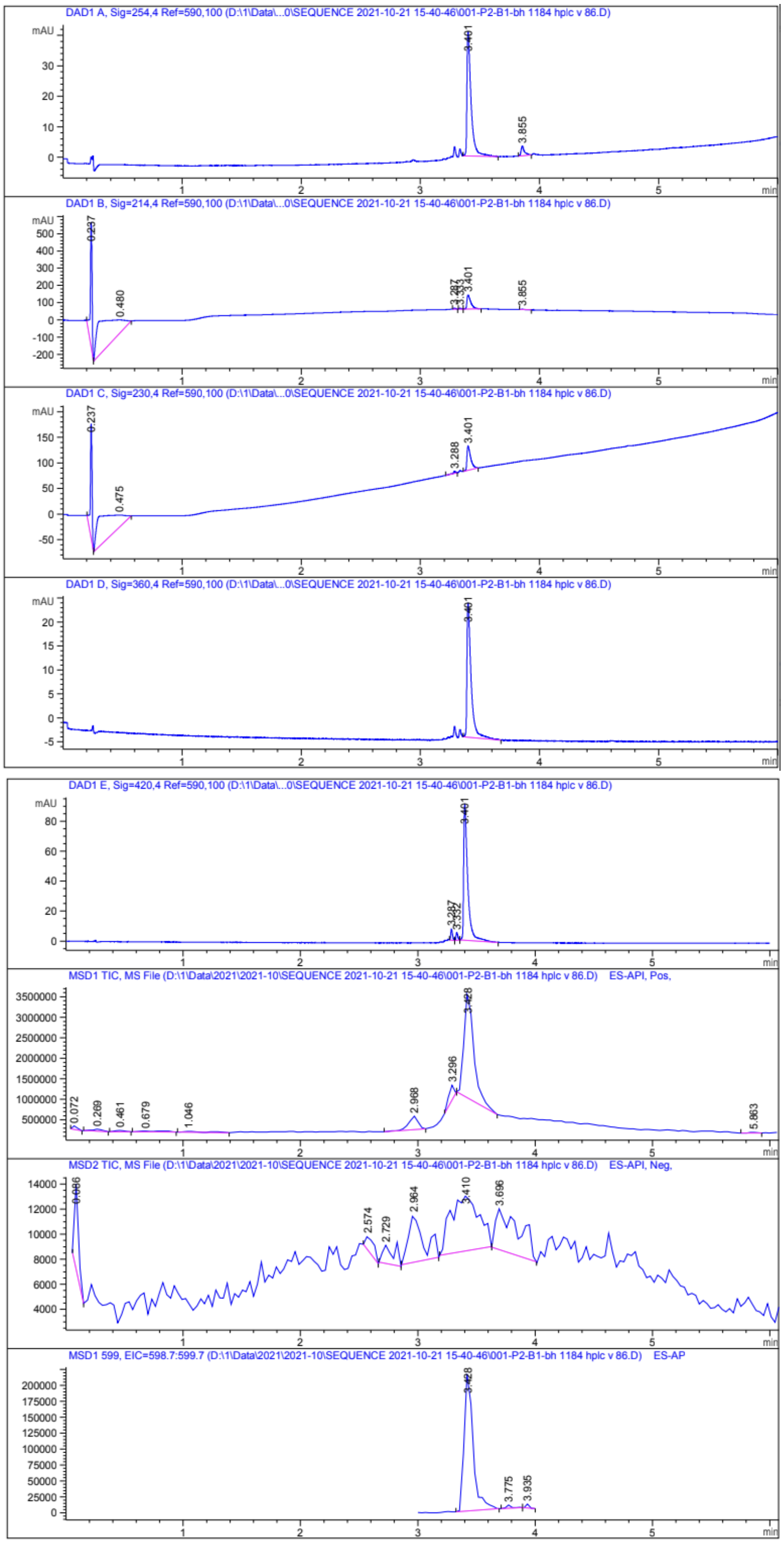
DAD1 A, Sig=254,4 Ref=590,100 (D:\1\Data\...0\SEQUENCE 2021-10-21 15-40-46\001-P2-B1-bh 1184 hplc v 86.D)
DAD1 B, Sig=214,4 Ref=590,100 (D:\1\Data\...0\SEQUENCE 2021-10-21 15-40-46\001-P2-B1-bh 1184 hplc v 86.D)
DAD1 C, Sig=230,4 Ref=590,100 (D:\1\Data\...0\SEQUENCE 2021-10-21 15-40-46\001-P2-B1-bh 1184 hplc v 86.D)
DAD1 D, Sig=360,4 Ref=590,100 (D:\1\Data\...0\SEQUENCE 2021-10-21 15-40-46\001-P2-B1-bh 1184 hplc v 86.D)
DAD1 E, Sig=420,4 Ref=590,100 (D:\1\Data\...0\SEQUENCE 2021-10-21 15-40-46\001-P2-B1-bh 1184 hplc v 86.D)
MSD1 TIC, MS File (D:\1\Data\2021\2021-10\SEQUENCE 2021-10-21 15-40-46\001-P2-B1-bh 1184 hplc v 86.D) ES-API, Pos,
MSD2 TIC, MS File (D:\1\Data\2021\2021-10\SEQUENCE 2021-10-21 15-40-46\001-P2-B1-bh 1184 hplc v 86.D) ES-API, Neg,
MSD1 599, EIC=598.7:599.7 (D:\1\Data\2021\2021-10\SEQUENCE 2021-10-21 15-40-46\001-P2-B1-bh 1184 hplc v 86.D) ES-AP
mAU
min

1.2.24 S17: C5-Methoxyphenylpiperazine Chloride C-5

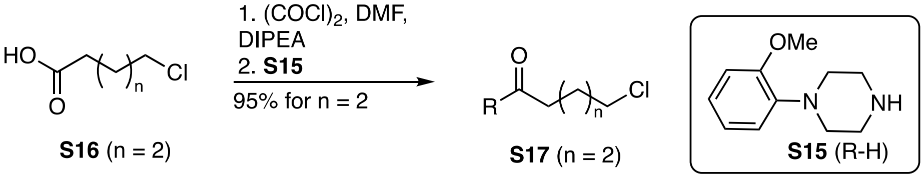

S17 has previously been used (*J. Med. Chem.* **1997**, *40*, 2653–2656).

In a 5 mL rbf, 5-chlorovaleric acid **S16** (0.328 mL, 2.81 mmol, 1.20 equiv.) and DMF (1 drop) were dissolved in THF (9.5 mL). The mixture was cooled in an ice-water bath, and then oxalyl chloride (0.280 mL, 3.28 mmol, 1.40 equiv.) was added dropwise under evolution of bubbles. The mixture was warmed to room temperature and stirred for another 30 min. The solvent was removed under reduced pressure and the yellow oily residue taken up in THF (1 mL). In a separate 5 mL rbf, 1-(2-methoxyphenyl)piperazine (450 mg, 2.34 mmol, 1.00 equiv.), DIPEA (0.815 mL, 4.68 mmol, 2.00 equiv.), and DMAP (1 grain) were dissolved in THF (8.0 mL), and cooled in an ice-water bath. The acyl chloride solution was added dropwise under evolution of fumes. After 30 seconds, a white precipitate occurred. The reaction was stirred at room temperature for 10 min, then the solvent removed under reduced pressure. The crude white solid was subjected to FCC (24 g silica, DCM-5% MeOH in DCM), to yield **S17** as light-yellow oil in 95% yield (688 mg, 2.21 mmol).

R_f = 0.66 (5% MeOH in DCM; UV detection).

HRMS (ESI): calc. for $C_{16}H_{24}ClN_2O_2^+$ $[M+H]^+$: 311.1565; found 311.1567.

LCMS (5-100% MeCN in H_2O with 0.1% formic acid over 5 min) t_R = 3.265 min, 254 nm detection.

LRMS (ESI): calc. for $C_{16}H_{24}ClN_2O_2^+$ $[M+H]^+$: 311.1; found 311.1.

^{1}H NMR (400 MHz, $CDCl_3$) δ 7.06 – 7.01 (m, 1H), 6.96 – 6.84 (m, 3H), 3.88 (s, 3H), 3.80 (t, *J* = 5.1 Hz, 2H), 3.64 (t, *J* = 5.2 Hz, 2H), 3.57 (t, *J* = 6.2 Hz, 2H), 3.08 – 2.99 (m, 4H), 2.41 (t, *J* = 6.9 Hz, 2H), 1.90 – 1.77 (m, *J* = 4H).

^{13}C NMR (101 MHz, $CDCl_3$) δ 170.98, 152.38, 140.77, 123.71, 121.18, 118.56, 111.46, 55.57, 51.18, 50.74, 45.98, 44.86, 41.92, 32.44, 32.29, 22.68.

IR (neat) 3406 (b), 2940 (w), 2832 (w), 1636 (m), 1594 (m), 1499 (s), 1436 (m), 1369 (w), 1236 (s), 1152 (m), 1118 (m), 1056 (s), 939 (m), 747 (m) cm^{-1}.

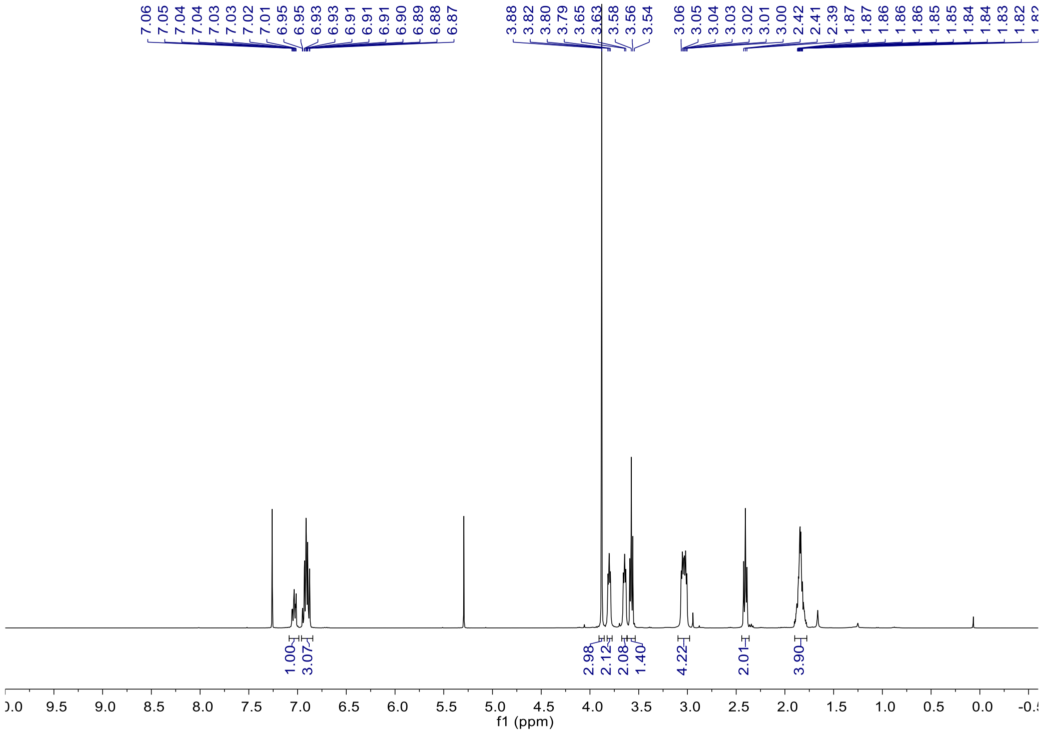
7.06
7.05
7.04
7.04
7.03
7.03
7.02
7.01
6.95
6.95
6.93
6.93
6.91
6.91
6.91
6.90
6.89
6.88
6.87
3.88
3.82
3.80
3.79
3.65
3.63
3.58
3.56
3.54
3.06
3.05
3.04
3.03
3.02
3.01
3.00
2.42
2.41
2.39
1.87
1.87
1.86
1.86
1.86
1.85
1.85
1.84
1.84
1.83
1.82
1.00
3.07
2.98
2.12
2.08
1.40
4.22
2.01
3.90
9.5
9.0
8.5
8.0
7.5
7.0
6.5
6.0
5.5
5.0
4.5
4.0
3.5
3.0
2.5
2.0
1.5
1.0
0.5
0.0
f1 (ppm)

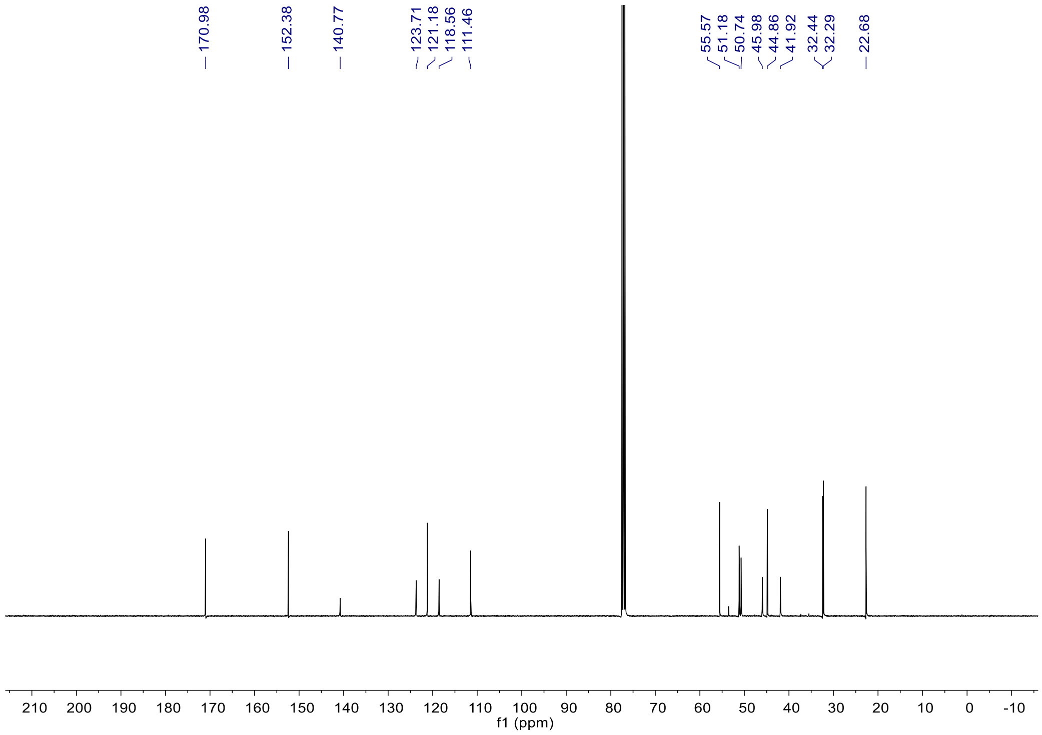
170.98
152.38
140.77
123.71
121.18
118.56
111.46
55.57
51.18
50.74
45.98
44.86
41.92
32.44
32.29
22.68
210
200
190
180
170
160
150
140
130
120
110
100
90
80
70
60
50
40
30
20
10
0
-10
f1 (ppm)

1.2.25 S18: C5-Methoxyphenylpiperazine Amide Azo C-5

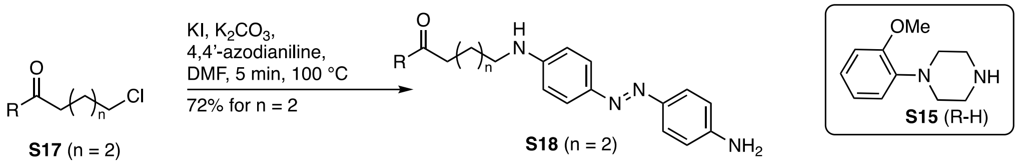

In a 20 mL scintillation vial, **S17** (202 mg, 0.650 mmol, 1.00 equiv.), KI (324 mg, 1.950 mmol, 3.00 equiv.) and 4,4'-azodianiline (138 mg, 0.650 mmol, 1.00 equiv.) were dissolved in DMF (3.2 mL), and the orange reaction mixture heated to 60 °C. After 3h, the dark green reaction was judged complete by LCMS analysis. The solvent was removed under reduced pressure at 50 °C. The residue was taken up in DCM and washed with LiCl (10 %, aq., 5x). The organic phase was dried over Na_2SO_4, filtered, and concentrated under reduced pressure. The red residue was subjected to FCC (4g silica, 0 -> 5% MeOH in DCM), to yield the product **S18** as dark orange oil in 72% yield (226 mg, 0.464 mmol).

R_f = 0.46 (5% MeOH in DCM; red spot).

HRMS (ESI): calc. for $C_{28}H_{35}N_6O_2^+$ [M+H]$^+$: 487.2843; found 487.2846.

LCMS (5-100% MeCN in H_2O with 0.1% formic acid over 5 min) t_R = 3.492 min, 360 nm detection.

LRMS (ESI): calc. for $C_{28}H_{35}N_6O_2^+$ [M+H]$^+$: 487.3; found 487.3.

^{1}H NMR (400 MHz, Chloroform-*d*) δ 7.82 – 7.68 (m, 4H), 7.03 (m, 1H), 6.98 – 6.84 (m, 3H), 6.81 – 6.68 (m, 2H), 6.67 – 6.61 (m, 2H), 3.88 (s, 3H), 3.81 (t, *J* = 5.1 Hz, 2H), 3.64 (t, *J* = 5.2, 2H), 3.24 (t, *J* = 6.6 Hz, 2H), 3.04 – 3.01 (m, 4H), 2.43 (t, *J* = 7.1 Hz, 2H), 1.86 – 1.69 (m, 4H).

^{13}C NMR (101 MHz, $CDCl_3$) δ ^{13}C NMR (101 MHz, Chloroform-*d*) δ 171.21, 152.38, 150.28, 148.33, 146.08, 144.85, 140.75, 124.66, 124.28, 123.71, 121.20, 118.58, 114.95, 112.34, 111.45, 55.58, 51.17, 50.75, 45.96, 43.53, 41.98, 32.82, 29.10, 22.72.

IR (neat) 3342 (b), 2926 (w), 1623 (m), 1593 (s), 1446 (w), 1334 (w), 1301 (w), 1240 (m), 1147 (m), 1027 (m), 836 (w), 734 (w) cm^{-1}.

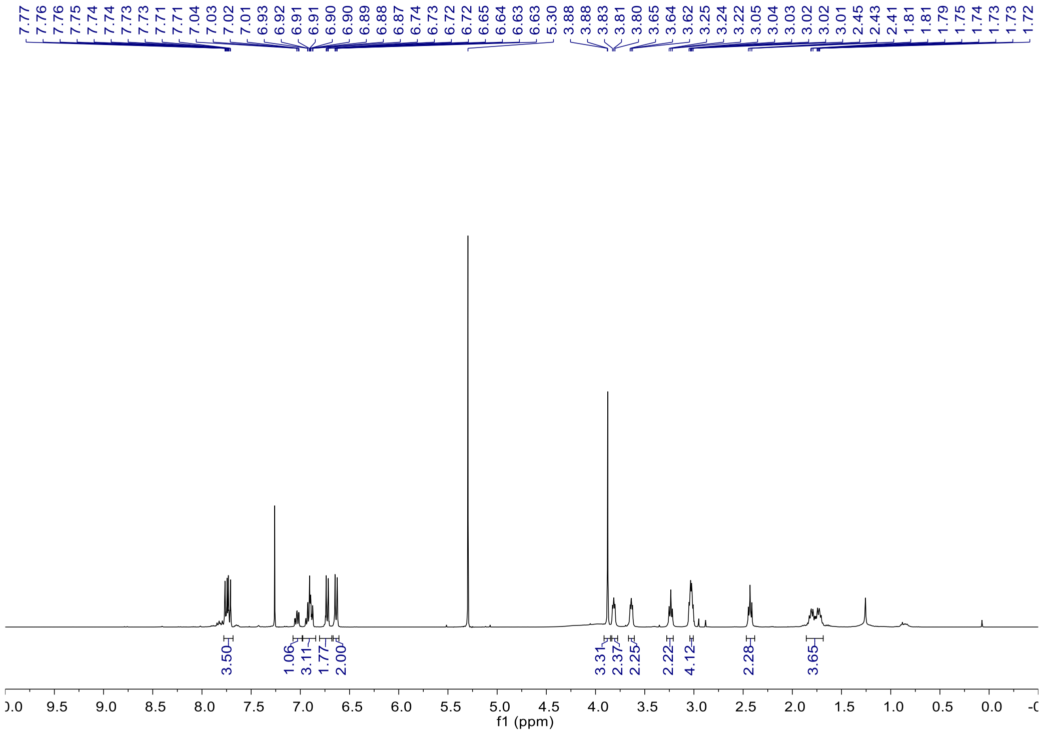
7.77 7.76 7.75 7.74 7.74 7.73 7.73 7.71 7.71 7.04 7.03 7.02 7.01 6.93 6.92 6.91 6.91 6.90 6.90 6.89 6.88 6.87 6.74 6.73 6.72 6.72 6.65 6.64 6.63 6.63 5.30 3.88 3.88 3.83 3.81 3.80 3.65 3.64 3.62 3.25 3.24 3.22 3.05 3.04 3.03 3.02 3.02 3.01 2.45 2.43 2.41 1.81 1.81 1.79 1.75 1.74 1.73 1.73 1.72
3.50 1.06 3.11 1.77 2.00 3.31 2.37 2.25 2.22 4.12 2.28 3.65
f1 (ppm)

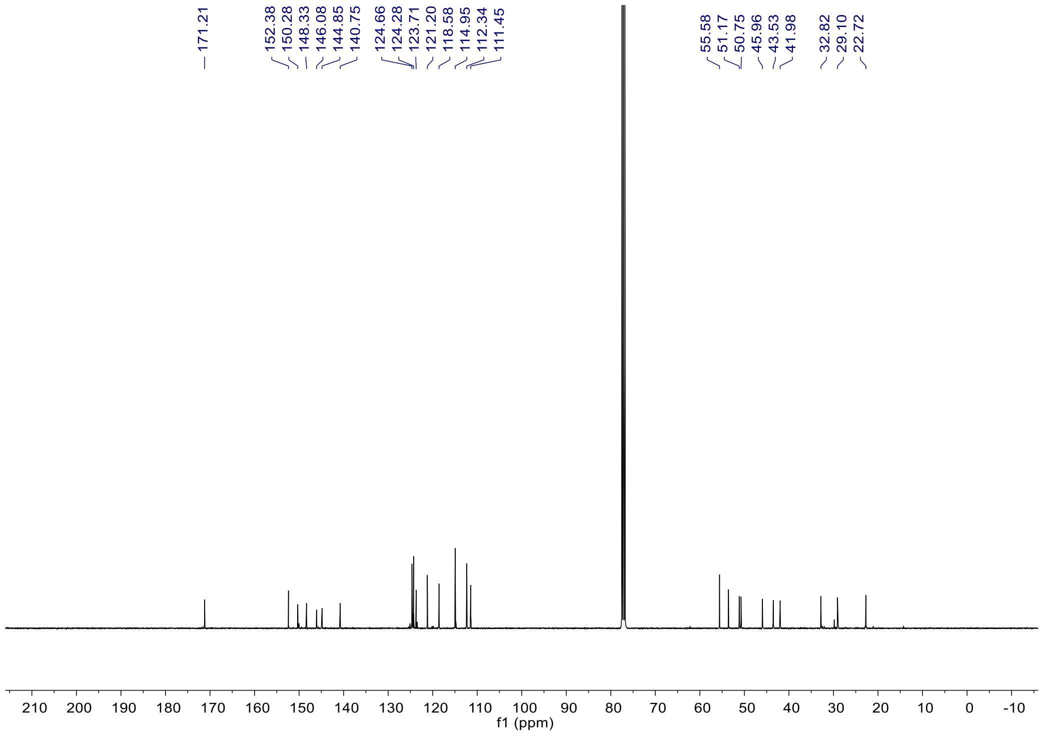
171.21
152.38 150.28 148.33 146.08 144.85 140.75
124.66 124.28 123.71 121.20 118.58 114.95 112.34 111.45
55.58 51.17 50.75 45.96 43.53 41.98
32.82 29.10 22.72
f1 (ppm)

1.2.26 S19: C5-Methoxyphenylpiperazine Amine Azo C-5

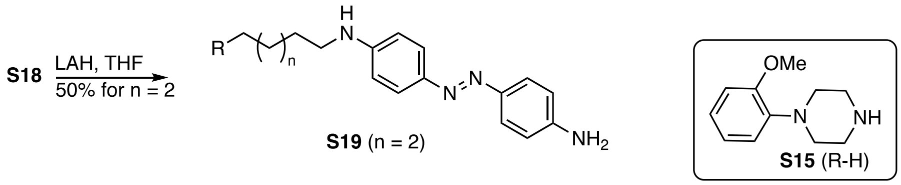

In a 25 mL rbf, LAH (1M in THF, 3.1 mL, 3.058 mmol, 8.00 equiv.) was cooled in an ice-water bath. **S18** (186 mg, 0.382 mmol, 1.00 equiv., as solution in 2 mL THF) was added dropwise. The dark red solution was warmed to room temperature and stirred for another 30 min. Then, the solution was cooled in an ice-water bath, and NaOH (5 mL, 2M) was added slowly under evolution of bubbles. Then, DCM was added, and the phases separated. The organic phase was dried over Na_2SO_4, filtered, and concentrated under reduced pressure. The red oil was subjected to FCC (4g silica, 0 -> 5% MeOH in DCM) to yield **S19** in 50% yield (90 mg, 0.190 mmol).

R*f* = 0.44 (5% MeOH in DCM; red spot).

HRMS (ESI): calc. for $C_{28}H_{37}N_6O^+$ [M+H]$^+$: 473.3126; found 473.3129.

LCMS (5-100% MeCN in H_2O with 0.1% formic acid over 5 min) t_R = 2.984 min, 360 nm detection.

LRMS (ESI): calc. for $C_{28}H_{37}N_6O^+$ [M+H]$^+$: 473.3; found 473.3.

^{1}H NMR (400 MHz, Chloroform-*d*) δ 7.86 – 7.67 (m, 4H), 7.02 – 6.84 (m, 4H), 6.75 – 6.71 (m, 2H), 6.68 – 6.60 (m, 2H), 3.86 (s, 3H), 3.21 (t, *J* = 7.1 Hz, 2H), 3.16 – 3.09 (m, 4H), 2.74 – 2.65 (m, 4H), 2.47 (t, *J* = 7.7 Hz, 2H), 1.71 – 1.62 (m, 4H), 1.49 (m, 2H).

^{13}C NMR (101 MHz, Chloroform-*d*) δ 152.39, 150.32, 148.33, 146.13, 144.91, 141.35, 124.65, 124.49, 124.30, 123.13, 121.15, 118.39, 114.96, 114.92, 112.34, 111.29, 58.67, 55.50, 53.59, 50.60, 43.74, 29.85, 29.46, 26.62, 25.20.

IR (neat) 3340 (b), 2929 (w), 2820 (w), 1592 (s), 1499 (m), 1449 (w), 1334 (w), 1238 (w), 1145 (m), 1026 (w), 834 (w), 734 (w) cm^{-1}.

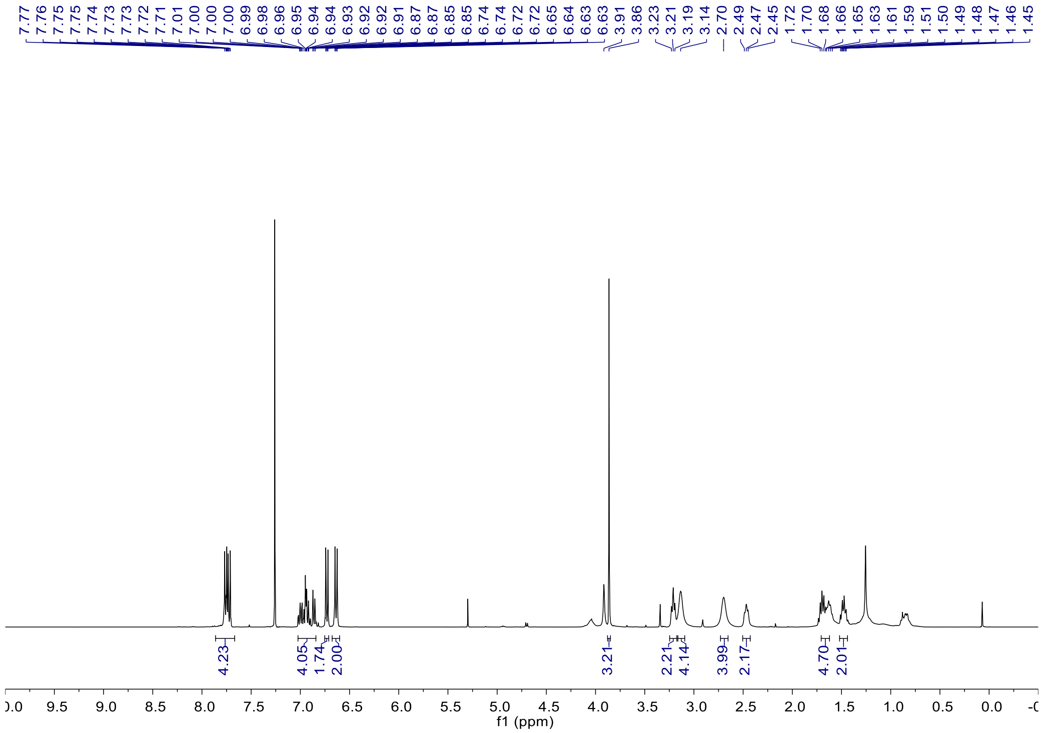

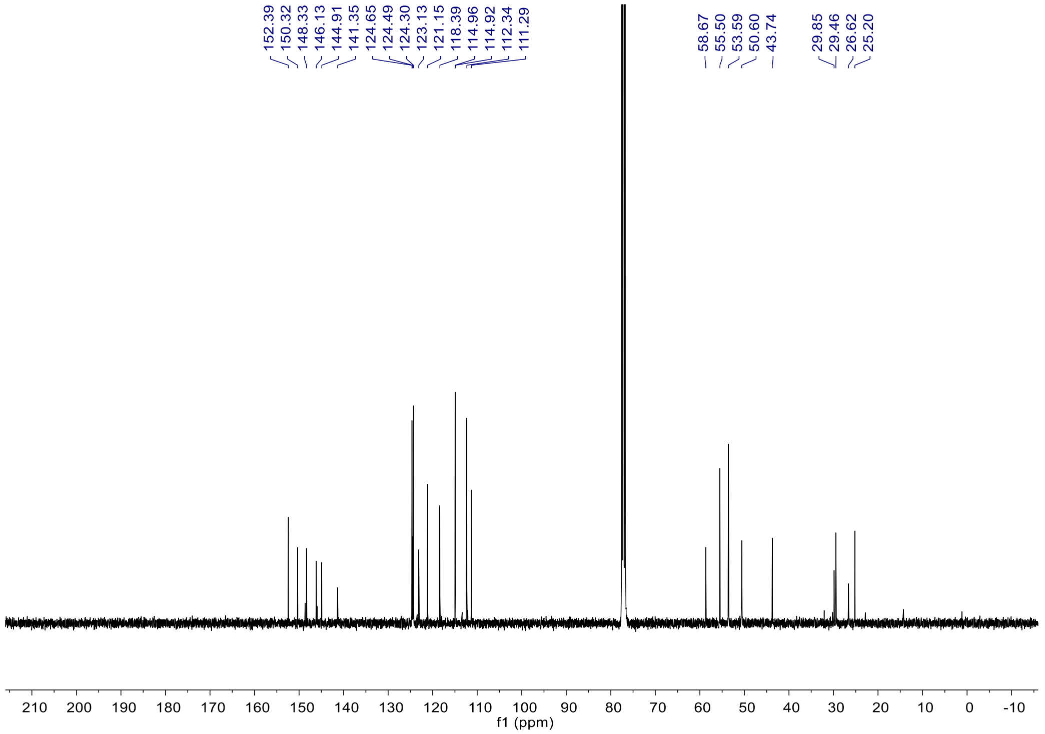

1.2.27 S20: C5-Methoxyphenylpiperazine Azo PEG

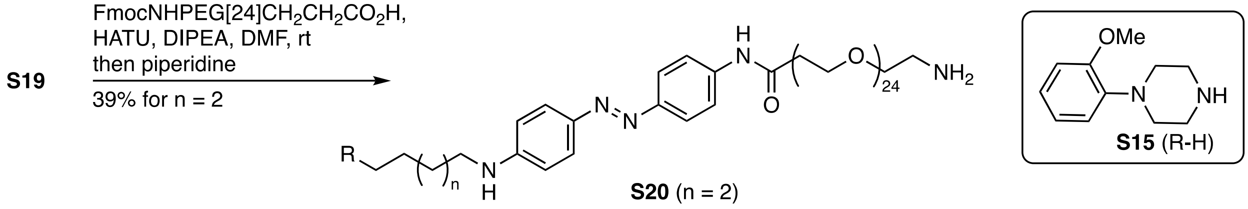

In a 20 mL vial, **S19** (20.2 mg, 0.043 mmol, 1.00 equiv.) and FmocHNPEG[24]CH_2CH_2COOH (58.5 mg, 0.043 mmol, 1.00 equiv.) were dissolved in DMF (1 mL), and DIPEA (37 uL, 0.214 mmol, 5.00 equiv.) was added. Then, HATU (17.1 mg, 0.043 mmol, 1.00 equiv.) was added in one portion. After 10h, LCMS showed full conversion to the desired product. Piperidine (85 uL) was added. After LCMS confirmed complete deprotection, the crude reaction solution was diluted with MeCN and treated with AcOH (200 uL), and subjected to RP-HPLC purification (15-42% MeCN in water containing 0.1% FA, 10 mL/min, 8 min runtime, semi prep column, 360 nm detection, t_R = 7.394 min). After evaporation of the solvent under reduced pressure, **S20** was obtained in 39% yield as orange oil (27.0 mg, 0.017 mmol).

<u>Fmoc Intermediate</u>:

LCMS (5-100% MeCN in H_2O with 0.1% formic acid over 5 min) t_R = 3.767 min, 360 nm detection.

LRMS (ESI): calc. for $C_{94}H_{149}N_7O_{28}^{2+}$ $[M+2H]^{2+}$: 912.5; found 912.4.

<u>Product</u>:

HRMS (ESI): calc. for $C_{79}H_{139}N_7O_{26}^{2+}$ $[M+2H]^{2+}$: 801.4789; found 801.4792.

LCMS (5-100% MeCN in H_2O with 0.1% formic acid over 5 min) t_R = 2.915 min, 360 nm detection.

LRMS (ESI): calc. for $C_{79}H_{139}N_7O_{26}^{2+}$ $[M+2H]^{2+}$: 534.3; found 534.5.

^{1}H NMR (400 MHz, Chloroform-*d*) δ 7.83 – 7.68 (m, 6H), 7.06 – 6.81 (m, 4H), 6.64 (d, *J* = 8.4 Hz, 2H), 3.87 – 3.83 (m, 5H), 3.82 – 3.78 (m, 3H), 3.69 – 3.58 (m, 90H), 3.25 – 3.12 (m, 8H), 2.90 – 2.82 (m, 4H), 2.68 (t, *J* = 5.6 Hz, 2H), 2.64 – 2.58 (m, 2H), 1.75 – 1.65 (m, 4H), 1.52 – 1.44 (m, 2H).
^{13}C NMR (101 MHz, Chloroform-*d*) δ 170.31, 152.32, 150.94, 149.32, 144.73, 140.76, 140.04, 125.17, 123.49, 123.16, 121.19, 120.08, 118.51, 112.25, 111.32, 70.68, 70.66, 67.29, 58.04, 55.52, 53.00, 49.74, 43.49, 38.18, 29.20, 25.64, 24.98.
IR (neat) 3398 (b), 2917 (m), 1600 (s), 1540 (m), 1501 (w), 1454 (w), 1348 (m), 1301 (w), 1244 (w), 1094 (s), 949 (w), 833 (m), 720 (w) cm^{-1}.

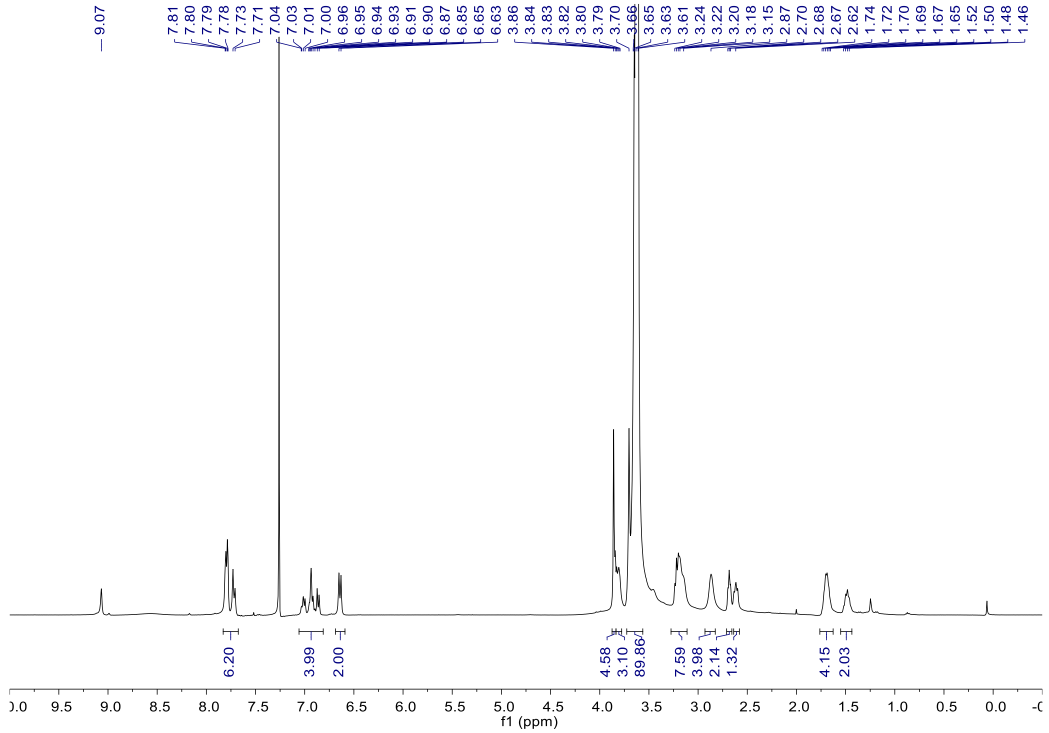
9.07
7.81
7.80
7.79
7.78
7.73
7.71
7.04
7.03
7.01
7.00
6.96
6.95
6.94
6.93
6.91
6.90
6.87
6.85
6.65
6.63
3.86
3.84
3.83
3.82
3.80
3.79
3.70
3.66
3.65
3.63
3.61
3.24
3.22
3.20
3.18
3.15
2.87
2.70
2.68
2.67
2.62
1.74
1.72
1.70
1.69
1.67
1.65
1.52
1.50
1.48
1.46
6.20
3.99
2.00
4.58
3.10
89.86
7.59
3.98
2.14
1.32
4.15
2.03
f1 (ppm)

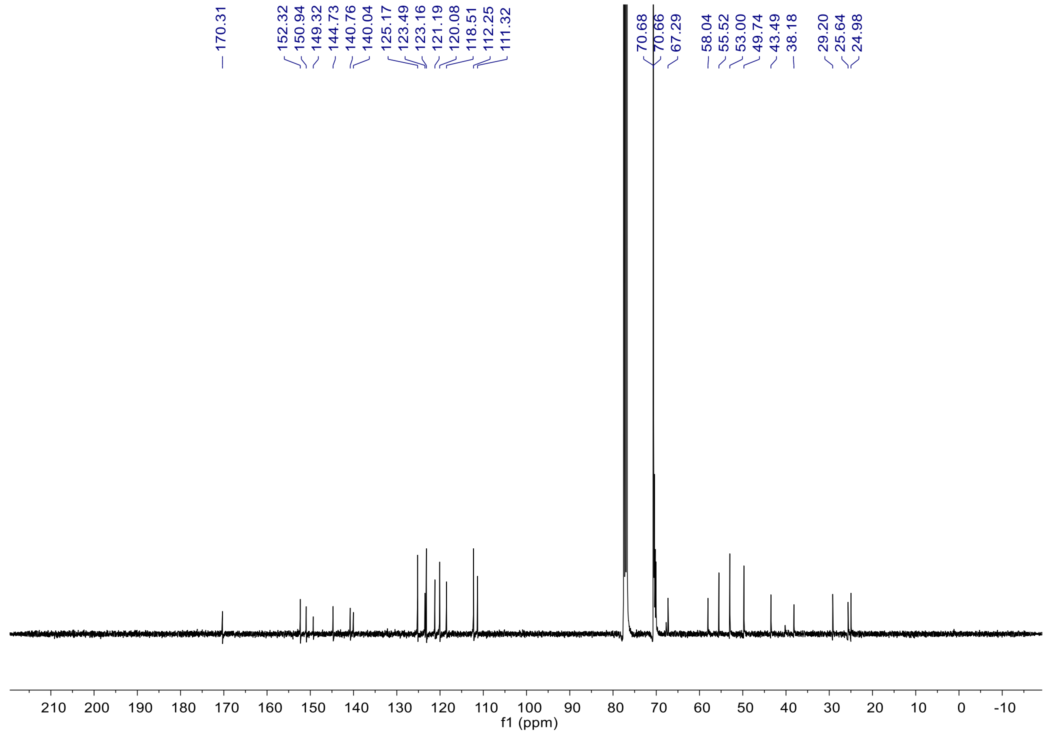
170.31
152.32
150.94
149.32
144.73
140.76
140.04
125.17
123.49
123.16
121.19
120.08
118.51
112.25
111.32
70.68
70.66
67.29
58.04
55.52
53.00
49.74
43.49
38.18
29.20
25.64
24.98
f1 (ppm)

1.2.28 P-D2$_{block}$(C5)

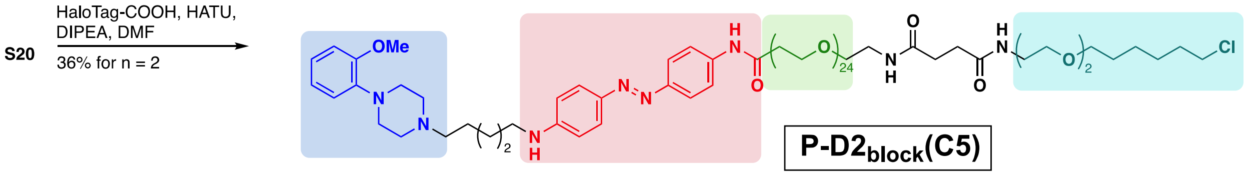

In a 4 mL vial, **S20** (6.5 mg, 4.1 umol, 1.00 equiv.) and HaloTag-COOH (1.4 mg, 4.1 umol, 1.00 equiv.) were dissolved in DMF (100 uL), and DIPEA (3 uL, 17 umol, 4 equiv.) was added. Then, HATU (1.6 mg, 4.1 umol, 1.00 equiv.) was added in one portion. After 10h, LCMS showed full conversion to the desired product. The crude reaction solution was diluted with MeCN and treated with AcOH (20 uL) and subjected to RP-HPLC purification (15-60% MeCN in water containing 0.1% FA, 10 mL/min, 8 min runtime, semiprep column, 360 nm detection, t_R = 6.545 min). After evaporation of the solvent under reduced pressure, **P-D2$_{block}$(C5)** was obtained in 36% yielded as orange oil (2.8 mg, 1.5 umol).

HRMS (ESI): calc. for $C_{93}H_{162}ClN_8O_{30}Na_2^{2+}$ $[M+2Na]^{2+}$: 976.0435; found 976.0636.

LCMS (5-100% MeCN in H_2O with 0.1% formic acid over 5 min) t_R = 3.459min, 360 nm detection.

LRMS (ESI): calc. for $C_{93}H_{164}ClN_8O_{30}^{2+}$ $[M+2H]^{2+}$: 954.0; found 954.1

^{1}H NMR (400 MHz, Methanol-d_4) δ 7.74 (dd, *J* = 8.7, 4.1 Hz, 6H), 7.01 – 6.90 (m, 4H), 6.70 (d, *J* = 9.0 Hz, 2H), 3.86 – 3.83 (m, 5H), 3.63 – 3.51 (m, 120H), 3.48 (t, *J* = 6.6 Hz, 2H), 3.34 (t, *J* = 5.6 Hz, 4H), 3.22 (t, *J* = 6.9 Hz, 2H), 3.08 (s, 5H), 2.71 (s, 5H), 2.67 (s, 2H), 2.48 (s, 6H), 1.80 – 1.68 (m, 4H), 1.67 – 1.55 (m, 2H), 1.54 – 1.36 (m, 4H).

^{13}C NMR (101 MHz, Methanol-d_4) δ 174.65, 172.42, 153.89, 153.59, 150.68, 145.05, 142.09, 141.14, 126.20, 124.72, 123.73, 122.19, 121.24, 119.44, 112.85, 112.81, 72.23, 71.53, 71.52, 71.30, 71.28, 71.20, 70.57, 68.21, 59.68, 55.95, 54.31, 51.40, 45.73, 44.06, 40.45, 40.43, 38.76, 33.76, 32.26, 30.55, 30.06, 27.75, 27.13, 26.50, 26.15.

IR (neat) 3328 (b), 2865 (m), 1649 (w), 1599 (s), 1537 (m)., 1454 (w), 1347 (w), 1300 (w), 1241 (m), 1095 (s), 949 (w), 847 (w) cm^{-1}.

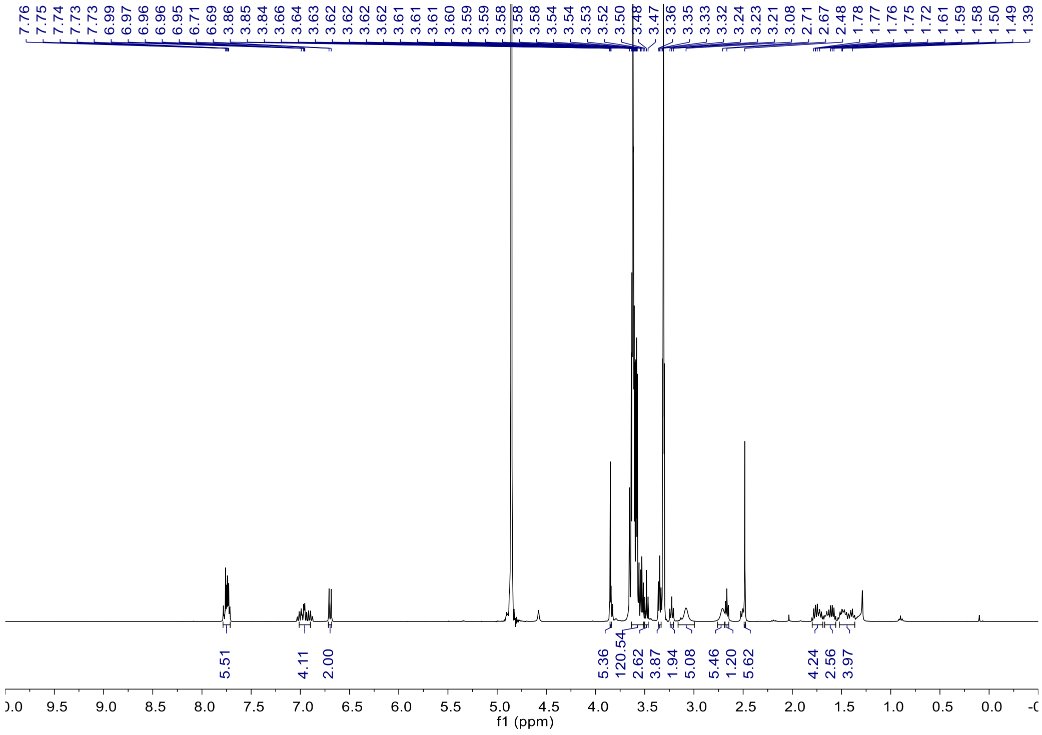
7.76 7.75 7.74 7.73 7.73 6.99 6.97 6.96 6.96 6.95 6.71 6.69 3.86 3.85 3.84 3.66 3.64 3.63 3.62 3.62 3.62 3.61 3.61 3.61 3.60 3.59 3.59 3.58 3.58 3.58 3.54 3.54 3.53 3.52 3.50 3.48 3.47 3.36 3.35 3.33 3.32 3.32 3.24 3.23 3.21 3.08 2.71 2.67 2.48 1.78 1.77 1.76 1.75 1.72 1.61 1.59 1.58 1.50 1.49 1.39
5.51 4.11 2.00 5.36 120.54 2.62 3.87 1.94 5.08 5.46 1.20 5.62 4.24 2.56 3.97
f1 (ppm)

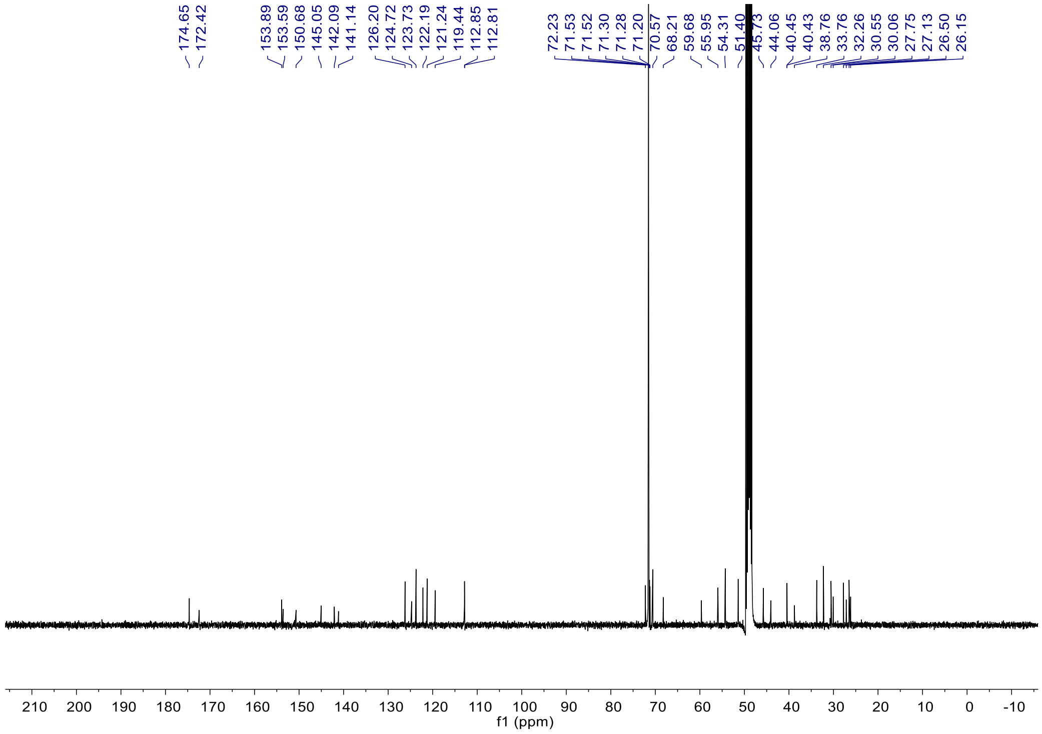
174.65 172.42 153.89 153.59 150.68 145.05 142.09 141.14 126.20 124.72 123.73 122.19 121.24 119.44 112.85 112.81 72.23 71.53 71.52 71.30 71.28 71.20 70.57 68.21 59.68 55.95 54.31 51.40 45.73 44.06 40.45 40.43 38.76 33.76 32.26 30.55 30.06 27.75 27.13 26.50 26.15
f1 (ppm)
